# A Direct Method for Synthesis of Quinoxalines and Quinazolinones Using Epoxides as Alkyl Precursor

**DOI:** 10.3390/molecules28217391

**Published:** 2023-11-02

**Authors:** Xueyan Lv, Lili Lv, Shichen Li, Chengcheng Ding, Bingchuan Yang, Chen Ma

**Affiliations:** 1 Key Laboratory of Special Functional Aggregated Materials, Ministry of Education, School of Chemistry and Chemical Engineering, Shandong University, Jinan 250100, China; lxy17852267584@163.com (X.L.); ray19940519@foxmail.com (S.L.); 202220349@mail.sdu.edu.cn (C.D.); 2 China Petroleum Planning and Engineering Institute, Dongying 257237, China; lvlili@petrochina.com.cn; 3 College of Chemistry and Chemical Engineering, Qilu Normal University, Jinan 250013, China

**Keywords:** epoxide, pyrrolo [1,2-*a*]quinoxalines, quinazolin-4-one, transition-metal free

## Abstract

An iodine-mediated one-pot synthesis of pyrrolo/indolo [1,2-*a*]quinoxalines and quinazolin-4-one via utilizing epoxides as alkyl precursors under metal-free conditions has been described. Both 1-(2-aminophenyl)-pyrrole and 2-aminobenzamide could be applied to this protocol. A total of 33 desired products were obtained with moderate to good yields. This methodology was suitable for wide-scale preparation and the obtained products could be further modified into promising pharmaceutically active reagents.

## 1. Introduction

**Figure 1:**
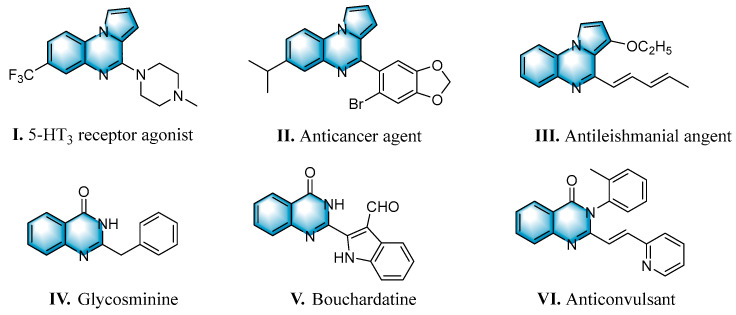
Drug Molecules Containing Quinoxalines and Quinazolines.

**Scheme 1:**
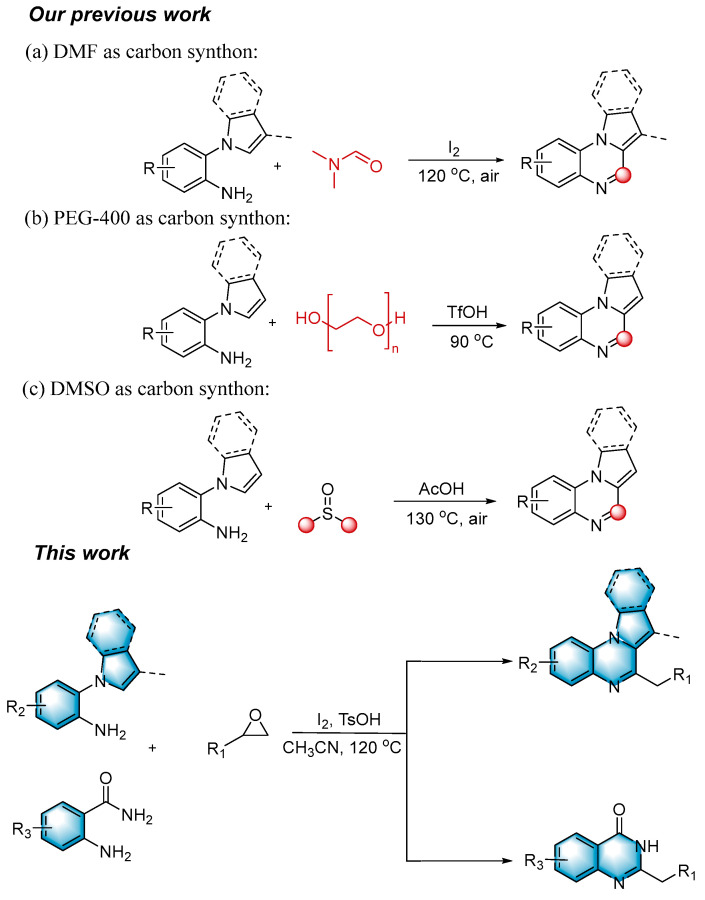
Synthesis of Pyrrolo[1,2-*a*]quinoxalines.

*N*-heterocycles are inseparable from our life for use as drug molecules, functional materials and dyes [1,2,3]. Pyrrolo [1,2-*a*]quinoxalines and quinazolin-4-ones are two essential heterocyclic skeletons with outstanding biological properties for antimalarial, anticancer, anti-HIV, antibacterial, etc [4,5,6,7,8,9,10,11,12,13,14]. As shown in Figure 1, compound **I** exhibits excellent activity towards the 5-HT_3_ receptor, and compounds **II** and **III** have anticancer and antileishmanial properties, respectively. Glycosminine (Figure 1, **IV**) and alkaloid bouchardatine (Figure 1, **V**) are present in natural products. Compound **VI** has anticonvulsant activity. Given their excellent applications, especially in medicinal chemistry, their efficient and green synthesis has been a long-pursued research topic in the field of organic synthetic chemistry. In 1966, Cheeseman and Tuck [15] first synthesized pyrrolo [1,2-*a*]quinoxalines using 1-(2-aminophenyl)-pyrrole as the starting material with aqueous formic acid under reflux conditions. Since then, a number of groups have made tremendous efforts to construct these compounds [16,17,18,19,20,21,22,23,24,25]. In 2022, Ma [16] reported a Cu(II)-catalyzed synthesis of pyrrolo[1,2-*a*]quinoxalines using *N*,*N*-dimethylethanolamine (DMEA) as a C1 synthon. In addition, the method of Ma’s group was also applicable to the synthesis of quinazolin-4-one and benzo[4,5]imidazoquinazoline. During the preparation of our manuscript, by means of an I_2_-DMSO synergistic system, Lin [17] reported a method for the synthesis of acyl-substituted pyrrolo[1,2-*a*]quinolines using epoxides as acyl precursors. As part of our research interest in the green synthesis of nitrogen-containing heterocycles under metal-free conditions, PEG-400 [18], DMF [19] and DMSO [20] as carbon synthons have been reported (Scheme 1).

On the other hand, the synthesis of quinazolin-4-ones has also attracted much attention from chemists. In traditional synthesis methods, quinazolinone was obtained by an acid/base facilitated condensation reaction of esters, aldehydes or carboxylic acids with amides by means of some homogeneous catalytic systems and expensive raw materials [26,27,28,29,30]. In 2018, Wang [31] reported a novel synthetic strategy for the synthesis of quinazolinones from olefins, carbon monoxide and amines over a heterogeneous Ru cluster/cerium oxide catalyst under acid/ base-free and oxidant-free conditions with H_2_O as the only by-product. In 2019, Zheng [32] developed a visible light-mediated intramolecular C-N cross-coupling reaction to synthesize a series of fused *N*-substituted polycyclic quinazolinone derivatives under mild reaction conditions via long-lived photoactive photoisomer complexes. In 2023, Fan [33] reported a method for the synthesis of 5*H*-phthalazino[1,2-*b*]quinazolin-8(6*H*)-one derivatives via a *t*-BuOK-catalyzed intramolecular hydrogen amination reaction of quinazolinones. Also in 2023, Zhu [34] reported a cobalt homeostatic catalysis system for the synthesis of quinazolinones from the coupling of enaminones and oxadiazolones.

Epoxides are well-known electrophiles that can react with various nucleophiles. They are readily available from olefins or ketones and are usually air-stable and easily stored. The Meinwald rearrangement [35] is an acid-catalyzed rearrangement reaction in which epoxides form aldehydes or ketones via ring-opening followed by a 1,2-shift of the hydride or alkyl group. Aldehydes, especially enolizable aliphatic aldehydes, are susceptible to self-condensation which makes them unstable and hard to store in pure form. Therefore, it is more desirable to utilize epoxides to replace the original aldehydes. In 2023, Moran [36] synthesized a series of functionalized isochromans using epoxides as an alternative to aldehydes. In 2021, Feng [37] reported the first catalytic asymmetric multi-insertion olefin addition reaction triggered by an epoxy-vinyl Meinwald rearrangement using a chiral *N*,*N*′-dioxide/Sc^III^ complex catalyst. Encouraged by these discoveries and to further explore the applications of epoxides [38,39,40,41,42], herein, we developed a method for synthesizing pyrrolo[1,2-*a*]quinoxalines and quinazolin-4-ones via a tandem Meinwald rearrangement and cyclization in one pot.

## 2. Results and Discussion

### 2.1. Optimization

We commenced our exploration with 1-(2-aminophenyl)-pyrrole (**1a**; 0.5 mmol) and styrene oxide (**2a**; 1.0 mmol) as the model substrates. In the presence of I_2_ (0.5 mmol) and AcOH (0.5 mmol) in CH_3_CN (1.0 mL) at 120 °C, the target product 4-benzylpyrrolo[1,2-*a*]quinoxaline (**3a**) was obtained in 52% yield. Then, we screened several common Brønsted acids, such as HCOOH, trifluoromethanesulfonic acid (TfOH) and *p*-toluenesulfonic acid (TsOH) (Table 1, entries 1–5), and **3a** had the best yield (76%) when TsOH was used (Table 1, entry 5). In addition, we found that in the absence of iodine or acid, yields showed a significant decline (Table 1, entries 6–7). It can be noted that iodine and acid play a crucial role in this reaction system. Furthermore, when the acid was reduced to 0.5 equivalents, the yield decreased; when the acid equivalent was increased to 1.5 equivalents, the yield did not increase significantly (Table 1, entries 8–9). In addition, common solvents, such as 1,2-dichloroethane (DCE), EtOH, toluene, *N*-methylpyrrolidone (NMP) and PhCl, were selected, and CH_3_CN was the best (Table 1, entries 10–14). Lowering the reaction temperature did not improve the reaction yield (Table 1, entries 15–16). For the sake of economy and safety, we did not raise the temperature further. We finally determined that subsequent experiments will be performed with I_2_ (0.5 mmol) and TsOH (0.5 mmol) in CH_3_CN at 120 °C for 4 h (Table 1, entry 5).

### 2.2. Scope of Substrates

With the optimal reaction conditions in hand, various substituted 1-(2-aminophenyl)-pyrroles were first explored, and the results are shown in Table 2. For monosubstituted 1-(2-aminophenyl)-pyrroles, some bearing electron-donating groups (EDGs), like methyl, methoxy or *tert*-butyl, work well in this method (**3b**–**3c**, **3e**–**3f** and **3k**). The method was also compatible with substrates containing electron-withdrawing groups (EWGs), such as fluoro, chloro, bromo and iodine (**3d**, **3g**–**3j** and **3l**). Disubstituted 1-(2-aminophenyl)-pyrroles were also well tolerated under these conditions; for 4,5-dimethyl-2-(1*H*-pyrrol-1-yl)aniline, 4,5-difluoro-2-(1*H*-pyrrol-1-yl)aniline and 4,5-dichloro-2-(1*H*-pyrrol-1-yl)aniline, the target products **3m**, **3n** and **3o** were obtained with the yields of 64%, 76% and 65%, respectively. When we replaced the pyrrole ring with indole, the reaction still proceeded with 35–48% yields (**3p**–**3r**). The reaction also proceeds smoothly when alkyl epoxides, such as 1,2-epoxybutane, were used (**3s**); when 3,4-epoxy-1-butene was used, the product **3t’** was expected to suffer a facile double-bond migration, affording the conjugated structure **3t**.

Inspired by the experimental results in Table 2, we then turned our focus to the synthesis of quinazolin-4-ones. We attempted to react 2-aminobenzamide (**4a**) with styrene oxide (**2a**) under optimal conditions and successfully obtained 2-benzylquinazolin-4(3*H*)-one (**5a**) in 70% yield (Table 3). Whether the R_3_ group of 2-aminobenzamide was EDG or EWG participated successfully in the reaction with yields ranging from 58% to 74% (5a-5j). Whether the R_3_ group of the 2-aminobenzamide was an EDG or EWG and could smoothly participate in this reaction, were demonstrated with yields ranging from 58% to 74% (**5a**–**5j**). The reaction also performed well when styrene oxide (**2a**) was attached to EWGs, such as chloro and bromo (**5k**–**5l**). When we used 3,4-epoxy-1-butene to react with **4a**, the expected product quinazolinone **5m’** suffered an easy double-bond migration to give the conjugated product **5m**. Unfortunately, we did not obtain the desired products **5n** and **5o** when the R_4_ group was replaced with an alkyl or aryl group.

### 2.3. Large Scale Reaction and Synthetic Applications

**Scheme 2:**
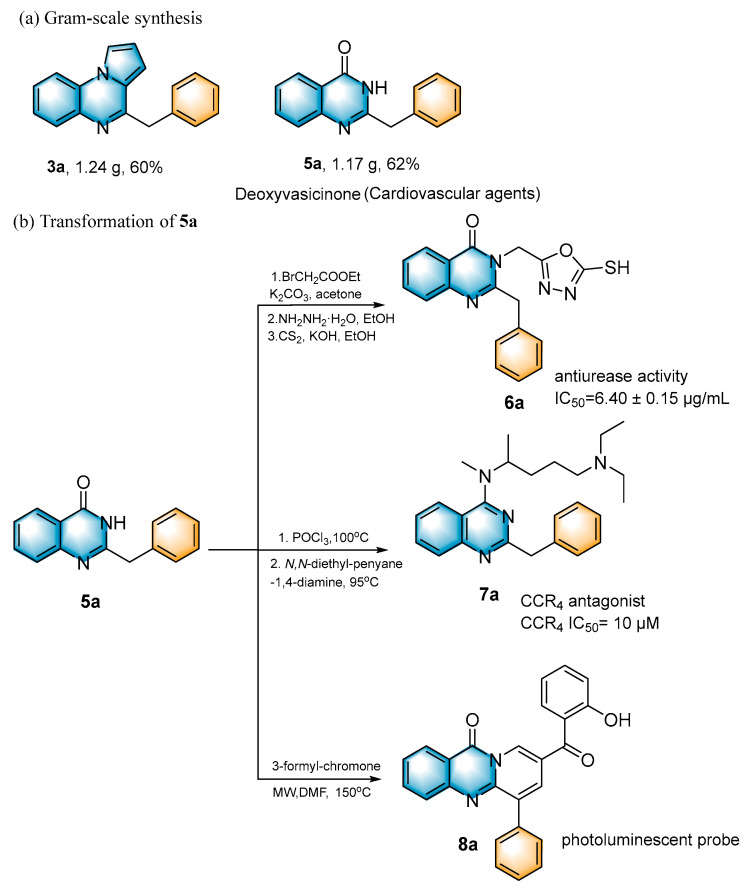
Large-scale reaction and synthetic applications.

Subsequently, several synthetic applications were performed to demonstrate the practicality of this methodology (Scheme 2). Under standard conditions, the gram-scale synthesis of **3a** and **5a** was carried out. Pleasantly, the desired product was successfully obtained in 60% and 62% yields, respectively, making the procedure suitable for a broad-scale preparation. Of these, **5a** can be used as a cardiovascular agent [14]. In addition, the 2-benzylquinazolin-4(3*H*)-one (**5a**) obtained in this protocol could be further modified into an active antiurease reagent [43], molecular antagonists of CCR_4_ [44] and a photoluminescent probe [45]. The photoluminescent probe could be used for the label-free, highly selective and sensitive detection of Fe^3+^ and Ag^+^ metal ions.

### 2.4. Mechanism Investigation

**Scheme 3:**
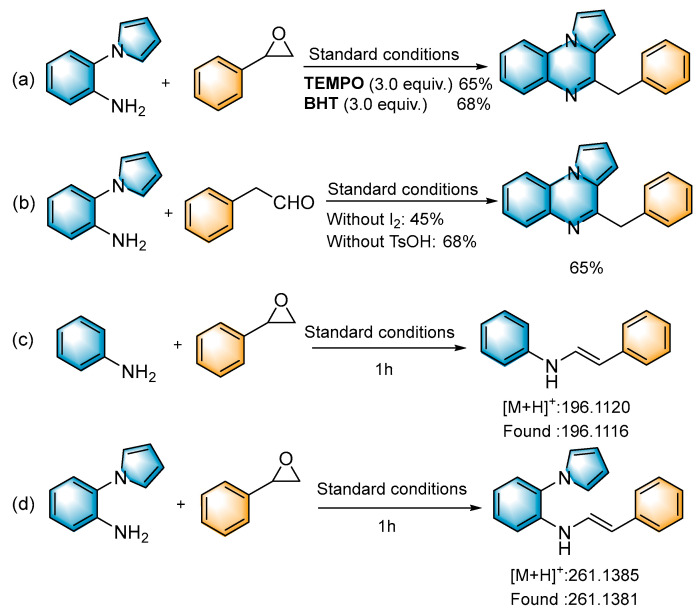
Control experiments (**a**–**d**).

To shed light on the mechanism of the reaction, several control experiments were performed (Scheme 3). When the radical scavengers 2,2,6,6-tetramethylpiperidinyl-1-oxide (TEMPO, 3.0 equiv.) and 2,6-di-*tert*-butyl-4-methylphenol (BHT, 3.0 equiv.) were added to the reaction system under standard conditions, the desired product was obtained in 65% and 68% yields (Scheme 3a), respectively. This indicated that the conversion was a non-radical process. When the reaction was carried out without the addition of I_2_, the yield exhibited a significant decrease (27%). When the reaction system was performed without the addition of TsOH, the target product was obtained with a 50% yield; we presumed that it is the I_2,_ as a Lewis acid, that plays a role in the conversion process (Table 1, entries 6–7). To investigate the reaction mechanism in more depth, we used 2-phenylacetaldehyde to react with **1a** under standard conditions to obtain the target product in 65% yield (Scheme 3b). This indicated that 2-phenylacetaldehyde may be a key intermediate in the reaction. In the absence of I_2_, the yield decreased significantly, indicating that iodine plays a key role in the subsequent cyclization reaction. Under standard conditions, aniline and 1-(2-aminophenyl)-pyrrole were reacted with styrene oxide (**2a**), respectively, and the intermediate imide was detected with HRMS (Scheme 3c,d).

**Scheme 4:**
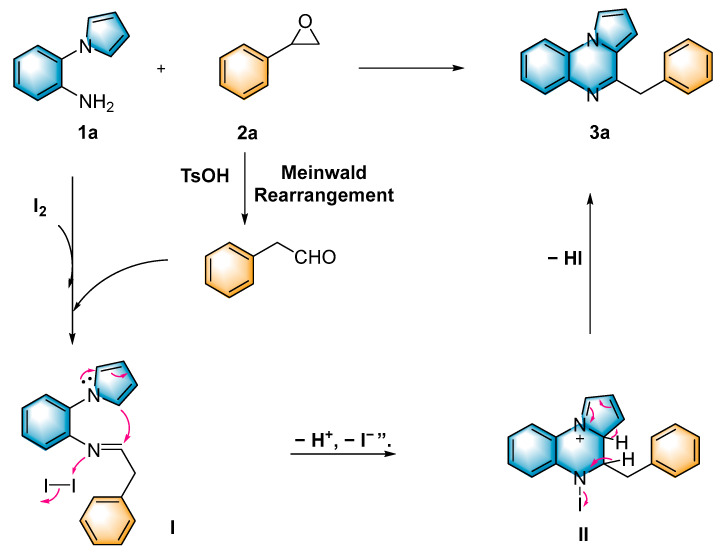
Proposed Mechanism.

Based on the aforementioned control experiments and related literature studies, a plausible mechanism of this reaction is described in Scheme 4. Initially, in the presence of TsOH, styrene oxide (**2a**) underwent a Meinwald rearrangement to afford 2-phenylacetaldehyde. The 2-phenylacetaldehyde reacted with 1-(2-aminophenyl)-pyrrole (**1a**) accompanied by the elimination of H_2_O to generate the intermediate imine **I**. Afterwards, intramolecular cyclization was accomplished with the assistance of molecular iodine to afford the dihydropyrrolo[1,2-*a*]quinoxaline **II**, which was ultimately aromatized to afford the target product **3a**.

## 3. Materials and Methods

### 3.1. General Information

2-(1*H*-pyrrol-1-yl)anilines/2-(1*H*-indolo-1-yl)anilines and 2-aminobenzamides were obtained based on procedures reported in the literature [46,47]. All other reagents were market available and used with no further purification. We monitored the reactions using thin layer chromatography (TLC). Reactions requiring heat were performed in an oil bath. ^1^H NMR (400 MHz or 500 MHz) and ^13^C NMR (100 MHz or 125 MHz) spectra were recorded on a Bruker spectrometer, with CDCl_3_ or DMSO-*d_6_* as a solvent and tetramethylsilane (TMS) as an internal standard (Supplementary Materials). HRMS spectra (ESI) were acquired on a Bruker impact II spectrograph in positive-ion mode with an ESI ion source.

### 3.2. General Experimental Procedures for 2-(1H-Pyrrol-1-yl) Anilines (***1***)

The substituted 2-nitroaniline (2.7626 g, 20 mmol, 1 equiv.) and 2, 5-dimethoxytetrahydrofuran (2.9075 g, 22 mmol, 1.1 equiv.) were mixed in HOAc (30 mL) and stirred vigorously under reflux conditions for 2−3 h. To neutralize the reaction mixture, Na_2_CO_3_ aqueous solution was added and extracted three times with EtOAc. The organic layer was then dried with anhydrous Na_2_SO_4_ and the residue was obtained after vacuum evaporation. Iron powder (4.4680 g, 80 mmol, 5 equiv.) and NH_4_Cl (1.0698 g, 20 mmol, 1 equiv.) were added to the residue in water (40 mL) and refluxed for 4–9 h. When the reaction was complete, the mixture was extracted three times with EtOAc. The organic layer was then dried with anhydrous Na_2_SO_4_, and the residue was obtained after vacuum evaporation. The residue was subsequently purified by silica gel chromatography to yield the desirable compounds.

### 3.3. General Experimental Procedures for 2-(1H-Indolo-1-yl)anilines (***1***)

The substituted fluoro-2-nitrobenzene (2.8220 g, 20 mmol, 1 equiv.), indole (2.3430 g, 20 mmol, 1 equiv.) and NaOH (0.8000 g, 20 mmol, 1 equiv.) were mixed in DMSO (30 mL) and stirred at room temperature for 4 h. The reaction mixture was extracted with EtOAc (3 × 30 mL). The organic layer was then dried with anhydrous Na_2_SO_4_ and a solid was obtained after vacuum evaporation. Iron powder (5.6850 g, 100 mmol, 5 equiv.) and NH_4_Cl (1.0700 g, 20 mmol, 1 equiv.) were added to the solvent and refluxed for 5−9 h. After cooling, the reaction mixture was extracted three times with EtOAc and brine solution and subsequently dried with anhydrous Na_2_SO_4_. After filtration, a vacuum was used to remove the solvent. The resulting solid was then purified by silica gel column chromatography to obtain the desired compound.

### 3.4. General Experimental Procedures for Compounds ***3a***–***3t*** and ***5a***–***5m***

Various substituted amines (0.5 mmol, 1 equiv.), epoxides (1.0 mmol, 2 equiv.), iodine (0.1269 g, 0.5 mmol, 1 equiv.) and TsOH (0.0861 g, 0.5 mmol, 1 equiv.) were added to CH_3_CN (1mL) in a sealed tube. The reaction mixture was stirred vigorously at 120 °C for 4 h. After completion of the reaction, monitored by TLC, the reaction mixture was neutralized with Na_2_S_2_O_3_ and extracted three times with EtOAc and H_2_O, followed by drying with anhydrous Na_2_SO_4_. The product was purified by silica gel column chromatography to give the desired compounds **3a**–**3t** and **5a**–**5m**.

### 3.5. Characterization Data

*4-benzylpyrrolo[1,2-a]quinoxaline* (**3a**), purification on a silica gel (petroleum ether/ethyl acetate = 15:1) afforded compound **3a** as a light yellow solid (98.2 mg, 76% yield); ^1^H NMR (400 MHz, CDCl_3_): *δ* 7.86–7.84 (m, 1 H), 7.65 (dd, *J* = 2.9, 1.4 Hz, 1 H), 7.60–7.57 (m, 1 H), 7.31–7.24 (m, 4 H), 7.14 (t, *J* = 8.0 Hz, 2 H), 7.10–7.03 (m, 1 H), 6.69 (dd, *J* = 4.0, 1.2 Hz, 1 H), 6.62–6.62 (m, 1 H), 4,23 (s, 2 H). ^13^C NMR (100 MHz, CDCl_3_): *δ* 154.1, 136.9, 134.7, 128.5, 127.8, 127.8, 127.4, 126.1, 125.5, 124.7, 124.0, 113.2, 112.6, 112.5, 106.1, 41.5. HRMS (ESI-TOF): *m*/*z* [M + H]^+^ calcd for C_18_H_15_N_2_^+^: 259.1229; found: 259.1233.
*4-benzyl-8-methylpyrrolo[1,2-a]quinoxaline* (**3b**), purification on a silica gel (petroleum ether/ethyl acetate = 15:1) afforded compound **3b** as a yellow solid (84.4 mg, 62% yield); ^1^H NMR (400 MHz, CDCl_3_): *δ* 7.77 (d, *J* = 8.2 Hz, 1 H), 7.72 (dd, *J* = 2.7, 1.3 Hz, 1 H), 7.50 (s, 1 H), 7.32 (d, *J* = 7.4 Hz, 2 H), 7.20–7.10 (m, 4 H), 6.71 (dd, *J* = 4.0, 1.3 Hz, 1 H), 6.67 (dd, *J* = 4.0, 2.7 Hz, 1 H), 4.26 (s, 2 H), 2.43 (s, 3 H). ^13^C NMR (100 MHz, CDCl_3_): *δ* 153.2, 137.1, 136.6, 132.8, 128.3, 127.8, 127.4, 126.0, 125.5, 125.3, 124.9, 112.8, 112.6, 112.5, 105.7, 41.5, 20.7. HRMS (ESI-TOF): *m*/*z* [M + H]^+^ calcd for C_19_H_17_N_2_^+^: 273.1385; found: 273.1381.
*4-benzyl-8-methoxypyrrolo[1,2-a]quinoxaline* (**3c**), purification on a silica gel (petroleum ether/ethyl acetate = 8:1) afforded compound **3c** as a yellow solid (85.1 mg, 59% yield); ^1^H NMR (400 MHz, CDCl_3_): *δ* 7.77 (d, *J* = 8.9 Hz, 1 H), 7.57 (dd, *J* = 2.8, 1.3 Hz, 1 H), 7.29 (d, *J* = 6.8 Hz, 2 H), 7.14 (t, *J* = 7.4 Hz, 2 H), 7.08–7.03 (m, 2 H), 6.88 (dd, *J* = 8.9, 2.7 Hz, 1 H), 6.66–6.61(m, 2 H), 4.21 (s, 2 H), 3.75 (s, 3 H). ^13^C NMR (100 MHz, CDCl_3_): *δ* 157.7, 151.5, 137.2, 129.8, 129.2, 127.8, 127.4, 126.9, 125.4, 124.7, 112.7, 112.6, 111.5, 105.5, 96.4, 54.6, 41.4. HRMS (ESI-TOF): *m*/*z* [M + H]^+^ calcd for C_19_H_17_ON_2_^+^: 289.1335; found: 289.1339.
*4-benzyl-8-fluoropyrrolo[1,2-a]quinoxaline* (**3d**), purification on a silica gel (petroleum ether/ethyl acetate = 15:1) afforded compound **3d** as a yellow solid (102.3 mg, 74% yield); ^1^H NMR (400 MHz, CDCl_3_): *δ* 7.63 (dd, *J* = 2.8, 1.3 Hz, 1 H), 7.56–7.50 (m, 2 H), 7.31 (d, *J* = 7.2 Hz, 2 H), 7.16 (t, *J* = 7.5 Hz, 2 H), 7.11–7.00 (m, 2 H), 6.74 (d, *J* = 4.0 Hz, 1 H), 6.64 (dd, *J* = 4.1, 2.7 Hz, 1 H), 4.22 (s, 2 H). ^13^C NMR (100 MHz, CDCl_3_): *δ* 158.7 (d, *J*_C-F_ = 243.4 Hz), 155.4, 136.7, 136.0 (d, *J*_C-F_ = 11.4 Hz), 127.65 (d, *J*_C-F_ = 34.6 Hz), 125.6, 124.5, 122.86 (d, *J*_C-F_ = 2.3 Hz), 113.9 (d, *J*_C-F_ = 22.2 Hz), 113.7 (d, *J*_C-F_ = 21.1 Hz), 113.6, 113.5, 113.4, 112.7, 106.4, 41.4. ^19^F NMR (377 MHz, CDCl_3_): *δ* −116.7 (s). HRMS (ESI-TOF): *m*/*z* [M + H]^+^ calcd for C_18_H_14_FN_2_^+^: 277.1135; found: 277.1132.
*4-benzyl-7-methylpyrrolo[1,2-a]quinoxaline* (**3e**), purification on a silica gel (petroleum ether/ethyl acetate = 15:1) afforded compound **3e** as a yellow solid (84.4 mg, 62% yield); ^1^H NMR (400 MHz, CDCl_3_): *δ* 7.67 (d, *J* = 1.5 Hz, 2 H), 7.52 (d, *J* = 8.3 Hz, 1 H), 7.31 (d, *J* = 7.2 Hz, 2 H), 7.18–7.12 (m, 3 H), 7.08 (t, *J* = 7.3 Hz, 1 H), 6.70 (dd, *J* = 4.0, 1.3 Hz, 1 H), 6.63 (dd, *J* = 4.0, 2.7 Hz, 1 H), 4.24 (s, 2 H), 2.36 (s, 3 H). ^13^C NMR (100 MHz, CDCl_3_): *δ* 154.1, 137.0, 134.8, 133.8, 128.5, 127.8, 127.4, 127.2, 125.5, 124.7, 124.1, 112.9, 112.3, 112.2, 105.7, 41.5, 20.0. HRMS (ESI-TOF): *m*/*z* [M + H]^+^ calcd for C_19_H_17_N_2_^+^: 273.1385; found: 273.1384.
*4-benzyl-7-(tert-butyl)pyrrolo[1,2-a]quinoxaline* (**3f**), purification on a silica gel (petroleum ether/ethyl acetate = 20:1) afforded compound **3f** as a yellow solid (114.8 mg, 73% yield); ^1^H NMR (400 MHz, CDCl_3_): *δ* 7.90 (s, 1 H), 7.68 (dd, *J* = 2.8, 1.3 Hz, 1 H), 7.59 (d, *J* = 8.6 Hz, 1 H), 7.40 (dd, *J* = 8.6, 2.3 Hz, 1 H), 7.30 (d, *J* = 6.8 Hz, 2 H), 7.14 (t, *J* = 7.5 Hz, 2 H), 7.08–7.04 (m, 1 H), 6.69 (d, *J* = 4.0 Hz, 1 H), 6.63 (dd, *J* = 4.0, 2.7 Hz, 1 H), 4.25 (s, 2 H), 1.30 (s, 9 H). ^13^C NMR (100 MHz, CDCl_3_): *δ* 154.0, 147.3, 137.0, 134.5, 127.7, 127.4, 125.5, 125.0, 124.7, 124.0, 123.8, 112.9, 112.4, 112.1, 105.8, 41.6, 33.7, 30.4. HRMS (ESI-TOF): *m*/*z* [M + H]^+^ calcd for C_22_H_23_N_2_^+^: 315.1855; found: 315.1859.
*4-benzyl-7-fluoropyrrolo[1,2-a]quinoxaline* (**3g**), purification on a silica gel (petroleum ether/ethyl acetate = 20:1) afforded compound **3g** as a yellow solid (98.1 mg, 71% yield); ^1^H NMR (400 MHz, CDCl_3_): *δ* 7.67 (dd, *J* = 2.8, 1.3 Hz, 1 H), 7.59 (dd, *J* = 9.0, 5.0 Hz, 1 H), 7.53 (dd, *J* = 9.5, 2.9 Hz, 1 H), 7.32 (d, *J* = 7.1 Hz, 2 H), 7.18 (t, *J* = 7.4 Hz, 2 H), 7.12–7.04 (m, 2 H), 6.76 (dd, *J* = 4.0, 1.3 Hz, 1 H), 6.66 (dd, *J* = 4.1, 2.7 Hz, 1 H), 4.23 (s, 2 H). ^13^C NMR (100 MHz, CDCl_3_): *δ* 158.7 (d, *J*_C-F_= 243.5 Hz), 155.4, 136.7, 136.1 (d, *J*_C-F_ = 11.6 Hz), 127.8, 127.5, 125.6, 124.5, 122.9 (d, *J*_C-F_= 2.2 Hz), 114.0 (d, *J*_C-F_ = 22.0 Hz), 113.8 (d, *J*_C-F_ = 24.0 Hz), 113.6 (d, *J*_C-F_= 9.0 Hz), 113.4, 112.7, 106.4, 41.4. ^19^F NMR (377 MHz, CDCl_3_): *δ* −116.8 (s). HRMS (ESI-TOF): *m*/*z* [M + H]^+^ calcd for C_18_H_14_FN_2_^+^: 277.1135; found: 277.1332.
*4-benzyl-7-chloropyrrolo[1,2-a]quinoxaline* (**3h**), purification on a silica gel (petroleum ether/ethyl acetate = 20:1) afforded compound **3h** as a yellow solid (92.2 mg, 63% yield); ^1^H NMR (400 MHz, CDCl_3_): *δ* 7.88 (d, *J* = 2.4 Hz, 1 H), 7.74 (d, *J* = 1.7 Hz, 1 H), 7.63 (d, *J* = 8.7 Hz, 1 H), 7.33 (d, *J* = 8.6 Hz, 3 H), 7.22–7.18 (m, 2 H), 7.14–7.11 (m, 1 H), 6.81 (d, *J* = 4.5 Hz, 1 H), 6.73–6.72 (m, 1 H), 4.27 (s, 2 H). ^13^C NMR (100 MHz, CDCl_3_): *δ* 155.4, 136.6, 135.7, 129.1, 128.0, 127.8, 127.5, 126.0, 125.6, 124.8, 124.6, 113.6, 113.5, 112.9, 106.5, 41.4. HRMS (ESI-TOF): *m*/*z* [M + H]^+^ calcd for C_18_H_14_ClN_2_^+^: 293.0839; found: 293.0837.
*4-benzyl-7-bromopyrrolo[1,2-a]quinoxaline* (**3i**), purification on a silica gel (petroleum ether/ethyl acetate = 20:1) afforded compound **3i** as a yellow solid (69.1 mg, 41% yield); ^1^H NMR (400 MHz, CDCl_3_): *δ* 7.98 (d, *J* = 2.3 Hz, 1 H), 7.63 (d, J = 1.5 Hz, 1 H), 7.44 (d, J = 8.8 Hz, 1 H), 7.37 (dd, J = 8.8, 2.3 Hz, 1 H), 7.31 (d, *J* = 7.6 Hz, 2 H), 7.18 (t, *J* = 7.5 Hz, 2 H), 7.10 (t, *J* = 7.3 Hz, 1 H), 6.75 (d, *J* = 4.1 Hz, 1 H), 6.67–6.65 (m, 1 H), 4.23 (s, 2 H). ^13^C NMR (100 MHz, CDCl_3_): *δ* 155.3, 136.6, 136.0, 131.1, 128.8, 127.8, 127.5, 125.7, 125.2, 124.6, 116.5, 113.9, 113.5, 113.0, 106.6, 41.4. HRMS (ESI-TOF): *m*/*z* [M + H]^+^ calcd for C_18_H_14_BrN_2_^+^: 337.0334; found: 337.0333.
*4-benzyl-7-iodopyrrolo[1,2-a]quinoxaline* (**3j**), purification on a silica gel (petroleum ether/ethyl acetate = 20:1) afforded compound **3i** as a yellow solid (76.8 mg, 40% yield); ^1^H NMR (400 MHz, CDCl_3_): *δ* 8.22 (d, *J* = 2.0 Hz, 1 H), 7.69 (dd, *J* = 2.8, 1.4 Hz, 1 H), 7.59 (dd, *J* = 8.6, 2.0 Hz, 1 H), 7.38 (d, *J* = 8.6 Hz, 1 H), 7.32 (d, *J* = 7.5 Hz, 2 H), 7.22–7.15 (m, 2 H), 7.11 (t, *J* = 7.3 Hz, 1 H), 6.78 (dd, *J* = 4.0, 1.4 Hz, 1 H), 6.70 (dd, *J* = 4.1, 2.7 Hz, 1 H), 4.24 (s, 2 H). ^13^C NMR (100 MHz, CDCl_3_): *δ* 155.2, 137.3, 136.6, 136.2, 134.5, 127.8, 127.5, 125.9, 125.7, 124.6, 114.2, 113.5, 113.0, 106.7, 87.1, 41.4. HRMS (ESI-TOF): *m*/*z* [M + H]^+^ calcd for C_18_H_14_IN_2_^+^: 385.0195; found: 385.0196.
*4-benzyl-9-methylpyrrolo[1,2-a]quinoxaline* (**3k**), purification on a silica gel (petroleum ether/ethyl acetate = 15:1) afforded compound **3k** as a yellow solid (87.1 mg, 64% yield); ^1^H NMR (400 MHz, CDCl_3_): *δ* 8.03 (dd, *J* = 2.9, 1.4 Hz, 1 H), 7.72 (dd, *J* = 7.9, 1.8 Hz, 1 H), 7.28 (d, *J* = 6.8 Hz, 2 H), 7.16–7.10 (m, 3 H), 7.07–7.02 (m, 2 H), 6.72 (dd, *J* = 4.1, 1.3 Hz, 1 H), 6.59 (dd, *J* = 4.2, 2.8 Hz, 1 H), 4.21 (s, 2 H), 2.68 (s, 3 H). ^13^C NMR (100 MHz, CDCl_3_): *δ* 153.7, 137.0, 136.4, 129.6, 127.7, 127.4, 127.1, 126.5, 126.0, 125.4, 124.2, 123.4, 118.8, 111.8, 105.4, 41.3, 22.7. HRMS (ESI-TOF): *m*/*z* [M + H]^+^ calcd for C_19_H_17_N_2_^+^: 273.1385; found: 273.1390.
*4-Benzyl-9-chloropyrrolo[1,2-a]quinoxaline* (**3l**). Purification on a silica gel (petroleum ether/ethyl acetate = 15:1) afforded compound **3l** as a yellow solid (95.2 mg, 65% yield); ^1^H NMR (400 MHz, CDCl_3_): *δ* 8.90 (dd, *J* = 2.9, 1.3 Hz, 1 H), 7.72 (dd, *J* = 8.0, 1.5 Hz, 1 H), 7.27 (dd, *J* = 8.0, 1.5 Hz, 3 H), 7.15–7.09 (m, 3 H), 7.07–7.03 (m, 1 H), 6.76 (dd, *J* = 4.2, 1.3 Hz, 1 H), 6.60 (dd, *J* = 4.2, 2.9 Hz, 1 H), 4.18(s, 2 H). ^13^C NMR (100 MHz, CDCl_3_): *δ* 154.6, 137.7, 136.7, 128.5, 127.8, 127.7, 127.4, 125.8, 125.6, 124.4, 123.5, 119.8, 119.8, 112.0, 106.4, 41.2. HRMS (ESI-TOF): *m*/*z* [M + H]^+^ calcd for C_18_H_14_ClN_2_^+^: 293.0839; found: 293.0844.
*4-benzyl-7,8-dimethylpyrrolo[1,2-a]quinoxaline* (**3m**), purification on a silica gel (petroleum ether/ethyl acetate = 10:1) afforded compound **3m** as a yellow solid (91.6 mg, 64% yield); ^1^H NMR (400 MHz, CDCl_3_): *δ* 7.64 (s, 1 H), 7.62 (s, 1 H), 7.41 (d, *J* = 5.3 Hz, 1 H), 7.30 (d, *J* = 8.1 Hz, 2 H), 7.15 (t, *J* = 7.4 Hz, 2 H), 7.07 (t, *J* = 7.3 Hz, 1 H), 6.67 (dd, *J* = 4.0, 1.4 Hz, 1 H), 6.62–6.60 (m, 1 H), 4.23 (s, 2 H), 2.29 (s, 3 H), 2.25 (s, 3 H). ^13^C NMR (100 MHz, CDCl_3_): *δ* 153.1, 137.2, 135.5, 133.0, 132.8, 128.8, 127.8, 127.4, 125.4, 124.8, 124.2, 113.0, 112.6, 112.2, 105.4, 41.5, 19.1, 18.5. HRMS (ESI-TOF): *m*/*z* [M + H]^+^ calcd for C_20_H_19_N_2_^+^: 287.1542; found: 287.1540.
*4-benzyl-7,8-difluoropyrrolo[1,2-a]quinoxaline* (**3n**), purification on a silica gel (petroleum ether/ethyl acetate = 15:1) afforded compound **3n** as a yellow solid (108.8 mg, 76% yield); ^1^H NMR (400 MHz, CDCl_3_): *δ* 7.68 (dd, *J* = 11.0, 8.1 Hz, 1 H), 7.62 (dd, *J* = 2.7, 1.3 Hz, 1 H), 7.48 (dd, *J* = 10.5, 7.3 Hz, 1 H), 7.32 (d, *J* = 6.9 Hz, 2 H), 7.22–7.17 (m, 2 H), 7.14–7.11 (m, 1 H), 6.80 (dd, J = 4.1, 1.3 Hz, 1 H), 6.73 (dd, *J* = 4.0, 2.8 Hz, 1 H), 4.25 (s, 2 H). ^13^C NMR (100 MHz, CDCl_3_): *δ* 154.8 (d, *J*_C-F_ = 2.9 Hz), 148.3 (dd, *J*_C-F_ = 248.7, 14.4 Hz), 146.9 (dd, *J*_C-F_ = 245.2, 13.7 Hz), 136.6, 129.9, 127.8, 127.5, 125.7, 124.4, 122.6 (d, *J*_C-F_ = 8.7 Hz), 116.2 (d, *J*_C-F_ = 19.6 Hz), 113.5, 113.2, 106.6, 101.1 (d, *J*_C-F_ = 22.2 Hz), 41.3. ^19^F NMR (377 MHz, CDCl_3_): *δ* -135.1 (s), -140.2 (s). HRMS (ESI-TOF): *m*/*z* [M + H]^+^ calcd for C_18_H_13_F_2_N_2_^+^: 295.1041; found: 295.1039.
*4-benzyl-7,8-dichloropyrrolo[1,2-a]quinoxaline* (**3o**), purification on a silica gel (petroleum ether/ethyl acetate = 25:1) afforded compound **3o** as a yellow solid (106.3 mg, 65% yield); ^1^H NMR (400 MHz, CDCl_3_): *δ* 7.94 (s, 1 H), 7.75 (s, 1 H), 7.64 (dd, *J* = 2.8, 1.3 Hz, 1 H), 7.31 (d, *J* = 6.9 Hz, 2 H), 7.22–7.11 (m, 3 H), 6.80 (dd, *J* = 4.0, 1.3 Hz, 1 H), 6.71 (dd, *J* = 4.0, 2.8 Hz, 1 H), 4.23 (s, 2 H). ^13^C NMR (100 MHz, CDCl_3_): *δ* 155.6, 136.4, 134.3, 129.6, 129.6, 127.8, 127.6, 127.5, 125.7, 125.4, 124.5, 114.1, 113.8, 113.4, 107.1, 41.4. HRMS (ESI-TOF): *m*/*z* [M + H]^+^ calcd for C_18_H_13_Cl_2_N_2_^+^: 327.0450; found: 327.0455.
*6-benzylindolo[1,2-a]quinoxaline* (**3p**), purification on a silica gel (petroleum ether/ethyl acetate = 25:1) afforded compound **3p** as a yellow solid (74.1 mg, 48% yield); ^1^H NMR (400 MHz, CDCl_3_): *δ* 8.21 (dd, *J* = 15.8, 8.6 Hz, 2 H), 7.89 (dd, *J* = 7.9, 1.8 Hz, 1 H), 7.71 (d, *J* = 8.0 Hz, 1 H), 7.42–7.23 (m, 6 H), 7.17 (t, *J* = 7.8 Hz, 2 H), 7.09 (t, *J* = 8.1 Hz, 1 H), 6.95 (s, 1 H), 4.30 (s, 2 H). ^13^C NMR (100 MHz, CDCl_3_): *δ* 155.6, 136.6, 134.7, 131.7, 129.2, 128.9, 128.1, 127.9, 127.7, 127.5, 127.0, 125.6, 123.1, 122.9, 121.6, 121.4, 113.5, 113.4, 99.5, 41.6. HRMS (ESI-TOF): *m*/*z* [M + H]^+^ calcd for C_22_H_17_N_2_^+^: 309.1385; found: 309.1382.
*6-qenzyl-3-methylindolo[1,2-a]quinoxaline* (**3q**), purification on a silica gel (petroleum ether/ethyl acetate = 20:1) afforded compound **3q** as a yellow solid (61.3 mg, 38% yield); ^1^H NMR (400 MHz, CDCl_3_): *δ* 8.31 (d, *J* = 8.6 Hz, 1 H), 8.14 (s, 1 H), 7.79 (dd, *J* = 16.9, 8.0 Hz, 2 H), 7.42–7.27 (m, 3 H), 7.29 (t, *J* = 7.4 Hz, 1 H), 7.23–7.06 (m, 4 H), 6.98 (s, 1 H), 4.33(s, 2 H), 2.51(s, 3 H). ^13^C NMR (100 MHz, CDCl_3_): *δ* 154.6, 137.5, 136.7, 132.7, 131.7, 129.1, 128.7, 128.3, 128.0, 127.7, 127.5, 125.6, 124.0, 122.9, 121.6, 121.5, 113.9, 113.6, 99.4, 41.6, 21.1. HRMS (ESI-TOF): *m*/*z* [M + H]^+^ calcd for C_23_H_19_N_2_^+^: 323.1542; found: 323.1546.
*6-Benzyl-7-methylindolo[1,2-a]quinoxaline* (**3r**). Purification on a silica gel (petroleum ether/ethyl acetate = 15:1) afforded compound **3r** as a yellow solid (56.4 mg, 35% yield); ^1^H NMR (400 MHz, CDCl_3_): *δ* 8.44 (t, *J* = 7.9 Hz, 2 H), 7.99 (s, 1 H), 7.88 (d, *J* = 8.1 Hz, 1 H), 7.56 (q, *J* = 7.1 Hz, 2 H), 7.41 (dt, *J* = 14.1, 7.1 Hz, 2 H), 7.27–7.25 (m, 4 H), 7.20 (q, *J* = 4.3 Hz, 1 H), 4.64 (s, 2 H), 2.63 (s, 3 H). ^13^C NMR (100 MHz, CDCl_3_): *δ* 156.8, 137.9, 132.1, 130.6, 130.0, 129.5, 128.9, 128.6, 128.5, 128.4, 128.2, 126.5, 124.8, 124.0, 123.8, 122.0, 120.7, 114.5, 114.4, 29.7, 11.0. HRMS (ESI-TOF): *m*/*z* [M + H]^+^ calcd for C_23_H_19_N_2_^+^: 323.1542; found: 323.1543.
*4-propylpyrrolo[1,2-a]quinoxaline* (**3s**), purification on a silica gel (petroleum ether/ethyl acetate = 10:1) afforded compound **3s** as a yellow solid (42.1 mg, 40% yield); ^1^H NMR (500 MHz, CDCl_3_): *δ* 7.84 (dd, *J* = 7.9, 1.5 Hz, 1 H), 7.81–7.79 (m, 1 H), 7.73 (d, *J* = 8.0 Hz, 1 H), 7.35 (dtd, *J* = 21.0, 7.4, 1.4 Hz, 2 H), 6.82 (dd, *J* = 3.9, 1.2 Hz, 1 H), 6.75 (dd, *J* = 3.8, 2.8 Hz, 1 H), 2.91 (dd, *J* = 8.5, 7.1 Hz, 2 H), 1.86 (dq, *J* = 15.0, 7.4 Hz, 2 H), 1.00 (t, *J* = 7.4 Hz, 3 H). ^13^C NMR (125 MHz, CDCl_3_): *δ* 156.4, 135.0, 128.4, 126.2, 125.8, 125.1, 124.0, 113.0, 112.6, 112.4, 105.2, 36.8, 20.9, 13.3. HRMS (ESI-TOF): *m*/*z* [M + H]^+^ calcd for C_14_H_15_N_2_^+^: 211.1229; found: 211.1233.
*(E)-4-(prop-1-en-1-yl)pyrrolo[1,2-a]quinoxaline* (**3t**), purification on a silica gel (petroleum ether/ethyl acetate = 10:1) afforded compound **3t** as a yellow solid (52.1 mg, 40% yield); ^1^H NMR (500 MHz, CDCl_3_): *δ* 7.90–7.82 (m, 2 H), 7.77–7.72 (dd, *J* = 8.0, 1.0 Hz, 1 H), 7.42–7.29 (m, 2 H), 7.18–7.09 (qd, *J* = 13.5, 7.0 Hz, 1 H), 6.93–6.90 (dd, *J* = 4.0, 1.0 Hz, 1 H), 6.80 (dd, *J* = 4.0, 1.5 Hz, 1 H), 6.78 (dd, *J* = 7.0, 2.5 Hz, 1 H), 1.98 (dd, *J* = 6.8, 1.7 Hz, 3 H). ^13^C NMR (125 MHz, CDCl_3_): *δ* 149.2, 135.1, 134.4, 128.6, 126.2, 125.9, 125.7, 124.6, 124.2, 113.3, 112.6, 112.5, 105.0, 17.8. HRMS (ESI-TOF): *m*/*z* [M + H]^+^ calcd for C_14_H_14_N_2_^+^: 209.1072; found: 209.1071.
*2-benzylquinazolin-4(3H)-one* (**5a**), purification on a silica gel (petroleum ether/ethyl acetate = 2:1) afforded compound **5a** as a white solid (82.7 mg, 70% yield); ^1^H NMR (400 MHz, DMSO-*d_6_*): *δ* 12.43 (s, 1 H), 8.09 (dd, *J* = 7.9, 1.6 Hz, 1 H), 7.78 (ddd, *J* = 8.4, 7.0, 1.6 Hz, 1 H), 7.62 (d, *J* = 8.5 Hz, 1 H), 7.50–7.44 (m, 1 H), 7.39 (d, *J* = 7.9 Hz, 2 H), 7.33 (t, *J* = 7.6 Hz, 2 H), 7.28–7.22 (m, 1 H), 3.95 (s, 2 H). ^13^C NMR (100 MHz, DMSO-*d_6_*): *δ* 167.1, 161.2, 154.1, 141.8, 139.6, 134.1, 133.7, 132.2, 132.0, 131.4, 130.9, 126.0, 46.0. HRMS (ESI-TOF): *m*/*z* [M + H]^+^ calcd for C_15_H_13_ON_2_^+^: 237.1002; found: 237.1005.
*2-benzyl-6-methylquinazolin-4(3H)-one* (**5b**), purification on a silica gel (petroleum ether/ethyl acetate = 5:1) afforded compound **5b** as a white solid (92.6 mg, 74% yield). ^1^H NMR (400 MHz, DMSO-*d_6_*): *δ* 12.32 (s, 1 H), 7.87 (s, 1 H), 7.59 (dd, *J* = 8.3, 2.2 Hz, 1 H), 7.51 (d, *J* = 8.2 Hz, 1 H), 7.38 (d, *J* = 6.8 Hz, 2 H), 7.32 (t, *J* = 7.5 Hz, 2 H), 7.24 (t, *J* = 7.2 Hz, 1 H), 3.92 (s, 2 H), 2.42 (s, 3H). ^13^C NMR (100 MHz, DMSO-*d_6_*): *δ* 162.3, 155.5, 147.4, 137.1, 136.3, 136.1, 129.3, 129.0, 127.3, 127.2, 125.5, 121.0, 41.2, 21.2. HRMS (ESI-TOF): *m*/*z* [M + H]^+^ calcd for C_16_H_15_ON_2_^+^: 251.1178; found: 251.1177.
*2-benzyl-6-methoxyquinazolin-4(3H)-one* (**5c**), purification on a silica gel (petroleum ether/ethyl acetate = 1:1) afforded compound **5c** as a white solid (77.3 mg, 58% yield); ^1^H NMR (400 MHz, DMSO-*d_6_*): *δ* 12.38 (s, 1 H), 7.57 (d, *J* = 8.8 Hz, 1 H), 7.48 (d, *J* = 2.9 Hz, 1 H), 7.41–7.36 (m, 3 H), 7.32 (d, *J* = 15.0 Hz, 2 H), 7.24 (t, *J* = 7.2 Hz, 1 H), 3.92 (s, 2 H), 3.85 (s, 3 H). ^13^C NMR (100 MHz, DMSO-*d_6_*): *δ* 162.2, 157.8, 154.1, 143.9, 137.2, 129.3, 129.1, 129.0, 127.2, 124.3, 122.0, 106.2, 56.0, 41.1. HRMS (ESI-TOF): *m*/*z* [M + H]^+^ calcd for C_15_H_15_ON_2_+: 267.1127; found: 267.1123.
*2-benzyl-6-fluoroquinazolin-4(3H)-one* (**5d**), purification on a silica gel (petroleum ether/ethyl acetate = 5:1) afforded compound **5d** as a white solid (90.3 mg, 71% yield); ^1^H NMR (400 MHz, DMSO-*d_6_*): *δ* 12.60 (s, 1 H), 7.76 (dd, *J* = 8.6, 2.7 Hz, 1 H), 7.71–7.65 (m, 2 H), 7.39 (d, *J* = 6.9 Hz, 2 H), 7.33 (t, *J* = 7.6 Hz, 2 H), 7.25 (t, *J* = 7.3 Hz, 1 H), 3.95 (s, 2 H). ^13^C NMR (100 MHz, DMSO-d_6_): *δ* 161.8 (d, *J*_C-F_ = 3.3 Hz), 160.2 (d, *J*_C-F_ = 244.9 Hz), 156.0 (d, *J*_C-F_ = 2.2 Hz), 146.2 (d, *J*_C-F_ = 1.8 Hz), 136.9, 130.2 (d, *J*_C-F_ = 8.0 Hz), 129.4, 129.0, 127.3, 123.3 (d, J_C-F_ = 24.0 Hz), 122.4 (d, J_C-F_ = 8.4 Hz), 110.8 (d, *J*_C-F_ = 23.3 Hz), 41.1. ^19^F NMR (377 MHz, DMSO-*d_6_*): *δ* −114.1 (s). HRMS (ESI-TOF): *m*/*z* [M + H]^+^ calcd for C_15_H_12_OFN_2_^+^: 255.0927; found: 255.0928.
*2-benzyl-6-chloroquinazolin-4(3H)-one* (**5e**), purification on a silica gel (petroleum ether/ethyl acetate = 5:1) afforded compound **5e** as a white solid (101.55 mg, 75% yield); ^1^H NMR (400 MHz, DMSO-*d_6_*): *δ* 12.61 (s, 1 H), 8.01 (d, *J* = 2.5 Hz, 1 H), 7.80 (dd, *J* = 8.8, 2.6 Hz, 1 H), 7.64 (d, *J* = 8.7 Hz, 1 H), 7.38 (d, *J* = 6.8 Hz, 2 H), 7.33 (t, *J* = 7.5 Hz, 2 H), 7.25 (t, *J* = 7.2 Hz, 1 H), 3.95 (s, 2 H). ^13^C NMR (100 MHz, DMSO-*d_6_*): *δ* 161.4, 157.1, 148.1, 136.8, 135.0, 130.9, 129.7, 129.4, 129.0, 127.3, 125.2, 122.5, 41.2. HRMS (ESI-TOF): *m*/*z* [M + H]^+^ calcd for C_15_H_12_OClN_2_^+^:271.0632; found: 271.0635.
*2-benzyl-6-bromoquinazolin-4(3H)-one* (**5f**), purification on a silica gel (petroleum ether/ethyl acetate = 5:1) afforded compound **5f** as a white solid (93.0 mg, 59% yield); ^1^H NMR (400 MHz, DMSO-*d_6_*): *δ* 12.65 (s, 1 H), 8.15 (d, *J* = 2.4 Hz, 1 H), 7.92 (dd, *J* = 8.7, 2.4 Hz, 1 H), 7.57 (d, *J* = 8.8 Hz, 1 H), 7.38 (d, *J* = 6.9 Hz, 2 H), 7.33 (t, *J* = 7.5 Hz, 2 H), 7.25 (t, *J* = 7.2 Hz, 1 H), 3.94 (s, 2 H). ^13^C NMR (100 MHz, DMSO-*d_6_*): *δ* 161.2, 157.2, 148.4, 137.7, 136.8, 129.8, 129.4, 129.0, 128.3, 127.3, 122.9, 119.0, 41.2. HRMS (ESI-TOF): *m*/*z* [M + H]^+^ calcd for C_15_H_12_OBrN_2_^+^: 315.0127; found: 315.0124.
*2-benzyl-7-methylquinazolin-4(3H)-one* (**5g**), purification on a silica gel (petroleum ether/ethyl acetate = 2:1) afforded compound **5g** as a white solid (75.2 mg, 60% yield); ^1^H NMR (400 MHz, DMSO-*d_6_*): *δ* 12.31 (s, 1 H), 7.97 (d, *J* = 8.1 Hz, 1 H), 7.47–7.37 (m, 3 H), 7.35–7.20 (m, 4 H), 3.93 (s, 2 H), 2.43 (s, 3 H). ^13^C NMR (100 MHz, DMSO-*d_6_*): *δ* 162.2, 156.5, 149.5, 145.3, 137.1, 129.3, 128.9, 128.0, 127.2, 127.1, 126.0, 118.8, 41.2, 21.8. HRMS (ESI-TOF): *m*/*z* [M + H]^+^ calcd for C_16_H_15_ON_2_^+^: 251.1178; found: 251.1175.
*2-benzyl-7-fluoroquinazolin-4(3H)-one* (**5h**), purification on a silica gel (petroleum ether/ethyl acetate = 2:1) afforded compound **5h** as a white solid (85.2 mg, 67% yield); ^1^H NMR (400 MHz, DMSO-*d_6_*): *δ* 12.54 (s, 1 H), 8.15 (dd, *J* = 8.9, 6.4 Hz, 1 H), 7.42–7.37 (m, 3 H), 7.36–7.30 (m, 3 H), 7.25 (t, *J* = 7.3 Hz, 1 H), 3.95 (s, 2 H). ^13^C NMR (100 MHz, DMSO-*d_6_*): δ = 166.2 (d, *J* _C-F_ = 250.7 Hz), 161.6, 158.0, 151.6 (d, *J* _C-F_ = 13.2 Hz), 136.8, 129.4, 129.2, 129.0, 127.3, 118.3 (d, *J*_C-F_ = 1.8 Hz), 115.2 (d, *J*_C-F_ = 23.6 Hz), 112.5 (d, *J*_C-F_ = 21.8 Hz), 41.2. ^19^F NMR (377 MHz, DMSO-*d_6_*): *δ* −104.5 (s). HRMS (ESI-TOF): *m*/*z* [M + H]^+^ calcd for C_15_H_12_OFN_2_^+^: 255.0927; found: 255.0930.
*2-benzyl-7-chloroquinazolin-4(3H)-one* (**5i**), purification on a silica gel (petroleum ether/ethyl acetate = 2:1) afforded compound **5i** as a white solid (88.1 mg, 65% yield); ^1^H NMR (400 MHz, DMSO-*d_6_*): *δ* 12.58 (s, 1 H), 8.07 (d, *J* = 8.4 Hz, 1 H), 7.66 (d, *J* = 2.1 Hz, 1 H), 7.50 (dd, *J* = 8.6, 2.1 Hz, 1 H), 7.39 (d, *J* = 7.0 Hz, 2 H), 7.33 (t, *J* = 7.5 Hz, 2 H), 7.25 (t, *J* = 7.2 Hz, 1 H), 3.95 (s, 2 H). ^13^C NMR (100 MHz, DMSO-*d_6_*): *δ* 161.8, 158.1, 150.5, 139.5, 136.8, 129.4, 129.0, 128.3, 127.3, 127.0, 126.6, 120.1, 41.2. HRMS (ESI-TOF): *m*/*z* [M + H]^+^ calcd for C_15_H_12_OClN_2_^+^: 271.0632; found: 271.0630.
*2-benzyl-7-bromoquinazolin-4(3H)-one* (**5j**), purification on a silica gel (petroleum ether/ethyl acetate = 3:1) afforded compound **5j** as a white solid (100.9 mg, 64% yield); ^1^H NMR (400 MHz, DMSO-*d_6_*): *δ* 12.58 (s, 1 H), 7.99 (d, *J* = 8.4 Hz, 1 H), 7.81 (d, *J* = 2.1 Hz, 1 H), 7.62 (dd, *J* = 8.4, 2.0 Hz, 1 H), 7.39 (d, *J* = 6.8 Hz, 2 H), 7.33 (t, *J* = 7.5 Hz, 2 H), 7.25 (t, *J* = 7.2 Hz, 1 H), 3.95 (s, 2 H). ^13^C NMR (100 MHz, DMSO-*d_6_*): *δ* 161.9, 158.1, 150.6, 136.8, 129.7, 129.7, 129.4, 129.0, 128.5, 128.3, 127.3, 120.4, 41.2. HRMS (ESI-TOF): *m*/*z* [M + H]^+^ calcd for C_15_H_12_OBrN_2_^+^: 315.0127; found: 315.0131.
*2-(4-chlorobenzyl)quinazolin-4(3H)-one* (**5k**), purification on a silica gel (petroleum ether/ethyl acetate = 2:1) afforded compound **5k** as a white solid (89.4 mg, 66% yield); ^1^H NMR (400 MHz, DMSO-*d_6_*): *δ* 12.43 (s, 1 H), 8.09 (dd, *J* = 7.9, 1.6 Hz, 1 H), 7.78 (ddd, *J* = 8.6, 7.1, 1.6 Hz, 1 H), 7.60 (dd, *J* = 8.3, 1.1 Hz, 1 H), 7.50–7.45 (m, 1 H), 7.44–7.37 (m, 4 H), 3.95 (s, 2 H). ^13^C NMR (100 MHz, DMSO-*d_6_*): *δ* 162.3, 156.1, 149.3, 136.0, 134.9, 132.0, 131.3, 128.9, 127.4, 126.8, 126.2, 121.3, 40.4. HRMS (ESI-TOF): *m*/*z* [M + H]^+^ calcd for C_15_H_12_OClN_2_^+^: 271.0632; found: 271.0633.
*2-(4-bromobenzyl)quinazolin-4(3H)-one* (**5l**), purification on a silica gel (petroleum ether/ethyl acetate = 3:1) afforded compound **5l** as a white solid (99.3 mg, 63% yield); ^1^H NMR (400 MHz, DMSO-*d_6_*): *δ* 12.43 (s, 1 H), 8.09 (d, *J* = 9.6 Hz, 1 H), 7.82–7.73 (m, 1 H), 7.60 (d, *J* = 7.4 Hz, 1 H), 7.53 (d, *J* = 8.4 Hz, 2 H), 7.48 (t, *J* = 7.5 Hz, 1 H), 7.35 (d, *J* = 8.5 Hz, 2 H), 3.93 (s, 2 H). ^13^C NMR (100 MHz, DMSO-*d_6_*): *δ* 162.3, 156.0, 149.3, 136.4, 134.9, 131.8, 131.7, 127.4, 126.8, 126.2, 121.3, 120.5, 40.5. HRMS (ESI-TOF): *m*/*z* [M + H]^+^ calcd for C_15_H_12_OBrN_2_^+^: 315.0127; found: 315.0125.
*(E)-2-(prop-1-en-1-yl)quinazolin-4(3H)-one* (**5m**). Purification on a silica gel (petroleum ether/ethyl acetate = 1:1) afforded compound **5m** as a white solid (44.7 mg, 48% yield); ^1^H NMR (500 MHz, DMSO-*d_6_*): *δ* 12.17 (s, 1 H), 8.14–8.01 (m, 1 H), 7.82–7.72 (m, 1 H), 7.60 (d, *J* = 8.1 Hz, 1 H), 7.44 (t, *J* = 7.5 Hz, 1 H), 7.21–7.04 (m, 1 H), 6.27 (dd, *J* = 15.7, 1.7 Hz, 1 H), 1.92 (dd, *J* = 6.9, 1.5 Hz, 3 H). ^13^C NMR (125 MHz, DMSO-*d_6_*): *δ* 162.3, 151.6, 149.5, 138.7, 134.9, 127.5, 126.4, 126.2, 125.1, 121.4, 18.7. HRMS (ESI-TOF): *m*/*z* [M + H]^+^ calcd for C_11_H_11_ON_2_^+^: 187.0865; found: 187.0867.

## 4. Conclusions

To conclude, we disclosed an iodine-mediated one-pot procedure for the synthesis of pyrrolo/indolo[1,2-*a*]quinoxalines and quinazolin-4-ones under metal-free conditions. This methodology tandems the Meinwald rearrangement and annulation process. A series of *N*-heterocycles were obtained with medium to good yields. The protocol has a broad substrate scope, has a simple work-up and was suitable for wide-scale preparation. Furthermore, the obtained products could be further modified to afford promising pharmaceutical reagents. This study injects new vitality into the methodology for the synthesis of *N*-heterocycles. Related research is still in progress in our laboratory.

## Figures and Tables

**Table 1:** Optimization ^a^.

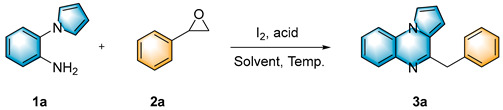				
Entry	Acid/Equiv.	Solvent	Temp. (°C)	Yield (%) ^b^
1	AcOH/1.0	CH_3_CN	120	52
2	HCOOH/1.0	CH_3_CN	120	55
3	TFA/1.0	CH_3_CN	120	56
4	TfOH/1.0	CH_3_CN	120	63
5	TsOH/1.0	CH_3_CN	120	76
6	TsOH/1.0	CH_3_CN	120	27 ^c^
7	-	CH_3_CN	120	50
8	TsOH/0.5	CH_3_CN	120	56r
9	TsOH/1.5	CH_3_CN	120	71
10	TsOH/1.0	DCE	120	47
11	TsOH/1.0	EtOH	120	43
12	TsOH/1.0	toluene	120	trace
13	TsOH/1.0	NMP	120	48
14	TsOH/1.0	PhCl	120	26
15	TsOH/1.0	CH_3_CN	100	62
16	TsOH/1.0	CH_3_CN	80	58

^a^ Reaction conditions: **1a** (0.5 mmol, 1 equiv.), **2a** (1.0 mmol, 2 equiv.), I_2_ (0.5 mmol, 1 equiv.) and acid in solvent (1.0 mL) were stirred in a sealed tube and allowed to react for 4 h. ^b^ Isolated yield. ^c^ Without I_2_.

**Table 2:** Substrate Scope of 2-(1*H*-Pyrrolo/indolo-1-yl)anilines ^a,b^.

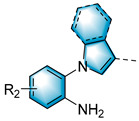	+	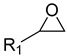	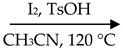	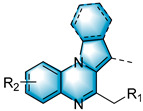	
**1**		**2**		**3**	
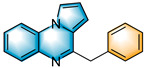	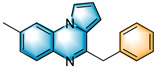		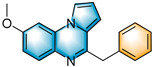	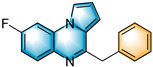	
**3a**, 76%	**3b**, 62%		**3c**, 59%	**3d**, 74%	
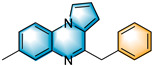	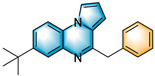		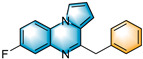	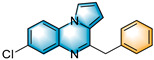	
**3e**, 62%	**3f**, 73%		**3g**, 71%	**3h**, 63%	
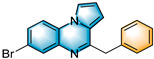	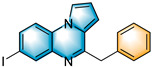		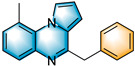	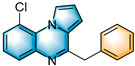	
**3i**, 41%	**3j**, 40%		**3k**, 64%	**3l**, 65%	
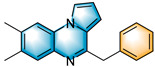	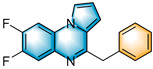		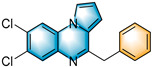	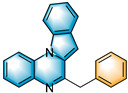	
**3m**, 64%	**3n**, 76%		**3o**, 65%	**3p**, 48%	
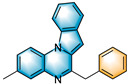	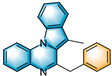		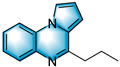	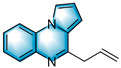	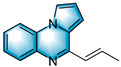
**3q**, 38%	**3r**, 35%		**3s**, 40%	**3t’**	**3t**, 50%

^a^ **1** (0.5 mmol, 1 equiv.), **2** (1.0 mmol, 2 equiv.), I_2_ (0.5 mmol, 1 equiv.) and acid (0.5 mmol, 1 equiv.) in solvent (1.0 mL) were stirred in a sealed tube, and allowed to react for 4 h. ^b^ Isolated yield.

**Table 3:** Substrate Scope of 2-Aminobenzamide ^a,b^.

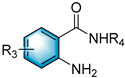	+	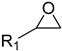	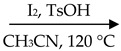	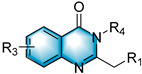
**4**		**2**		**5**
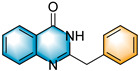	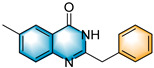		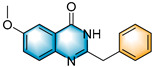	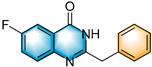
**5a**, 70%	**5b**, 74%		**5c**, 58%	**5d**, 71%
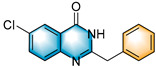	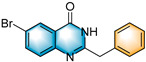		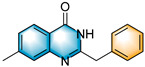	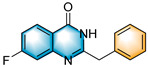
**5e**, 75%	**5f**, 59%		**5g**, 60%	**5h**, 67%
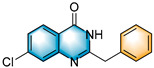	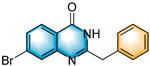		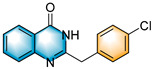	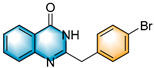
**5i**, 65%	**5j**, 64%		**5k**, 60%	**5l**, 65%
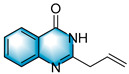	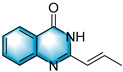		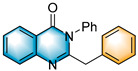	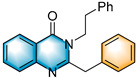
**5m** **’**	**5m**, 48%		**5n, 0%**	**5o, 0%**

^a^ **4** (0.5 mmol, 1 equiv.), **2** (1.0 mmol, 2 equiv.), I_2_ (0.5 mmol, 1 equiv.) and acid (0.5 mmol, 1 equiv.) in solvent (1.0 mL) were stirred in a sealed tube and allowed to react for 4 h. ^b^ Isolated yield.

## Data Availability

The supporting data are available in the Supplementary Materials.

## Supplementary Materials

The following supporting information can be downloaded at https://www.mdpi.com/article/10.3390/molecules28217391/s1, Figure S1: ^1^H NMR spectrum of compound **3a** in CDCl3 (400 MHz); Figure S2: ^13^C {^1^H} NMR spectrum of compound **3a** in CDCl_3_ (100 MHz); Figure S3: ^1^H NMR spectrum of compound **3b** in CDCl_3_ (400 MHz); Figure S4: ^13^C {1 H} NMR spectrum of compound **3b** in CDCl_3_ (100 MHz); Figure S5: ^1^H NMR spectrum of compound **3c** in CDCl_3_ (400 MHz); Figure S6: ^13^C {^1^ H} NMR spectrum of compound **3c** in CDCl_3_ (100 MHz); Figure S7: ^1^H NMR spectrum of compound **3d** in CDCl_3_ (400 MHz); Figure S8: ^13^C {^1^ H} NMR spectrum of compound **3d** in CDCl_3_ (100 MHz); Figure S9: ^19^F {^1^ H} NMR spectrum of compound **3d** in CDCl_3_ (377 MHz); Figure S10: ^1^H NMR spectrum of compound **3e** in CDCl_3_ (400 MHz); Figure S11: ^13^C {^1^ H} NMR spectrum of compound **3e** in CDCl_3_ (100 MHz); Figure S12: ^1^H NMR spectrum of compound **3f** in CDCl_3_ (400 MHz); Figure S13: ^13^C {^1^ H} NMR spectrum of compound **3f** in CDCl_3_ (100 MHz); Figure S14: ^1^H NMR spectrum of compound **3g** in CDCl_3_ (400 MHz); Figure S15: ^13^C {^1^ H} NMR spectrum of compound **3g** in CDCl_3_ (100 MHz); Figure S16: ^19^F {^1^H} NMR spectrum of compound **3g** in CDCl_3_ (377 MHz); Figure S17: ^1^H NMR spectrum of compound **3h** in CDCl_3_ (400 MHz); Figure S18: ^13^C {^1^ H} NMR spectrum of compound **3h** in CDCl_3_ (100 MHz); Figure S19: ^1^H NMR spectrum of compound **3i** in CDCl_3_ (400 MHz); Figure S20: ^13^C {^1^ H} NMR spectrum of compound **3i** in CDCl_3_ (100 MHz); Figure S21: ^1^H NMR spectrum of compound **3j** in CDCl_3_ (400 MHz); Figure S22: ^13^C {^1^ H} NMR spectrum of compound **3j** in CDCl_3_ (100 MHz); Figure S23: ^1^H NMR spectrum of compound **3k** in CDCl_3_ (400 MHz); Figure S24: ^13^C {^1^ H} NMR spectrum of compound **3k** in CDCl_3_ (100 MHz); Figure S25: ^1^H NMR spectrum of compound **3l** in CDCl_3_ (400 MHz); Figure S26: ^13^C {^1^ H} NMR spectrum of compound **3l** in CDCl_3_ (100 MHz); Figure S27: ^1^H NMR spectrum of compound **3m** in CDCl_3_ (400 MHz); Figure S28: ^13^C {^1^ H} NMR spectrum of compound **3m** in CDCl_3_ (100 MHz); Figure S29: ^1^H NMR spectrum of compound **3n** in CDCl_3_ (400 MHz); Figure S30: ^13^C {^1^ H} NMR spectrum of compound **3n** in CDCl_3_ (100 MHz); Figure S31: ^19^F {^1^ H} NMR spectrum of compound **3n** in CDCl_3_ (377 MHz); Figure S32: ^1^H NMR spectrum of compound **3o** in CDCl_3_ (400 MHz); Figure S33: ^13^C {^1^ H} NMR spectrum of compound **3o** in CDCl_3_ (100 MHz); Figure S34: ^1^H NMR spectrum of compound **3p** in CDCl_3_ (400 MHz); Figure S35: ^13^C {^1^ H} NMR spectrum of compound **3p** in CDCl_3_ (100 MHz); Figure S36: ^1^H NMR spectrum of compound **3q** in CDCl_3_ (400 MHz); Figure S37: ^13^C {^1^ H} NMR spectrum of compound **3q** in CDCl_3_ (100 MHz); Figure S38: ^1^H NMR spectrum of compound **3r** in CDCl_3_ (400 MHz); Figure S39: ^13^C {^1^ H} NMR spectrum of compound **3r** in CDCl_3_ (100 MHz); Figure S40: ^1^H NMR spectrum of compound **3s** in CDCl_3_ (500 MHz); Figure S41: ^13^C {^1^ H} NMR spectrum of compound **3s** in CDCl_3_ (125 MHz); Figure S42: ^1^H NMR spectrum of compound **3t** in CDCl_3_ (500 MHz); Figure S43: ^13^C {^1^ H} NMR spectrum of compound **3t** in CDCl_3_ (125 MHz); Figure S44: ^1^H NMR spectrum of compound **5a** in DMSO-*d_6_* (400 MHz); Figure S45: ^13^C {^1^ H} NMR spectrum of compound **5a** in DMSO-*d_6_* (100 MHz); Figure S46: ^1^H NMR spectrum of compound **5b** in DMSO-*d_6_* (400 MHz); Figure S47: ^13^C {^1^ H} NMR spectrum of compound **5b** in DMSO-*d_6_* (100 MHz); Figure S48: ^1^H NMR spectrum of compound **5c** in DMSO-*d_6_* (400 MHz); Figure S49: ^13^C {^1^ H} NMR spectrum of compound **5c** in DMSO-*d_6_* (100 MHz); Figure S50: ^1^H NMR spectrum of compound **5d** in DMSO-*d_6_* (400 MHz); Figure S51: ^13^C {^1^ H} NMR spectrum of compound **5d** in DMSO-*d_6_* (100 MHz); Figure S52: ^19^F {^1^ H} NMR spectrum of compound **5d** in DMSO-*d_6_* (377 MHz); Figure S53: ^1^H NMR spectrum of compound **5e** in DMSO-*d_6_* (400 MHz); Figure S54: ^13^C {^1^ H} NMR spectrum of compound **5e** in DMSO-*d_6_* (100 MHz); Figure S55: ^1^H NMR spectrum of compound **5f** in DMSO-*d_6_* (400 MHz); Figure S56: ^13^C {^1^ H} NMR spectrum of compound **5f** in DMSO-*d_6_* (100 MHz); Figure S57: ^1^H NMR spectrum of compound **5g** in DMSO-*d_6_* (400 MHz); Figure S58: ^13^C {^1^ H} NMR spectrum of compound **5g** in DMSO-*d_6_* (100 MHz); Figure S59: ^1^H NMR spectrum of compound **5h** in DMSO-*d_6_* (400 MHz); Figure S60: ^13^C {^1^ H} NMR spectrum of compound **5h** in DMSO-*d_6_* (100 MHz); Figure S61: ^19^F {^1^ H} NMR spectrum of compound **5h** in DMSO-*d_6_* (377 MHz); Figure S62: ^1^H NMR spectrum of compound **5i** in DMSO-*d_6_* (400 MHz); Figure S63: ^13^C {^1^ H} NMR spectrum of compound **5i** in DMSO-*d_6_* (100 MHz); Figure S64: ^1^H NMR spectrum of compound **5j** in DMSO-*d_6_* (400 MHz); Figure S65: ^13^C {^1^ H} NMR spectrum of compound **5j** in DMSO-*d_6_* (100 MHz); Figure S66: ^1^H NMR spectrum of compound **5k** in DMSO-*d_6_* (400 MHz); Figure S67: ^13^C {^1^ H} NMR spectrum of compound **5k** in DMSO-*d_6_* (100 MHz); Figure S68: ^1^H NMR spectrum of compound **5l** in DMSO-*d_6_* (400 MHz); Figure S69: ^13^C {^1^ H} NMR spectrum of compound **5l** in DMSO-*d_6_* (100 MHz); Figure S70: ^1^H NMR spectrum of compound **5m** in DMSO-*d_6_* (500 MHz); Figure S71: ^13^C {^1^ H} NMR spectrum of compound **5m** in DMSO-*d_6_* (125 MHz); Figure S72. HRMS of intermediate.
