# Supplementary material for: A Direct Method for Synthesis of Quinoxalines and Quinazolinones Using Epoxides as Alkyl Precursor
Source: Molecules. 2023 Nov 2;28(21):7391. doi: 10.3390/molecules28217391 (PMC10648482; doi:10.3390/molecules28217391)
Supplement: Supplementary file 1 [file molecules-28-07391-s001.zip › molecules-2664668-supplementary.pdf]

A Direct Method for Synthesis of Quinoxalines and Quinazolinones Using Epoxides as Alkyl Precursor

Xueyan Lv [1], Lili Lv [2], Shichen Li [1], Chengcheng Ding [1], Bingchuan Yang [3,*] and Chen Ma [1,*]

[1] Key Laboratory of Special Functional Aggregated Materials, Ministry of Education, School of Chemistry and Chemical Engineering, Shandong University, Jinan 250100, China; lxy17852267584@163.com (X.L.); ray19940519@foxmail.com (S.L.); 202220349@mail.sdu.edu.cn (C.D.)

[2] China Petroleum Planning and Engineering Institute, Dongying 257237, China; lvlili@petrochina.com.cn

[3] College of Chemistry and Chemical Engineering, Qilu Normal University, Jinan 250013, China

* Correspondence: yangbingchuan@lcu.edu.cn (B.Y.); chenma@sdu.edu.cn (C.M.)

Table of contents

1. Copies of ^{1}H NMR and ^{13}C NMR spectra……………………………………………1

2. HRMS of intermediate 34

1. Copies of ^{1}H NMR and ^{13}C NMR spectra

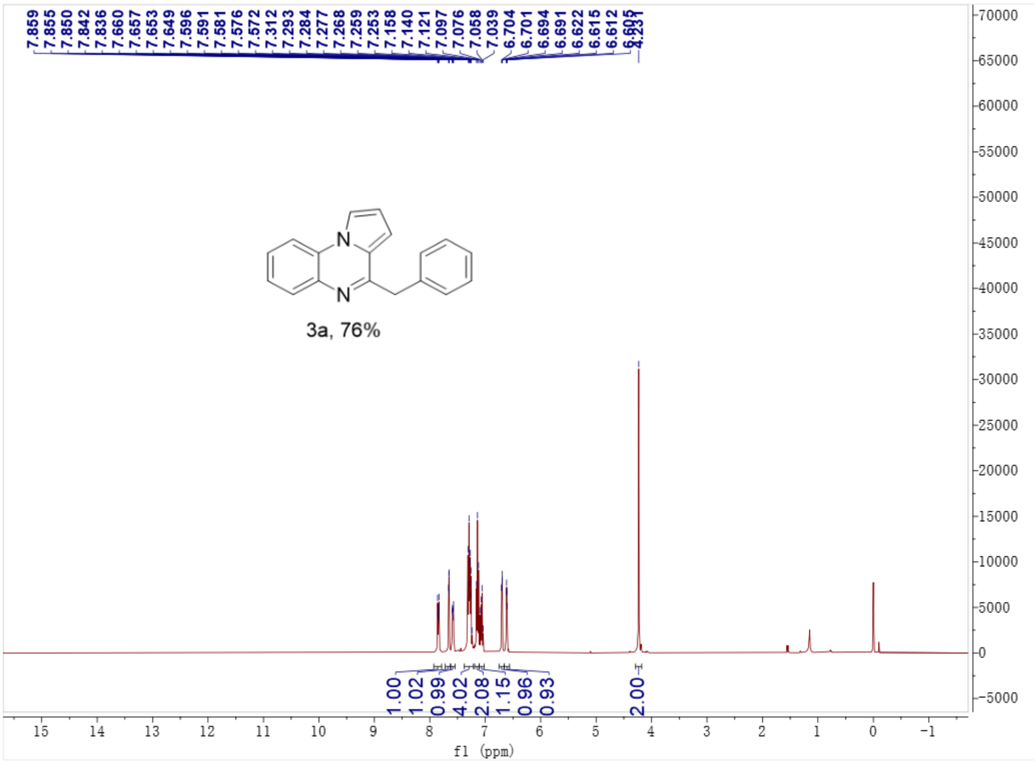

Figure S1. ^{1}H NMR spectrum of compound **3a** in CDCl3 (400 MHz).

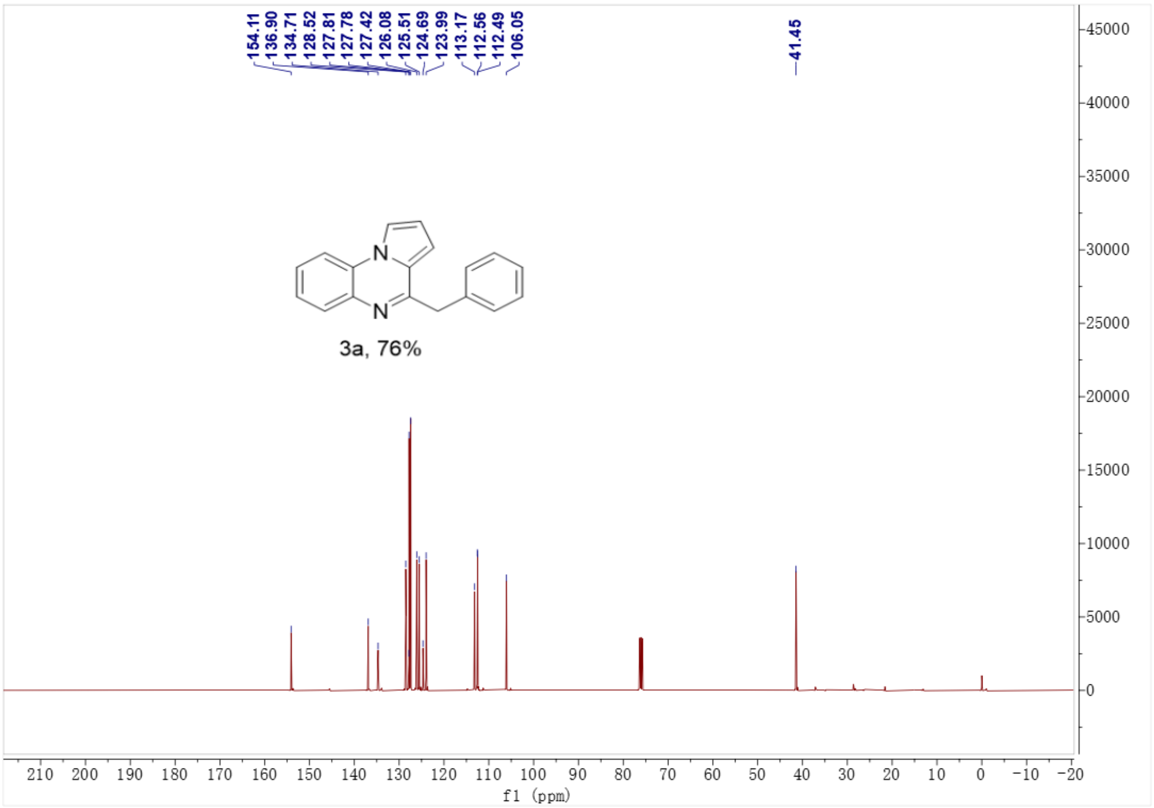

Figure S2. ^{13}C {^{1}H} NMR spectrum of compound **3a** in $CDCl_3$ (100 MHz).

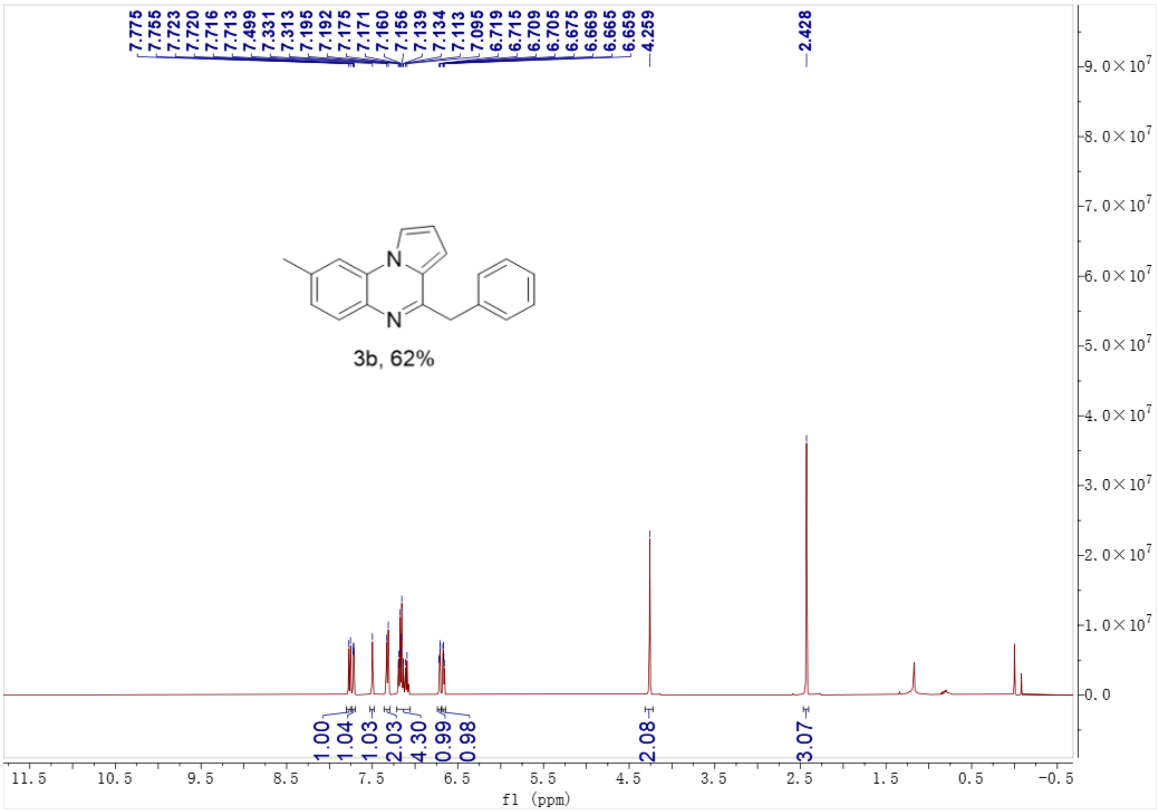

Figure S3. ^{1}H NMR spectrum of compound **3b** in $CDCl_3$ (400 MHz).

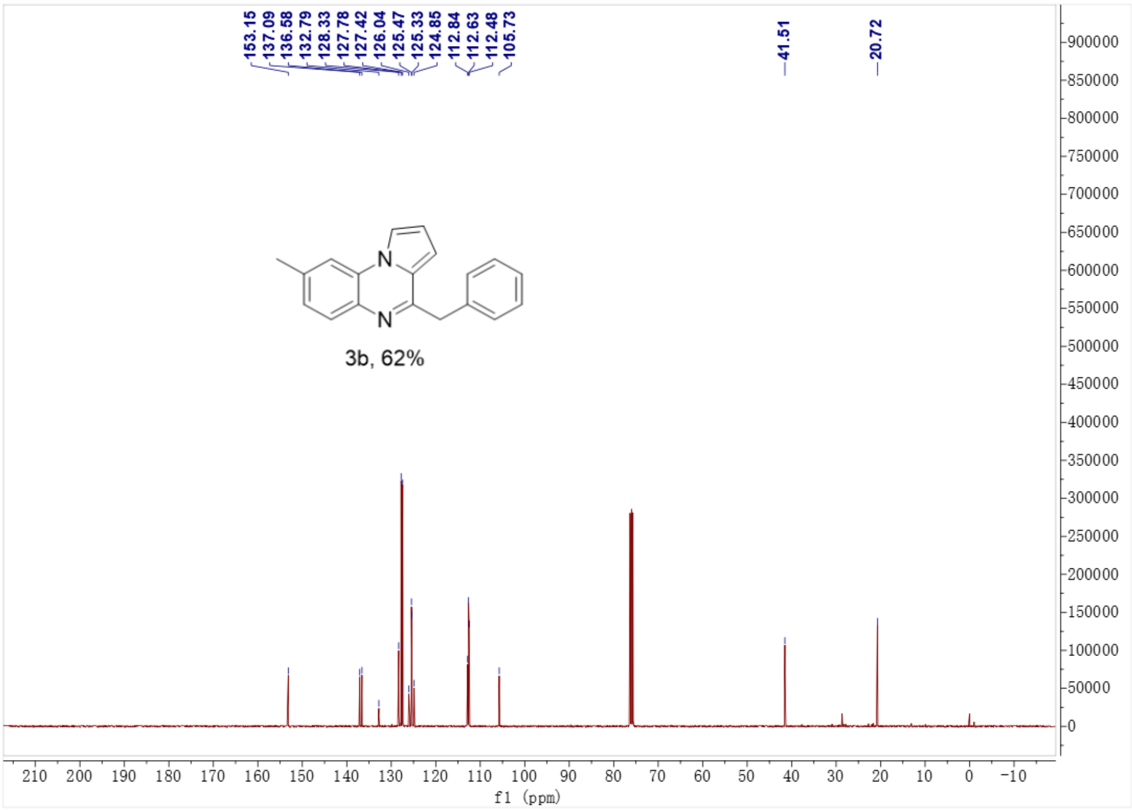

Figure S4. ^{13}C {1 H} NMR spectrum of compound **3b** in $CDCl_3$ (100 MHz).

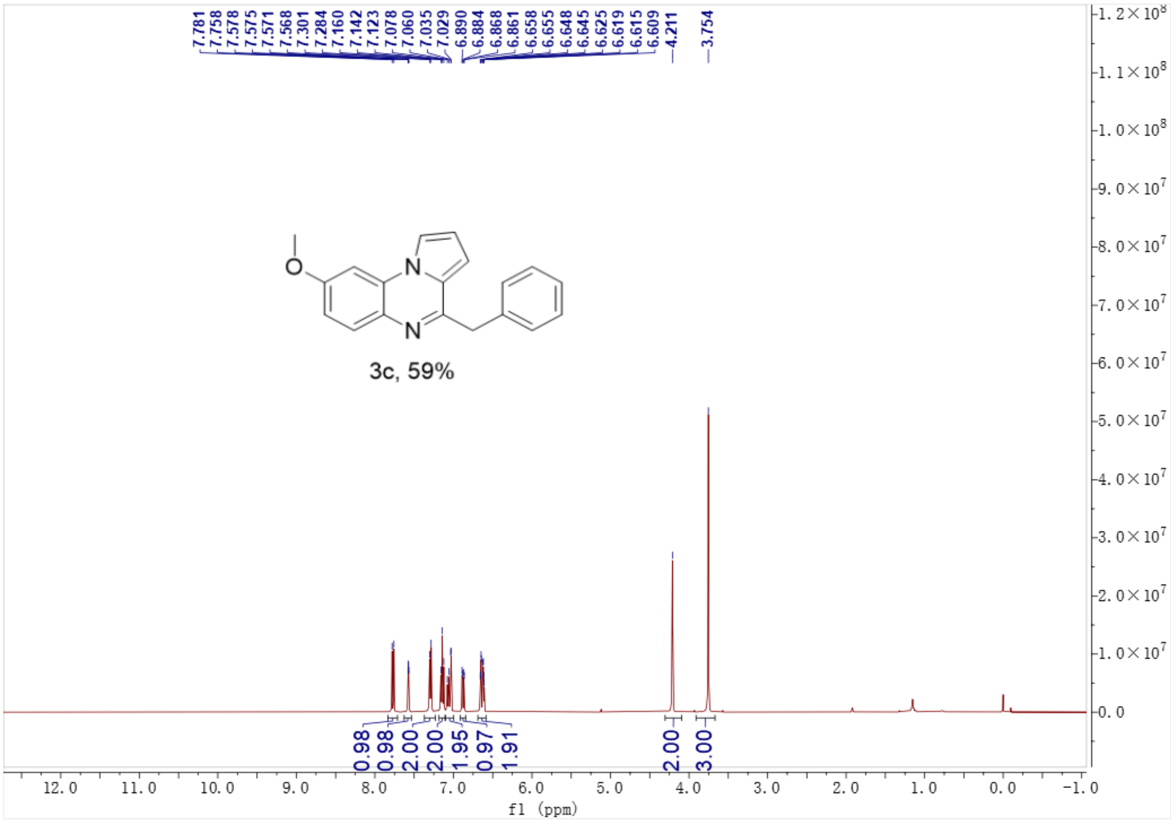

Figure S5. ^{1}H NMR spectrum of compound **3c** in $CDCl_3$ (400 MHz).

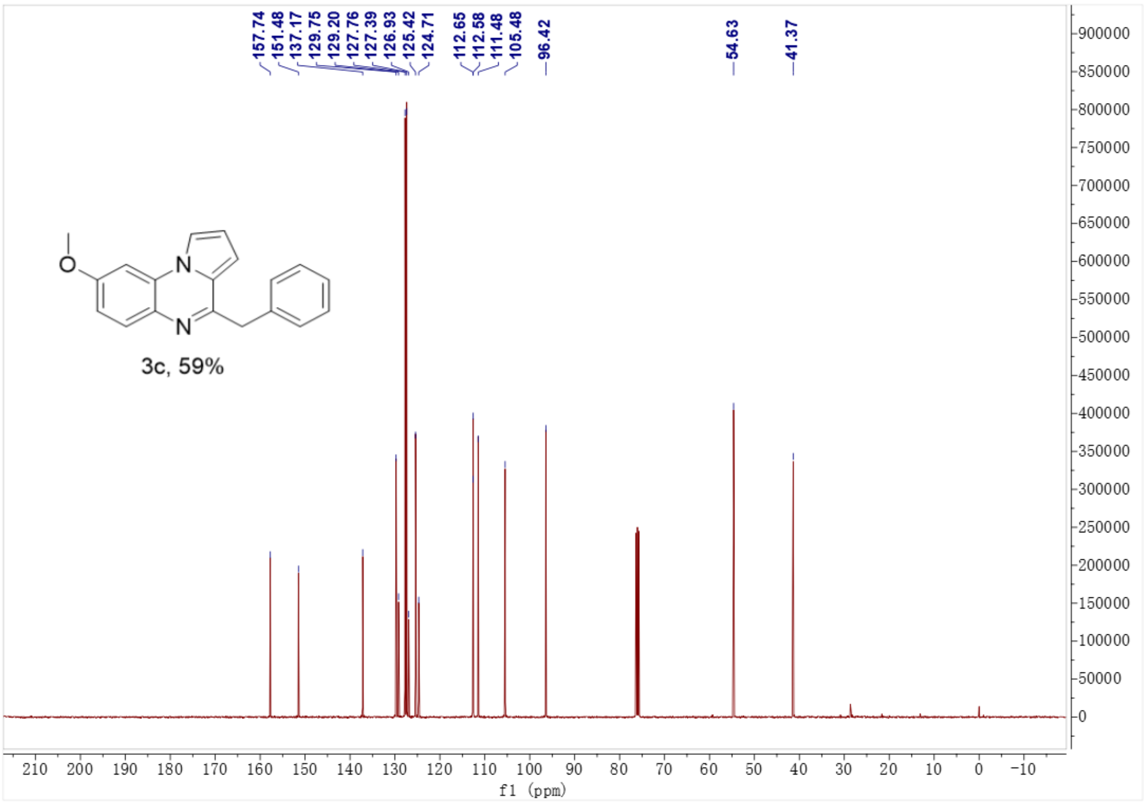

Figure S6. ^{13}C {[1] H} NMR spectrum of compound **3c** in $CDCl_3$ (100 MHz).

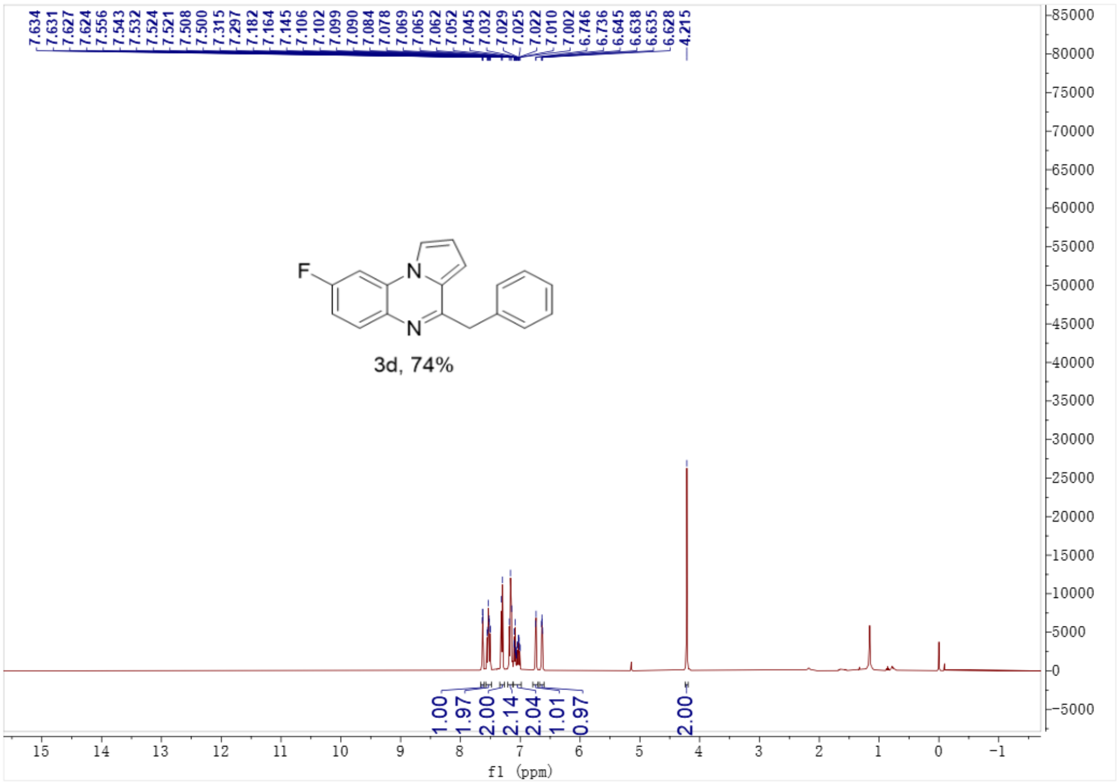

Figure S7. ^{1}H NMR spectrum of compound **3d** in $CDCl_3$ (400 MHz).

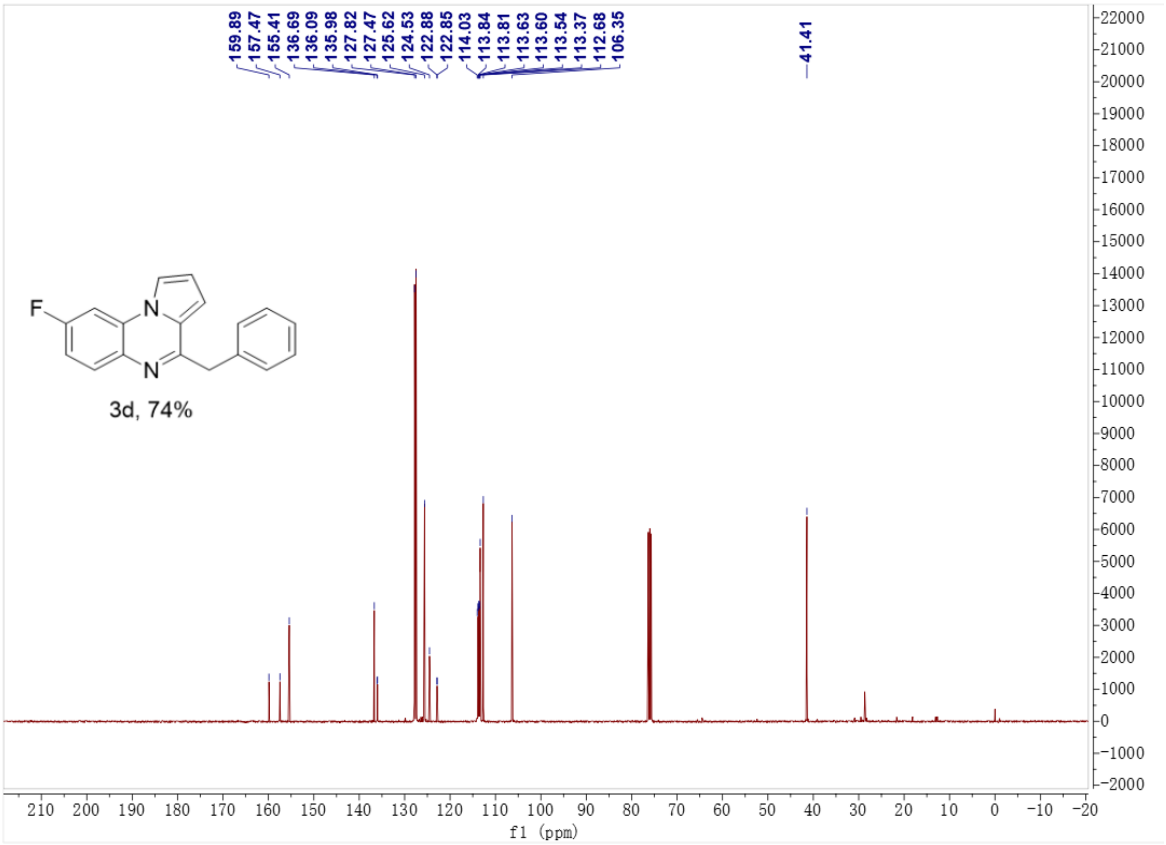

Figure S8. $^{13}C\{^{1}H\}$ NMR spectrum of compound **3d** in $CDCl_3$ (100 MHz).

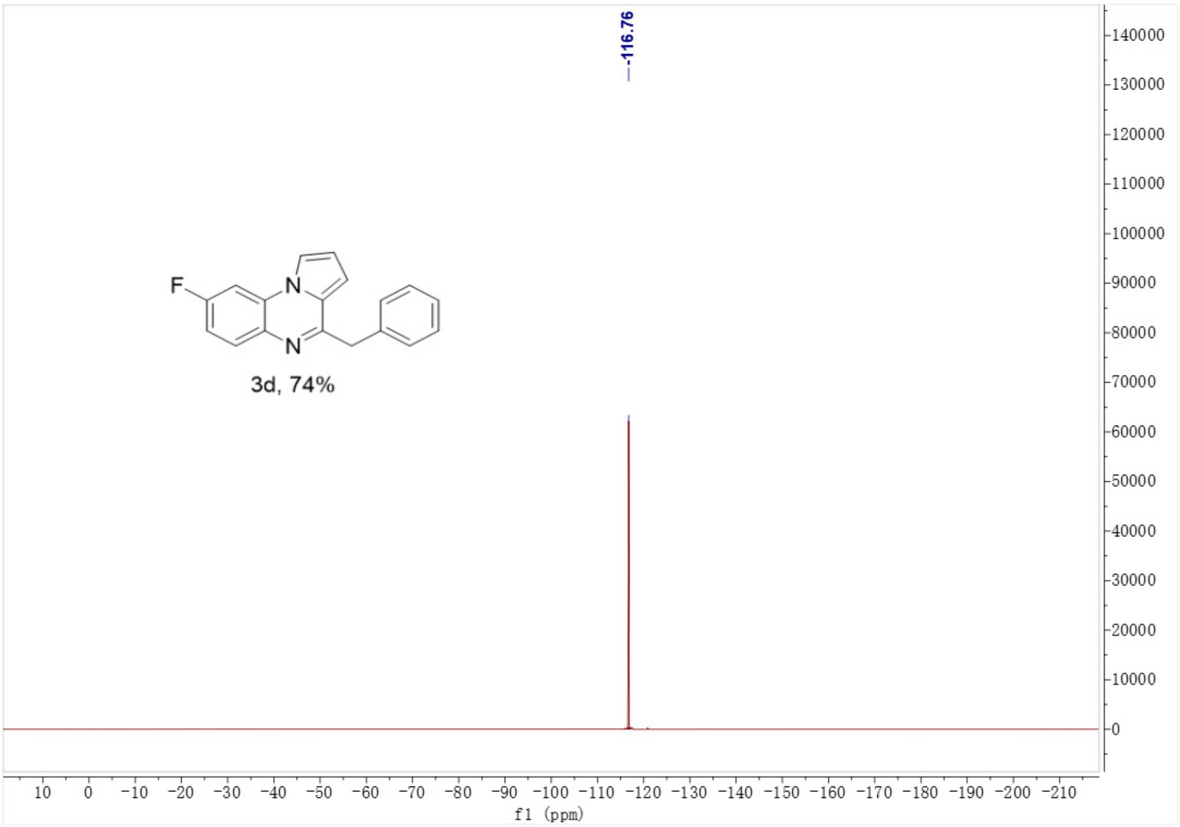

Figure S9. $^{19}F\{^{1}H\}$ NMR spectrum of compound **3d** in $CDCl_3$ (377 MHz)

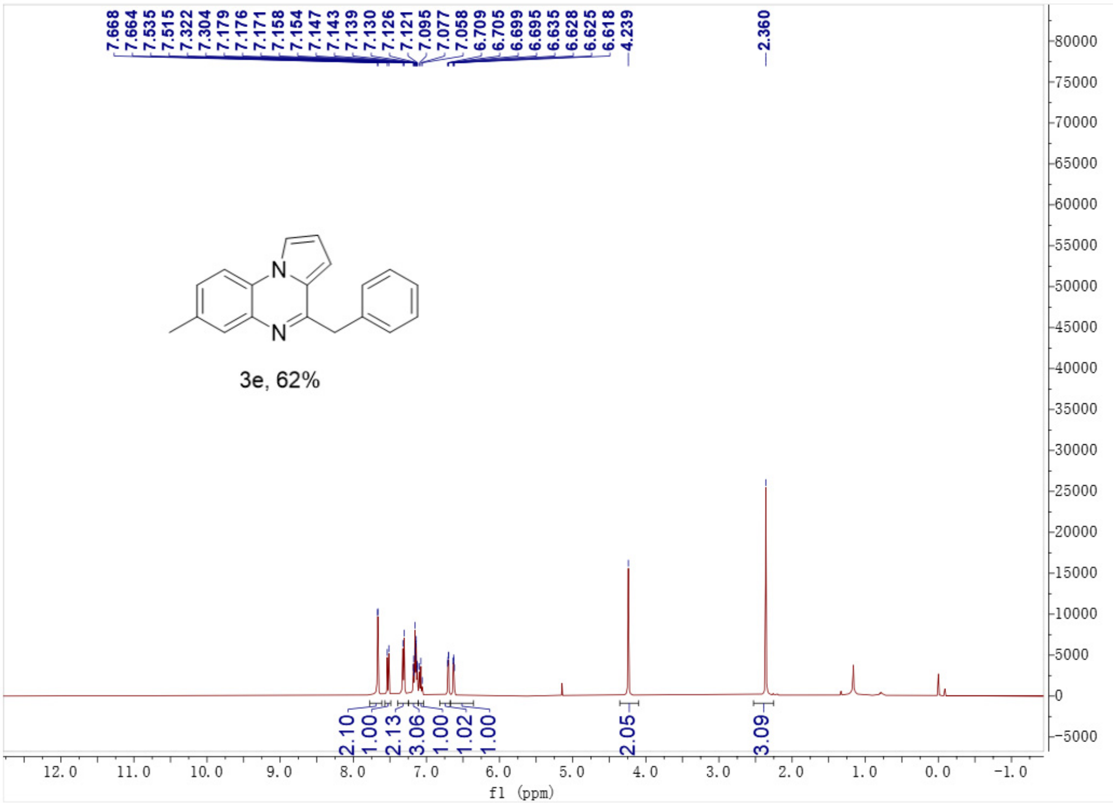

Figure S10. ^{1}H NMR spectrum of compound **3e** in $CDCl_3$ (400 MHz).

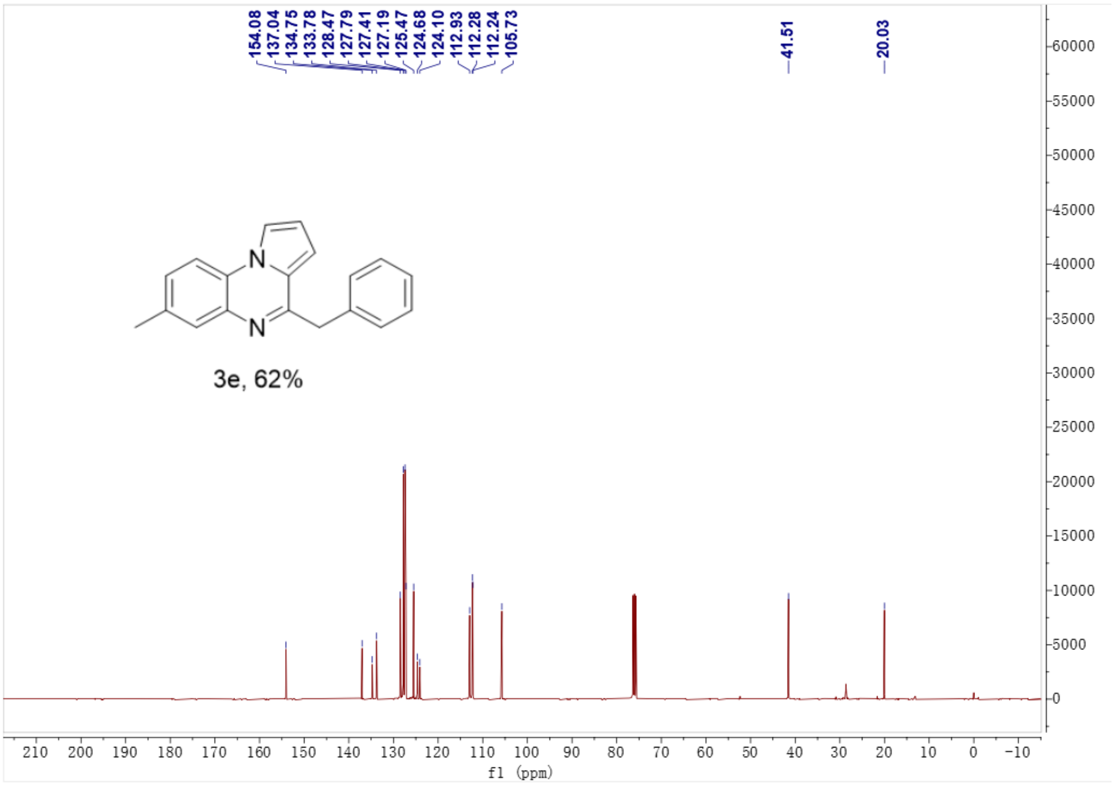

Figure S11. ^{13}C {^{1}H} NMR spectrum of compound **3e** in $CDCl_3$ (100 MHz).

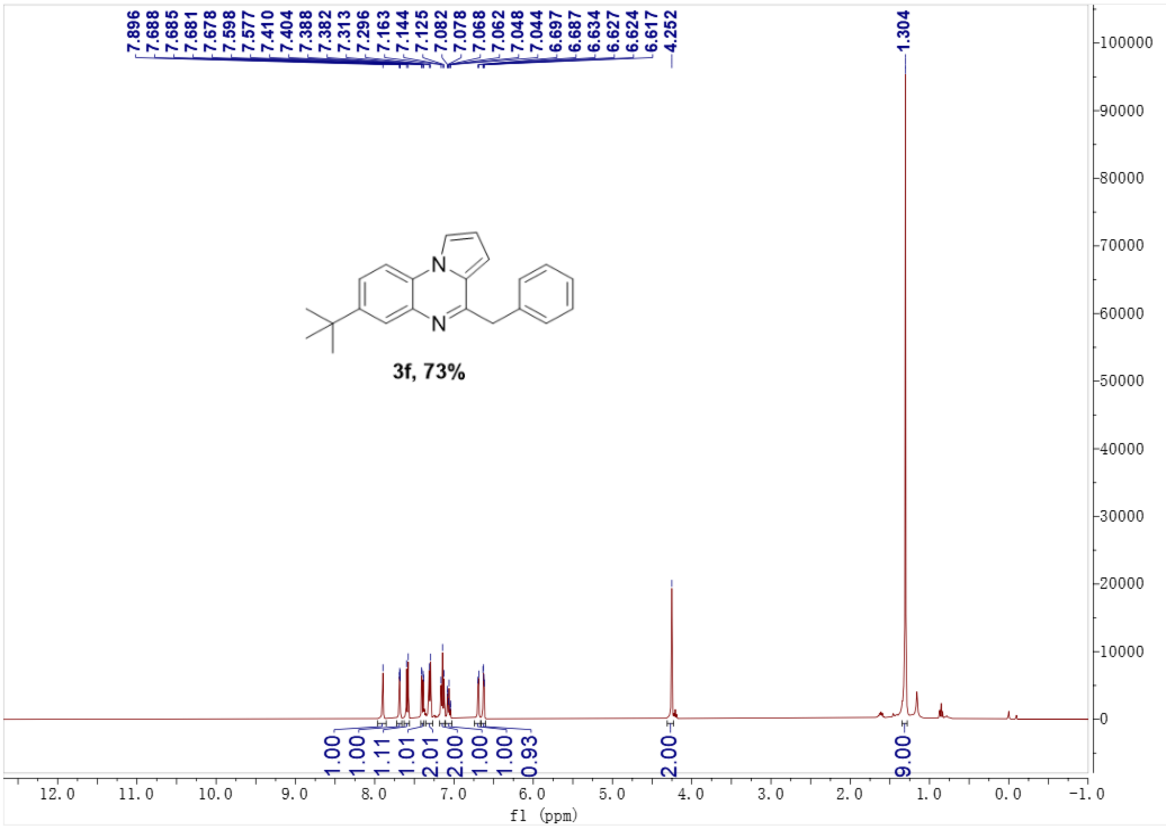

Figure S12. ^{1}H NMR spectrum of compound **3f** in $CDCl_3$ (400 MHz).

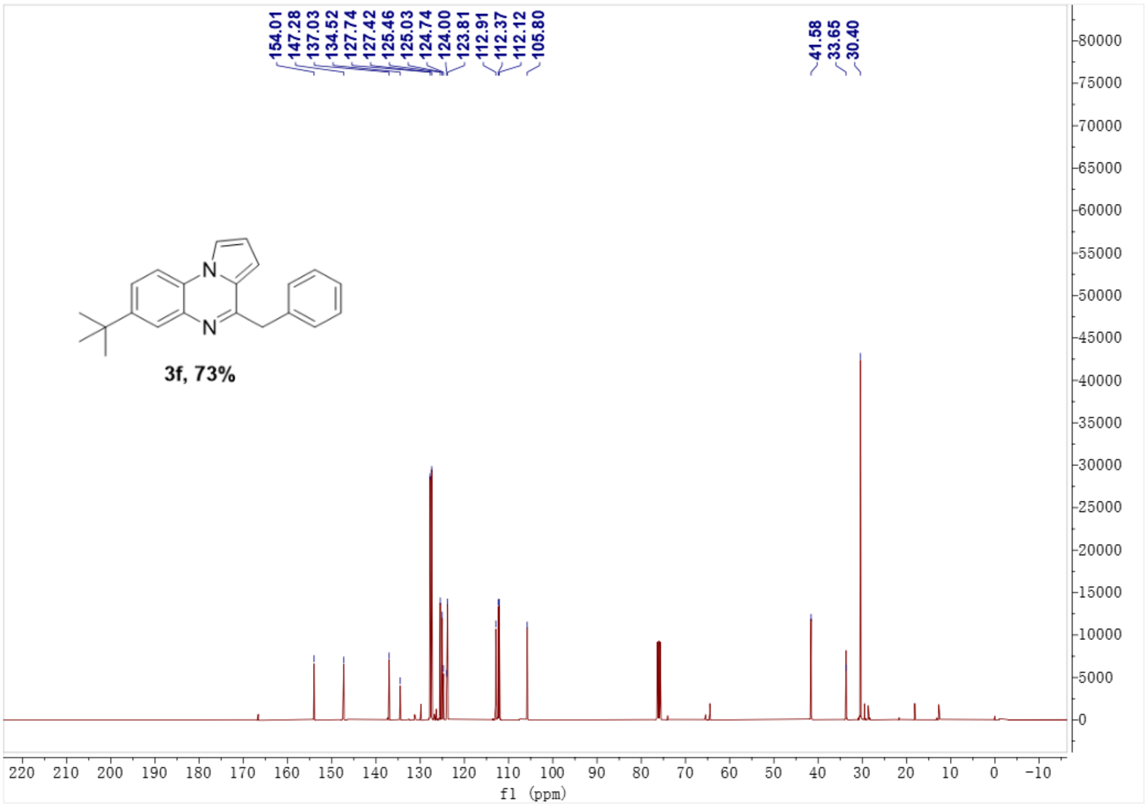

Figure S13. ^{13}C {[1] H} NMR spectrum of compound **3f** in $CDCl_3$ (100 MHz).

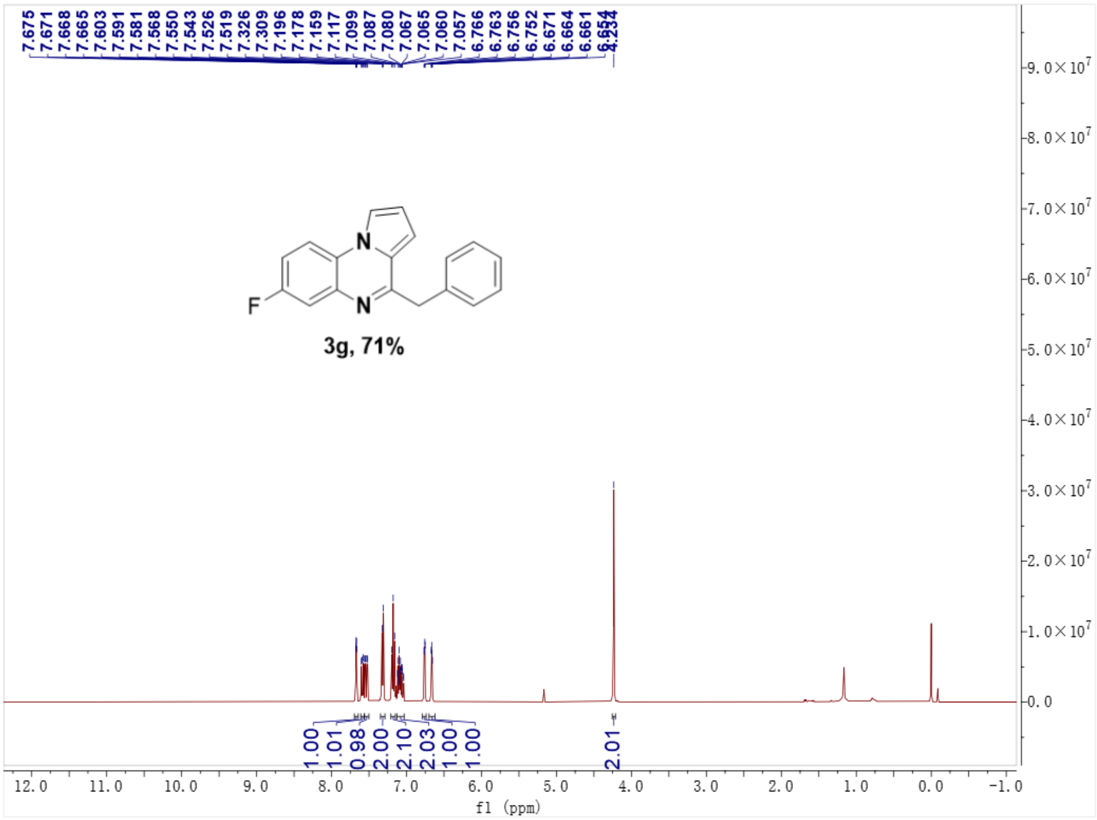

Figure S14. ^{1}H NMR spectrum of compound **3g** in $CDCl_3$ (400 MHz).

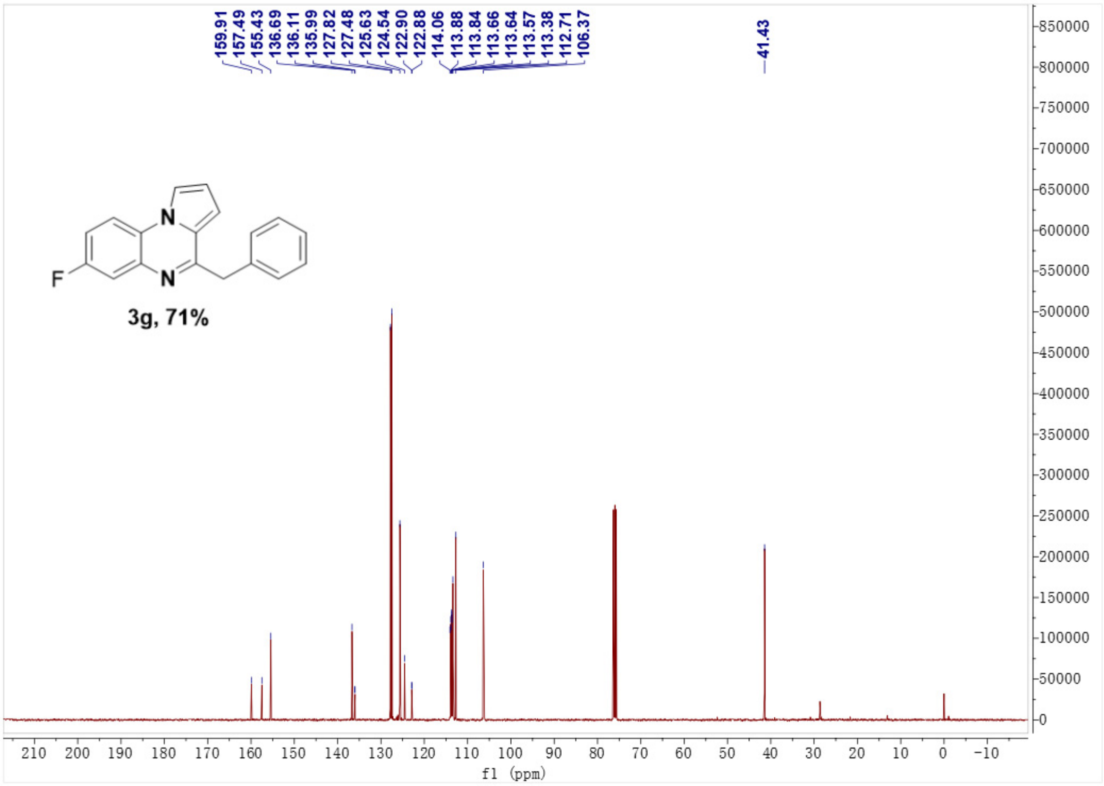

Figure S15. ^{13}C {^{1} H} NMR spectrum of compound **3g** in $CDCl_3$ (100 MHz)

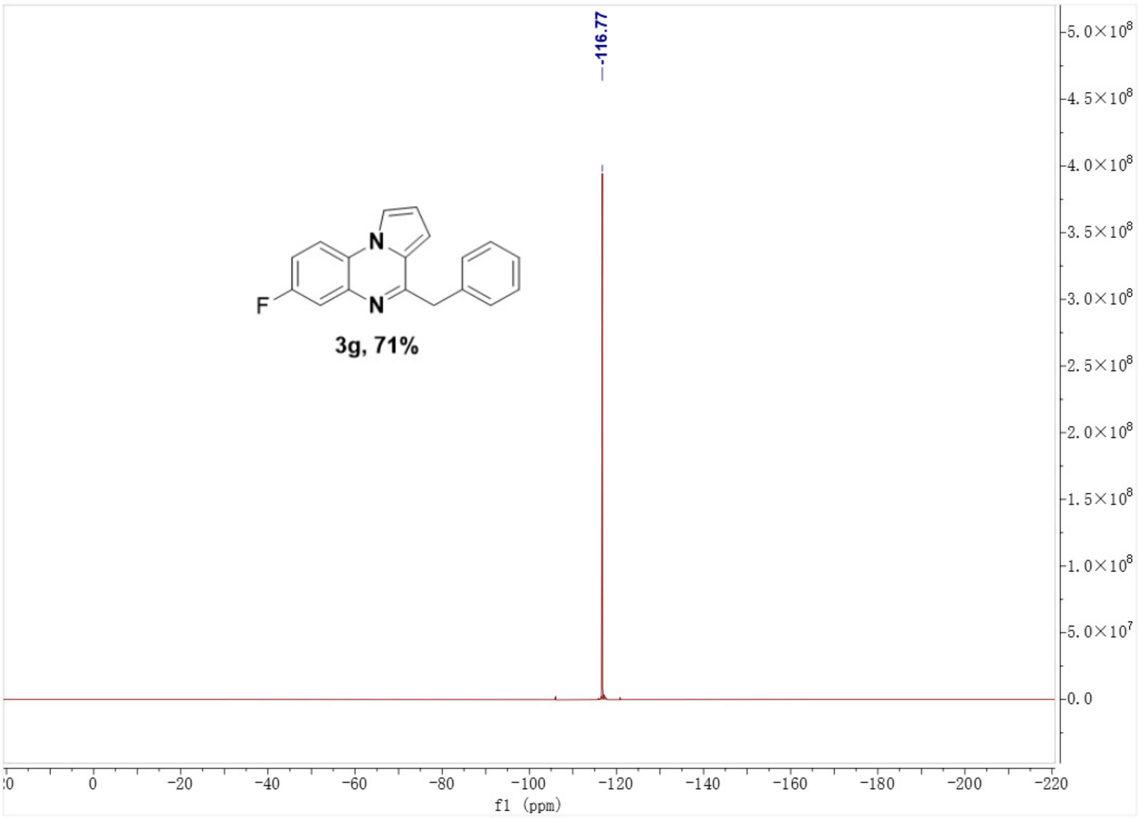

Figure S16. ^{19}F {^{1}H} NMR spectrum of compound **3g** in $CDCl_3$ (377 MHz)

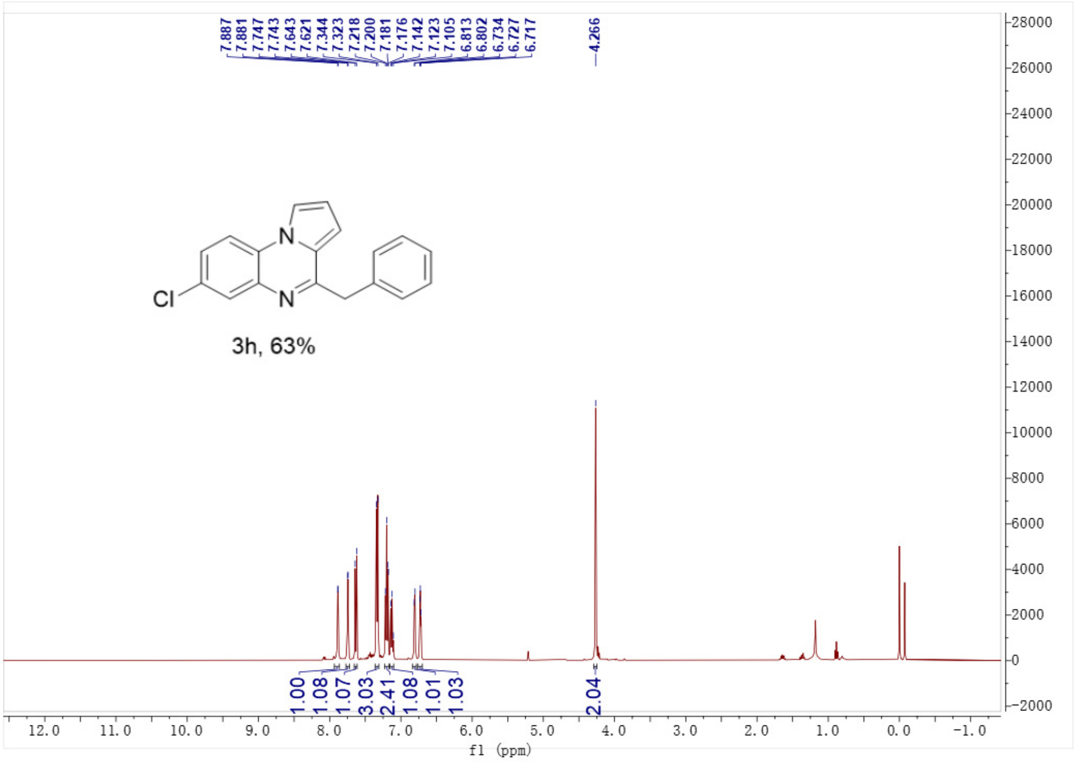

Figure S17. ^{1}H NMR spectrum of compound **3h** in $CDCl_3$ (400 MHz).

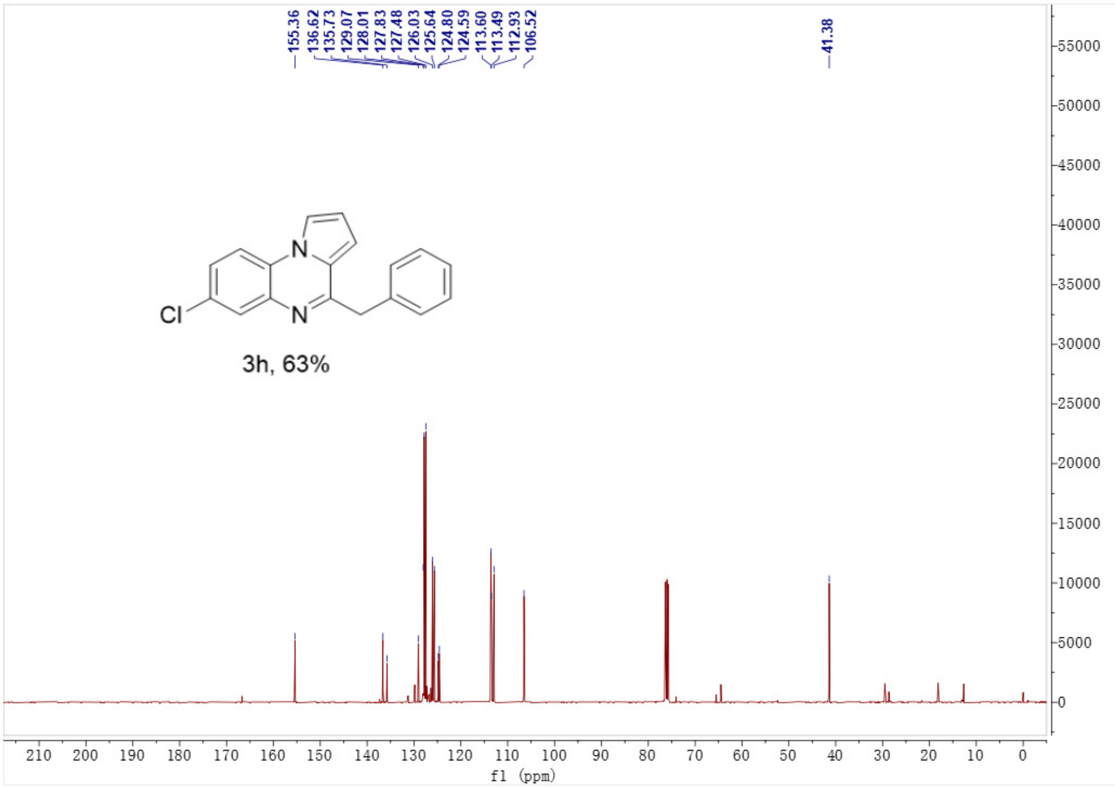

Figure S18. ^{13}C {^{1} H} NMR spectrum of compound **3h** in $CDCl_3$ (100 MHz).

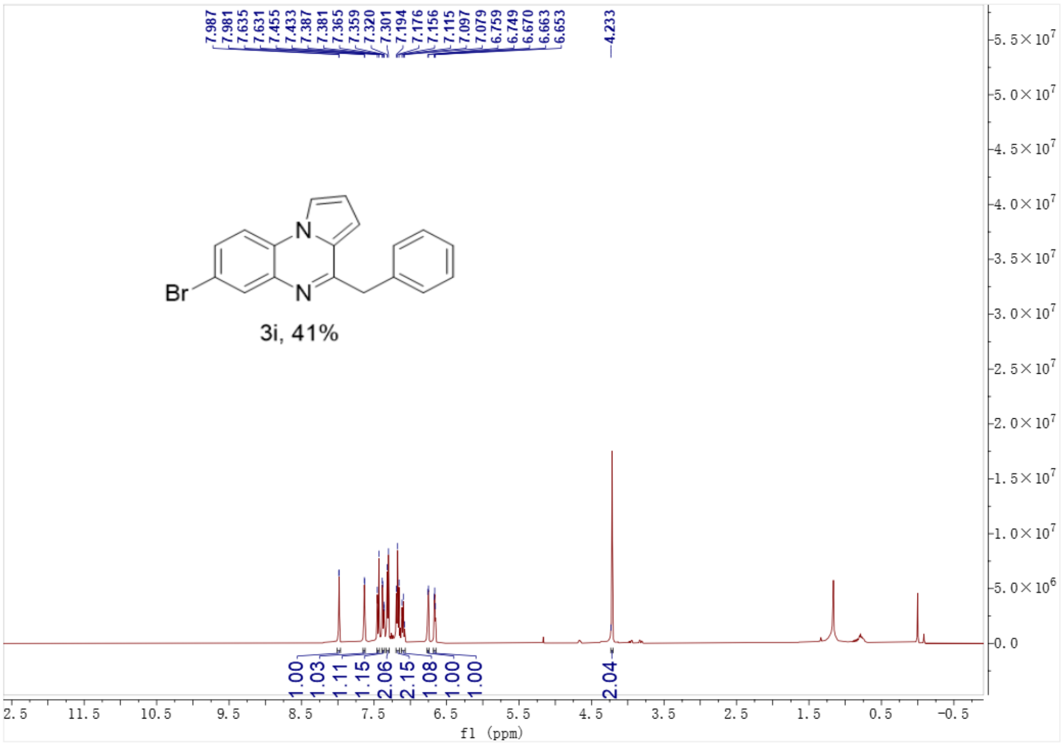

Figure S19. ^{1}H NMR spectrum of compound **3i** in $CDCl_3$ (400 MHz).

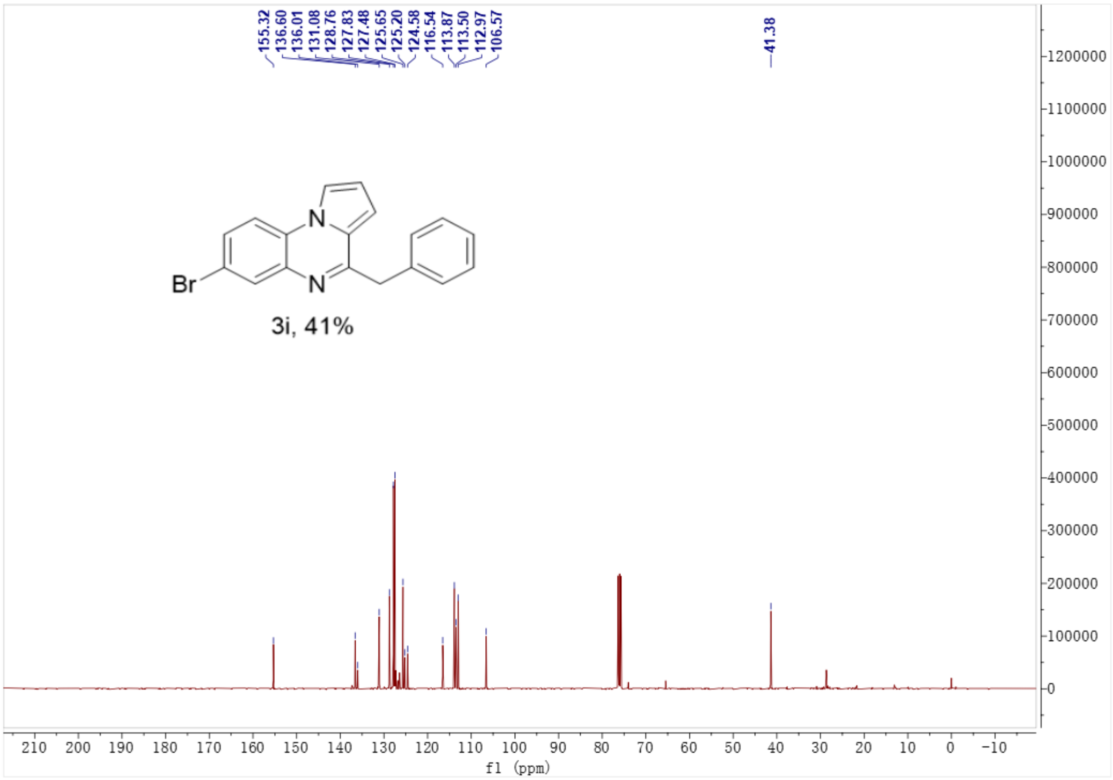

Figure S20. ^{13}C {^{1} H} NMR spectrum of compound **3i** in $CDCl_3$ (100 MHz).

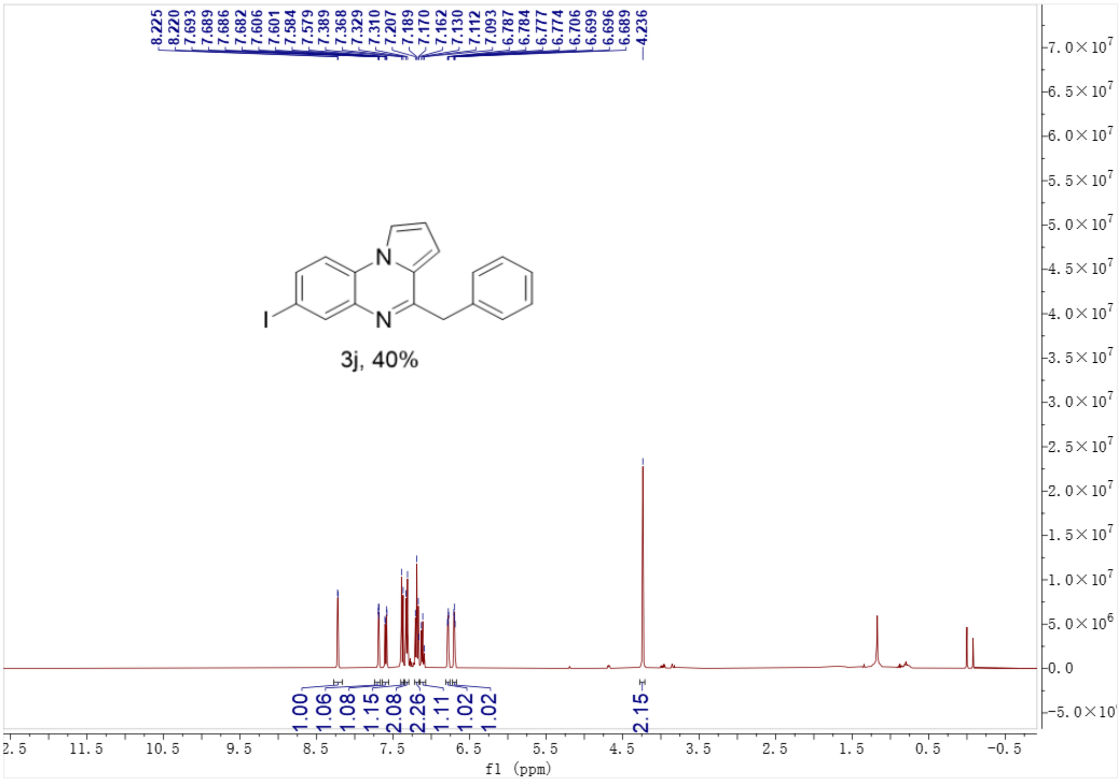

Figure S21. ^{1}H NMR spectrum of compound **3j** in $CDCl_3$ (400 MHz).

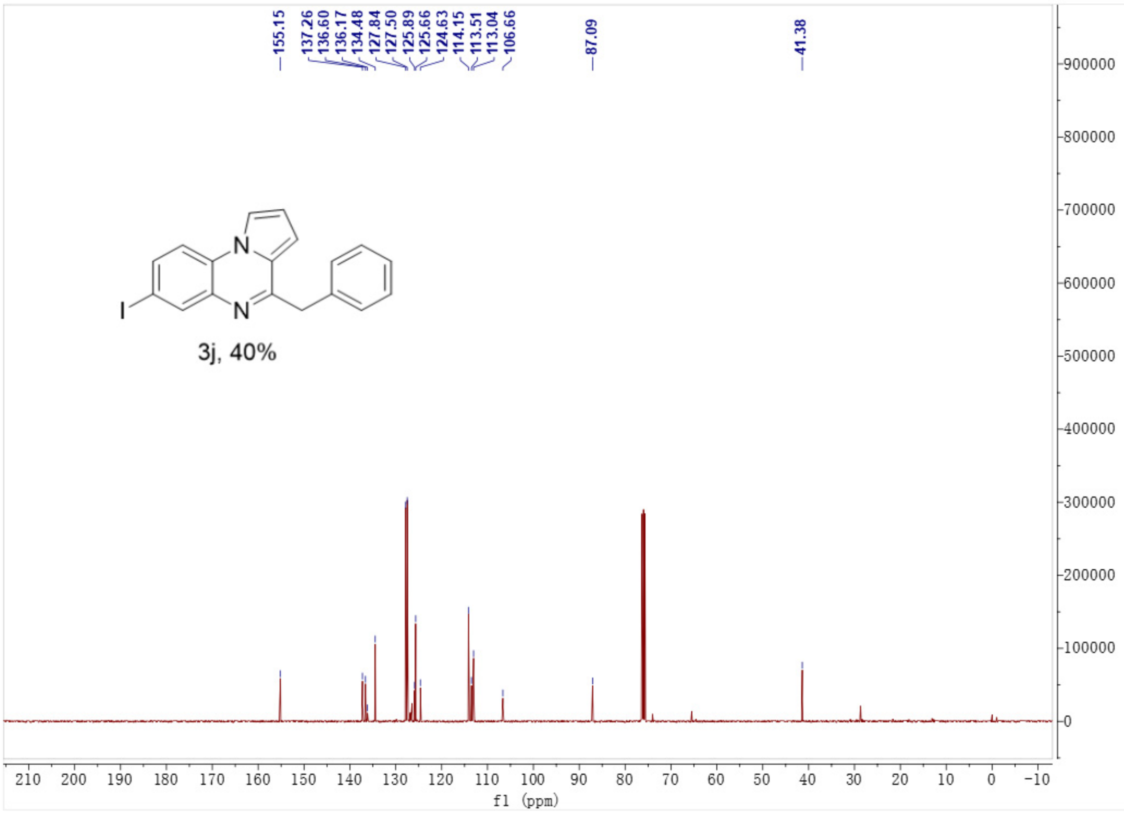

Figure S22. ^{13}C {[1] H} NMR spectrum of compound **3j** in $CDCl_3$ (100 MHz).

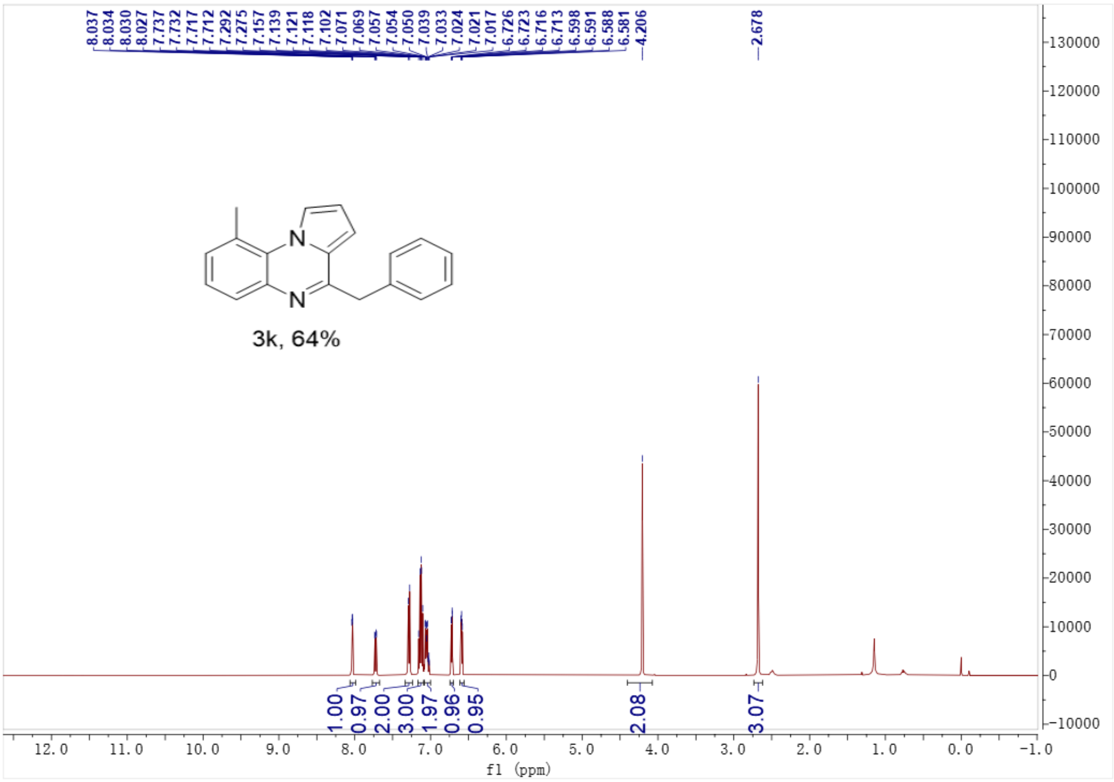

Figure S23. ^{1}H NMR spectrum of compound **3k** in $CDCl_3$ (400 MHz).

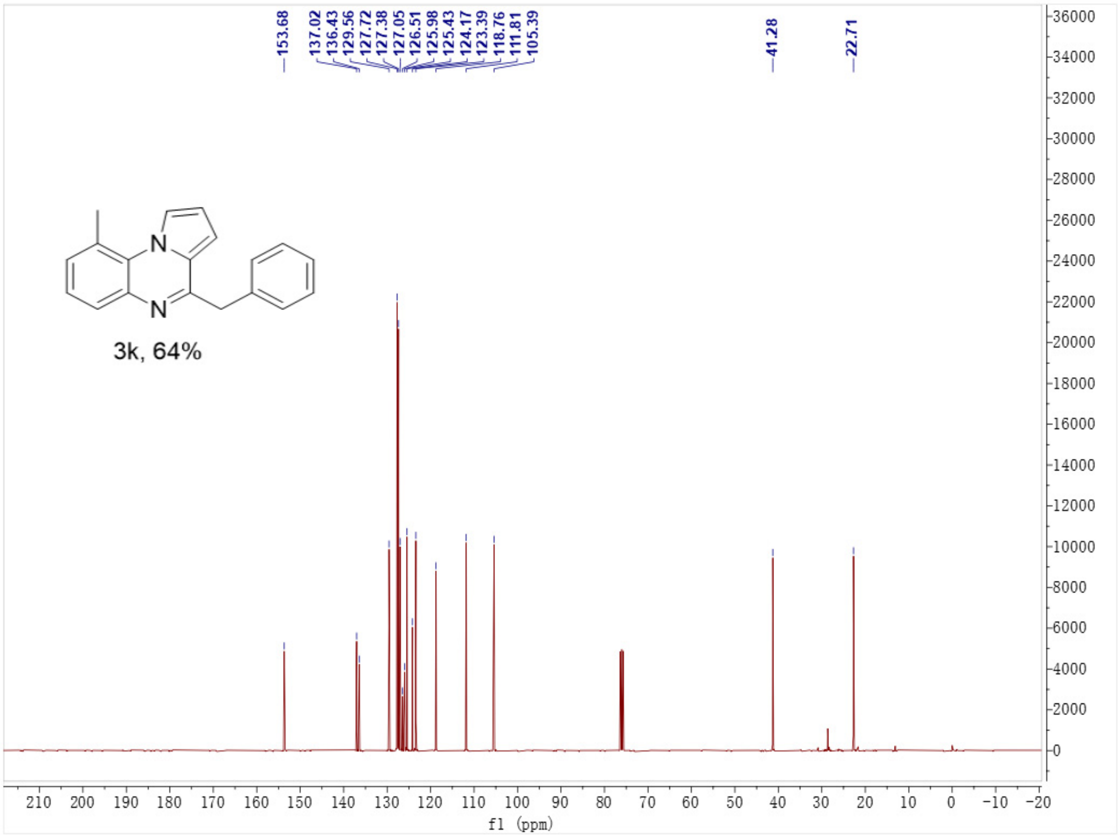

Figure S24. ^{13}C {[1] H} NMR spectrum of compound **3k** in $CDCl_3$ (100 MHz).

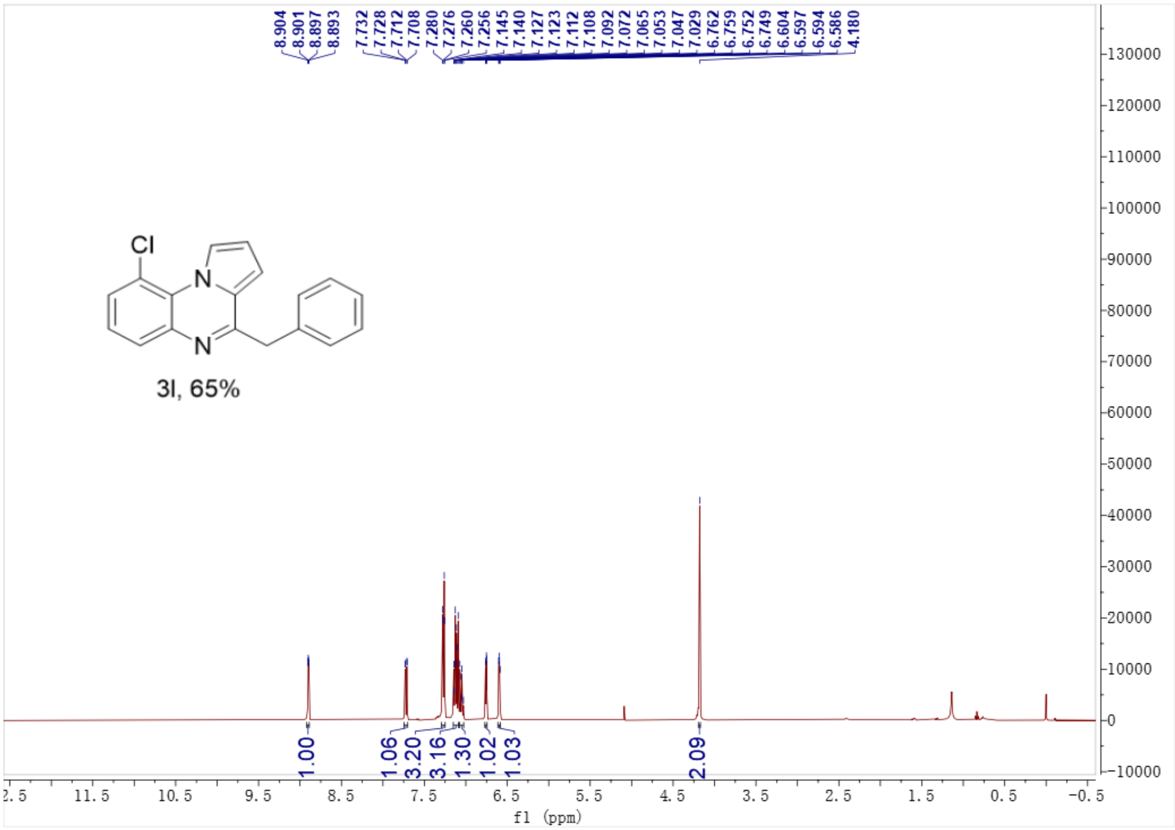

Figure S25. ^{1}H NMR spectrum of compound **3l** in $CDCl_3$ (400 MHz).

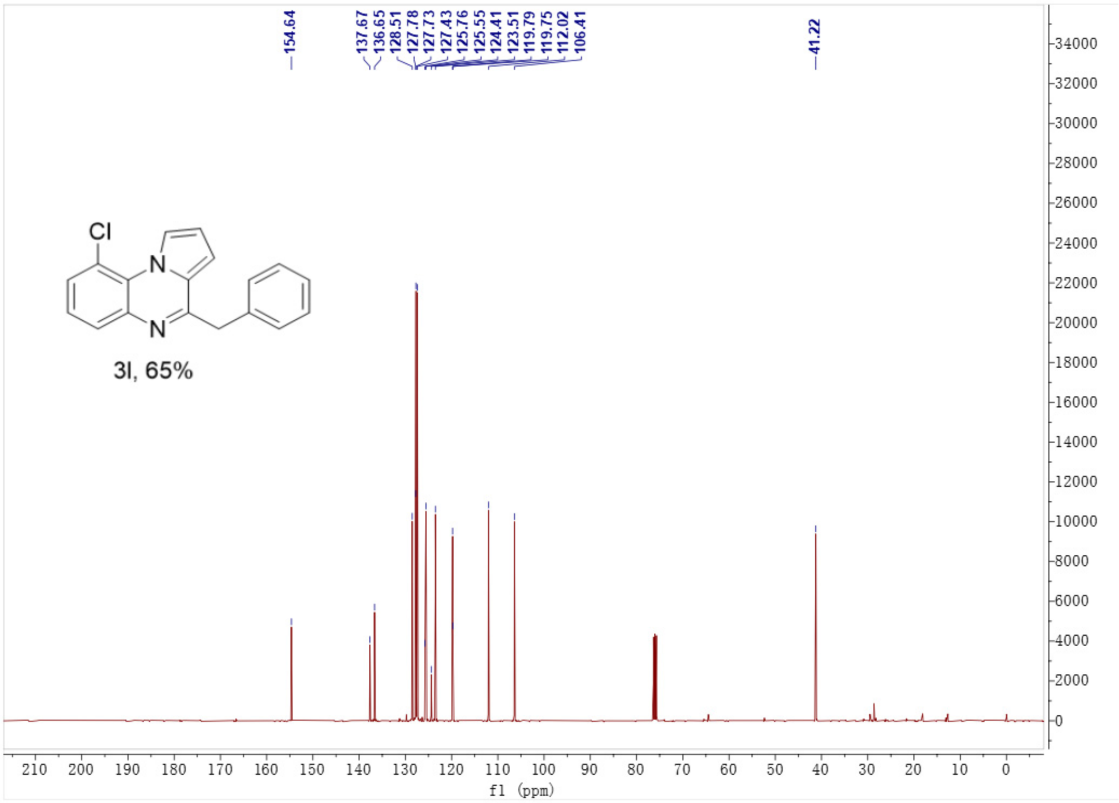

Figure S26. ^{13}C {[1] H} NMR spectrum of compound **3l** in $CDCl_3$ (100 MHz).

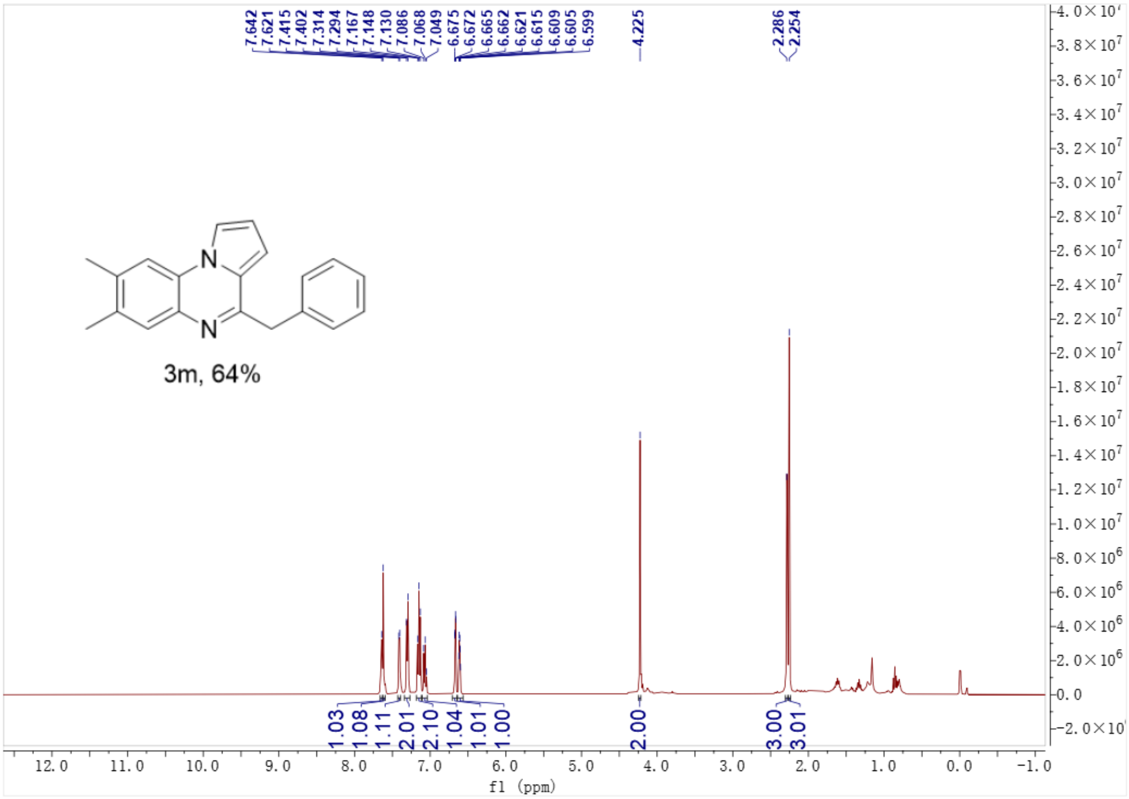

Figure S27. ^{1}H NMR spectrum of compound **3m** in $CDCl_3$ (400 MHz).

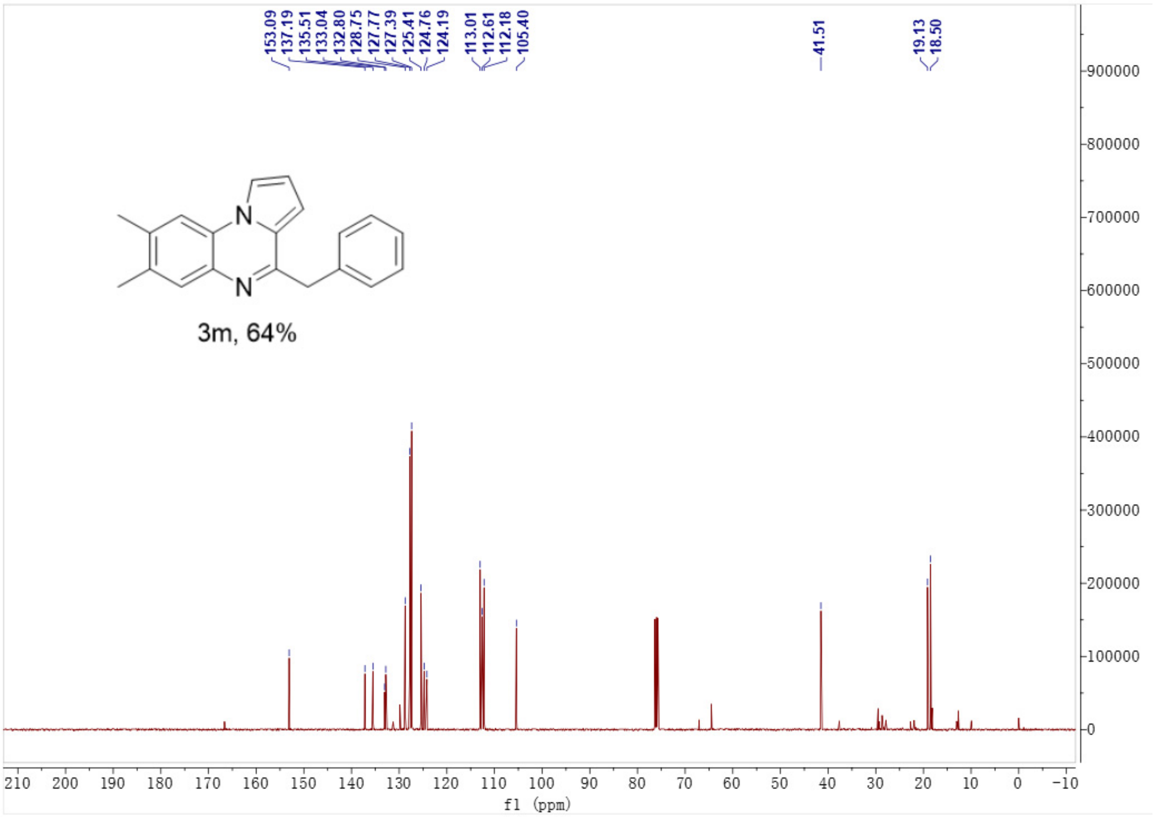

Figure S28. ^{13}C {[1] H} NMR spectrum of compound **3m** in $CDCl_3$ (100 MHz).

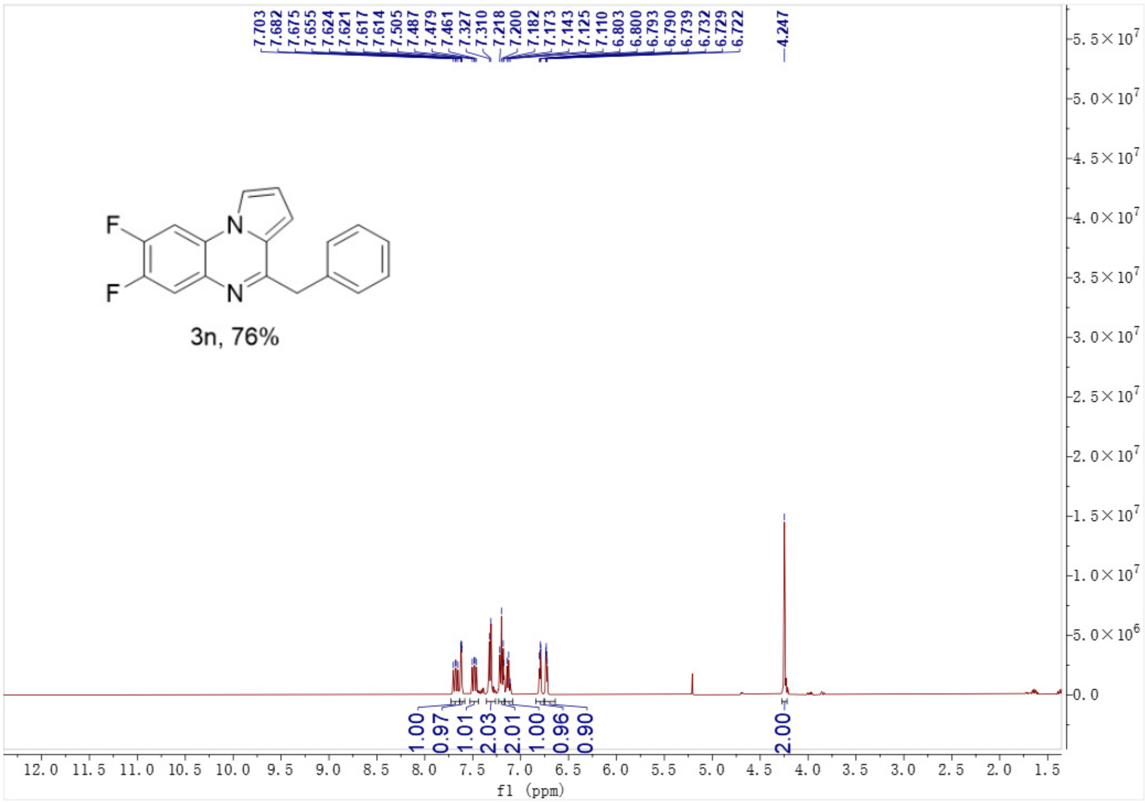

Figure S29. ^{1}H NMR spectrum of compound **3n** in $CDCl_3$ (400 MHz).

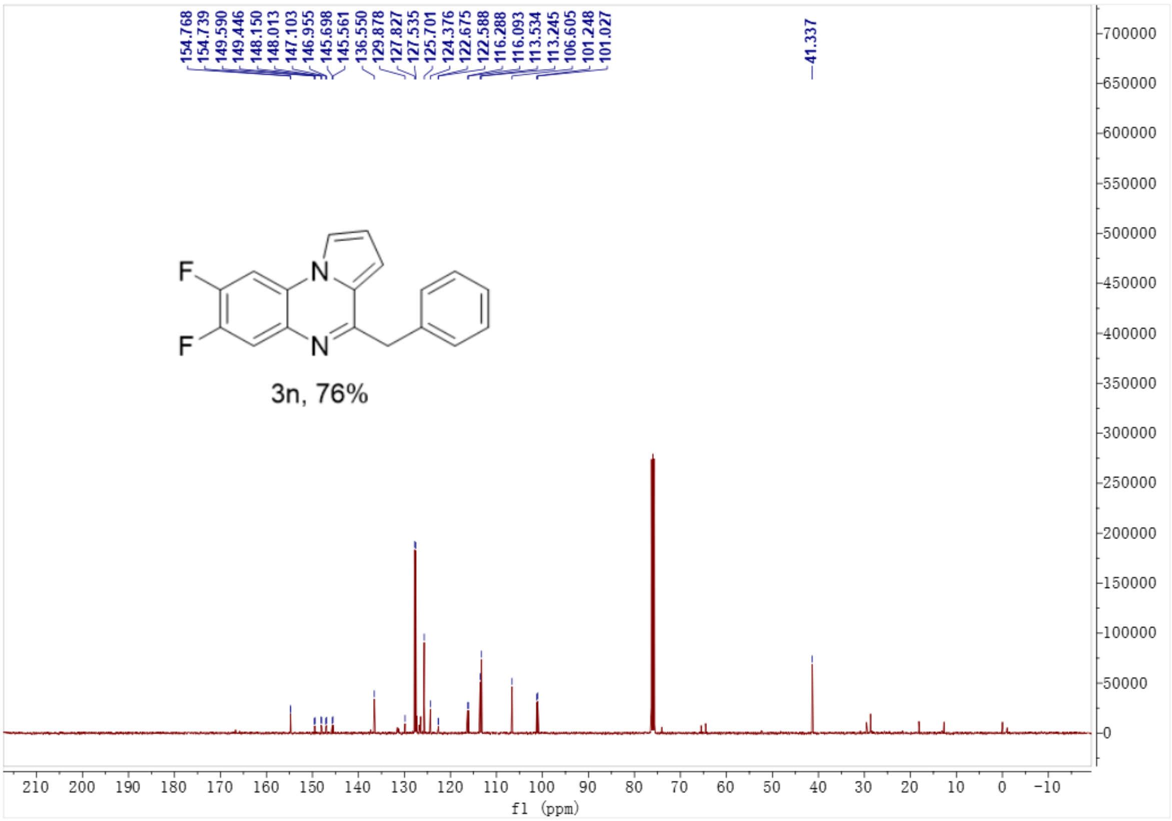

Figure S30. ^{13}C {[1] H} NMR spectrum of compound **3n** in $CDCl_3$ (100 MHz).

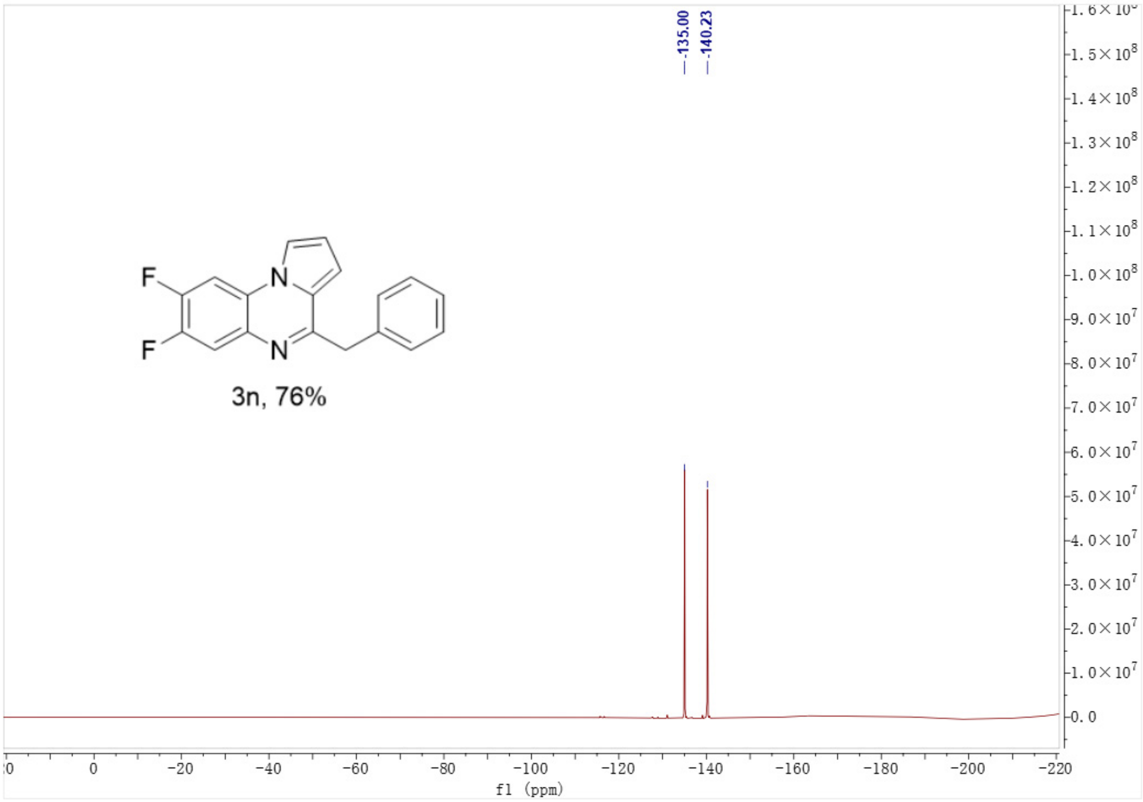

Figure S31. $^{19}F\{^{1}H\}$ NMR spectrum of compound **3n** in $CDCl_3$ (377 MHz)

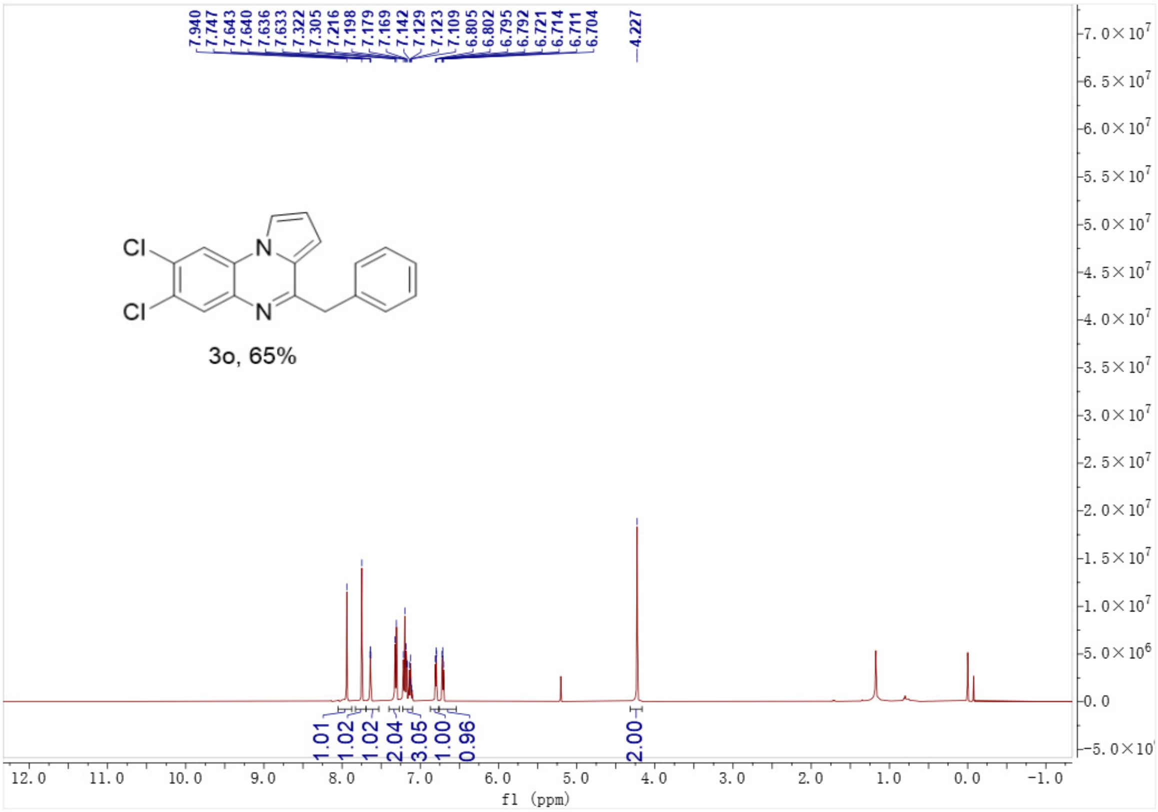

Figure S32. ^{1}H NMR spectrum of compound **3o** in $CDCl_3$ (400 MHz).

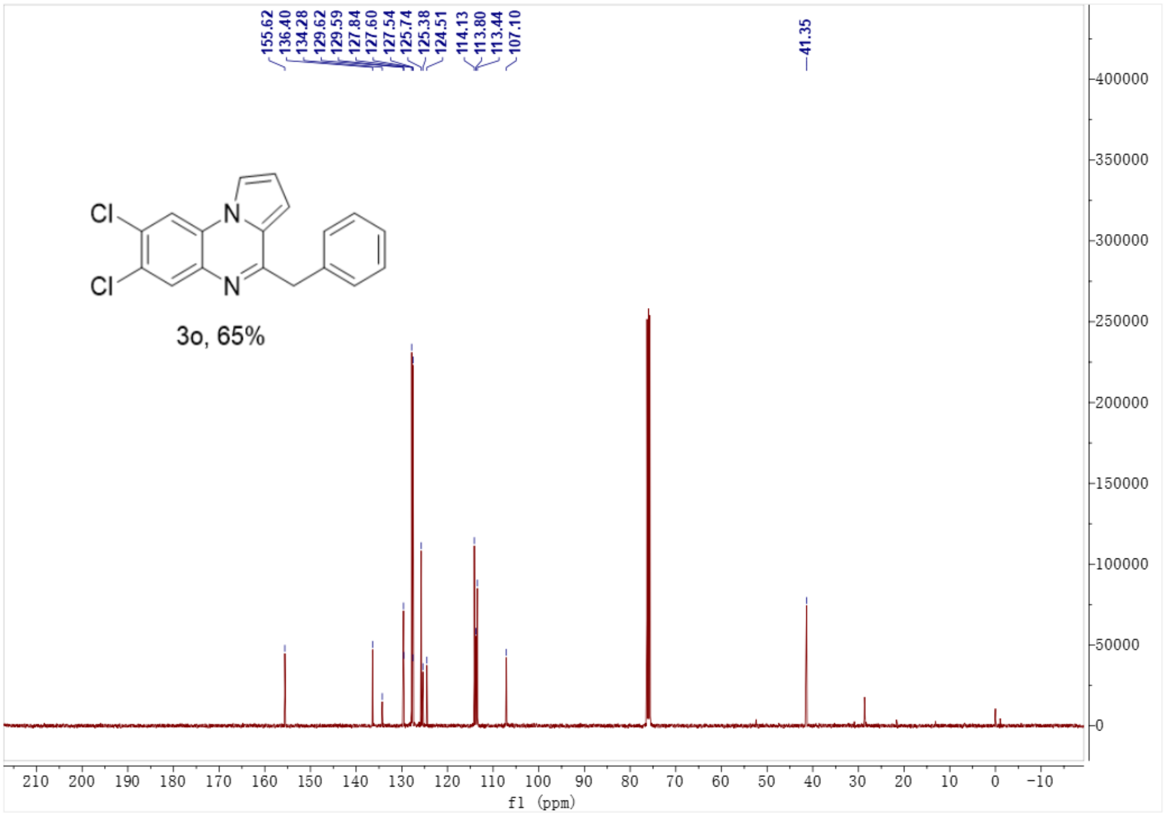

Figure S33. ^{13}C {^{1} H} NMR spectrum of compound **3o** in $CDCl_3$ (100 MHz).

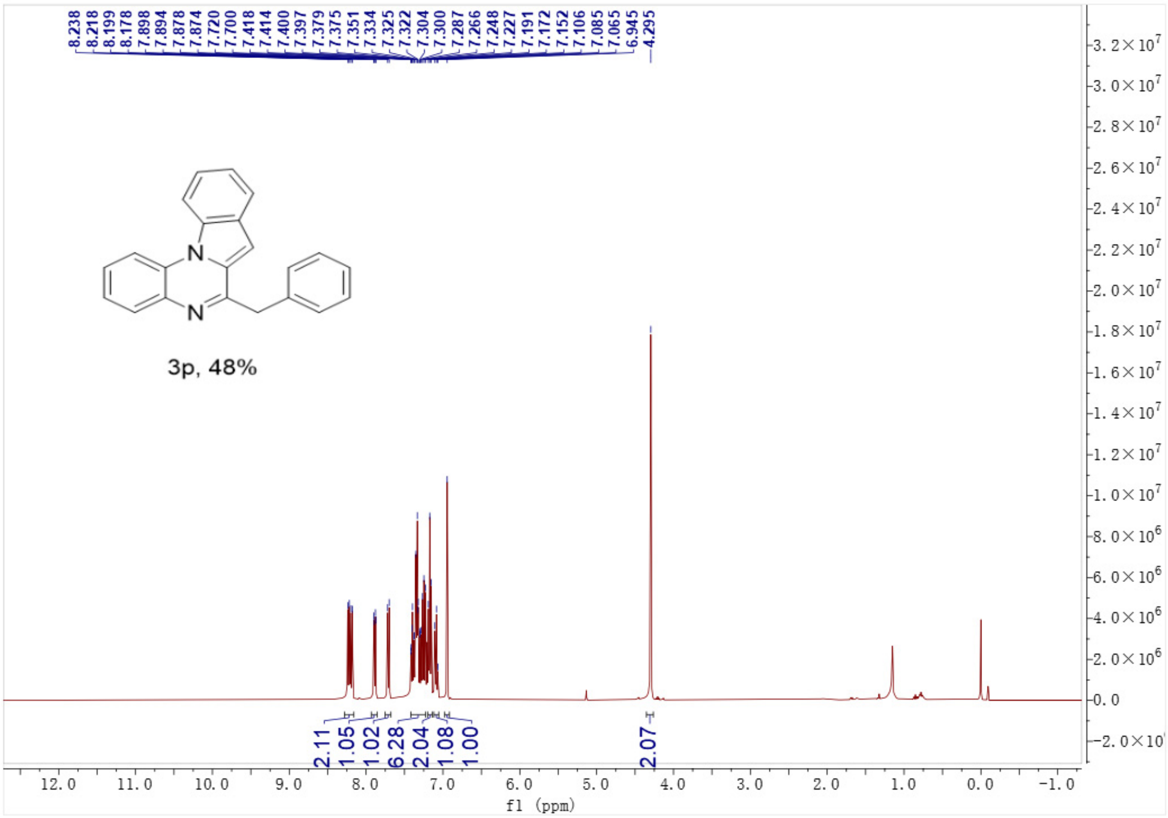

Figure S34. ^{1}H NMR spectrum of compound **3p** in $CDCl_3$ (400 MHz).

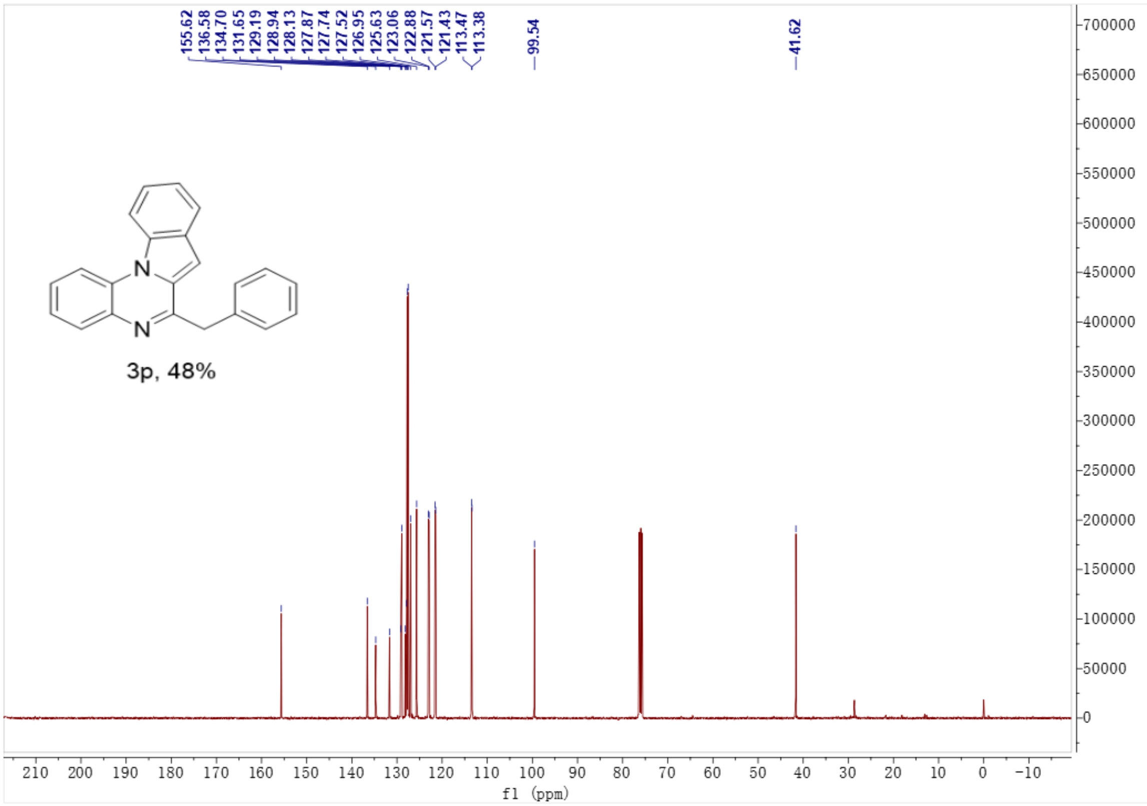

Figure S35. ^{13}C {^{1} H} NMR spectrum of compound **3p** in $CDCl_3$ (100 MHz).

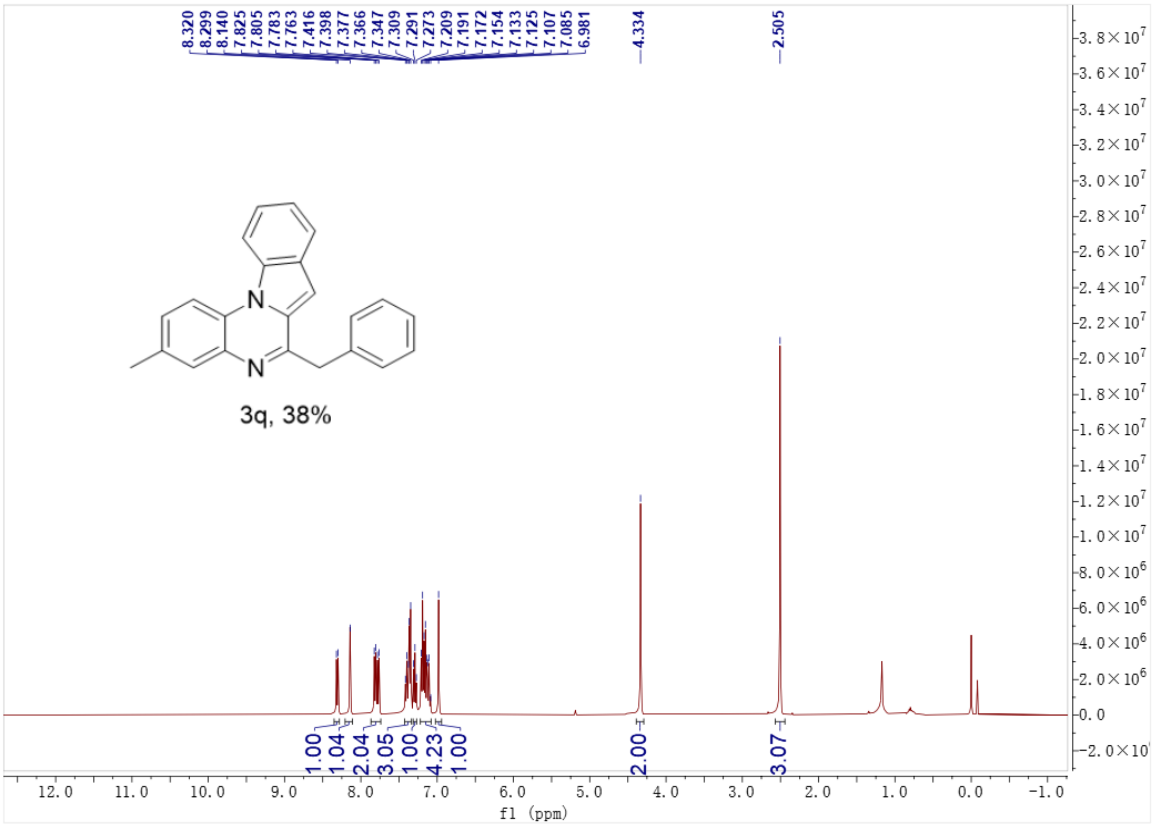

Figure S36. ^{1}H NMR spectrum of compound **3q** in $CDCl_3$ (400 MHz).

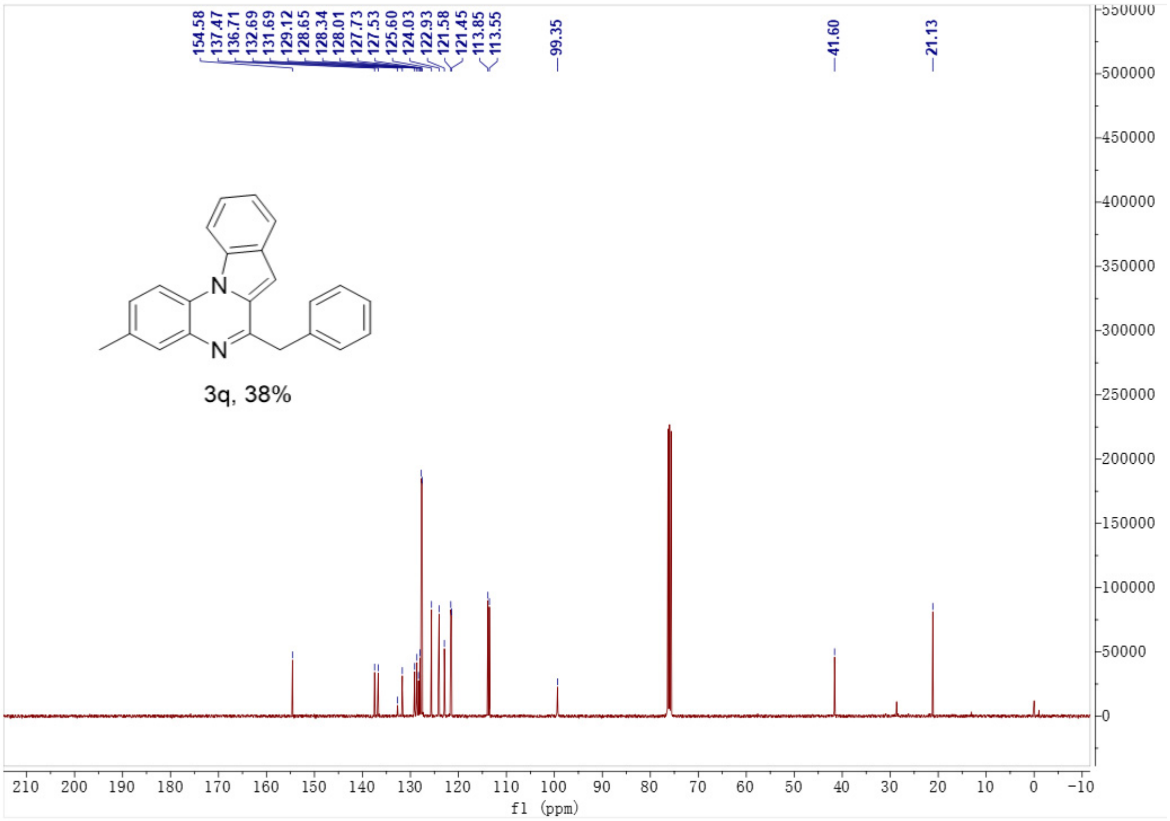

Figure S37. $^{13}C\{^1H\}$ NMR spectrum of compound **3q** in $CDCl_3$ (100 MHz).

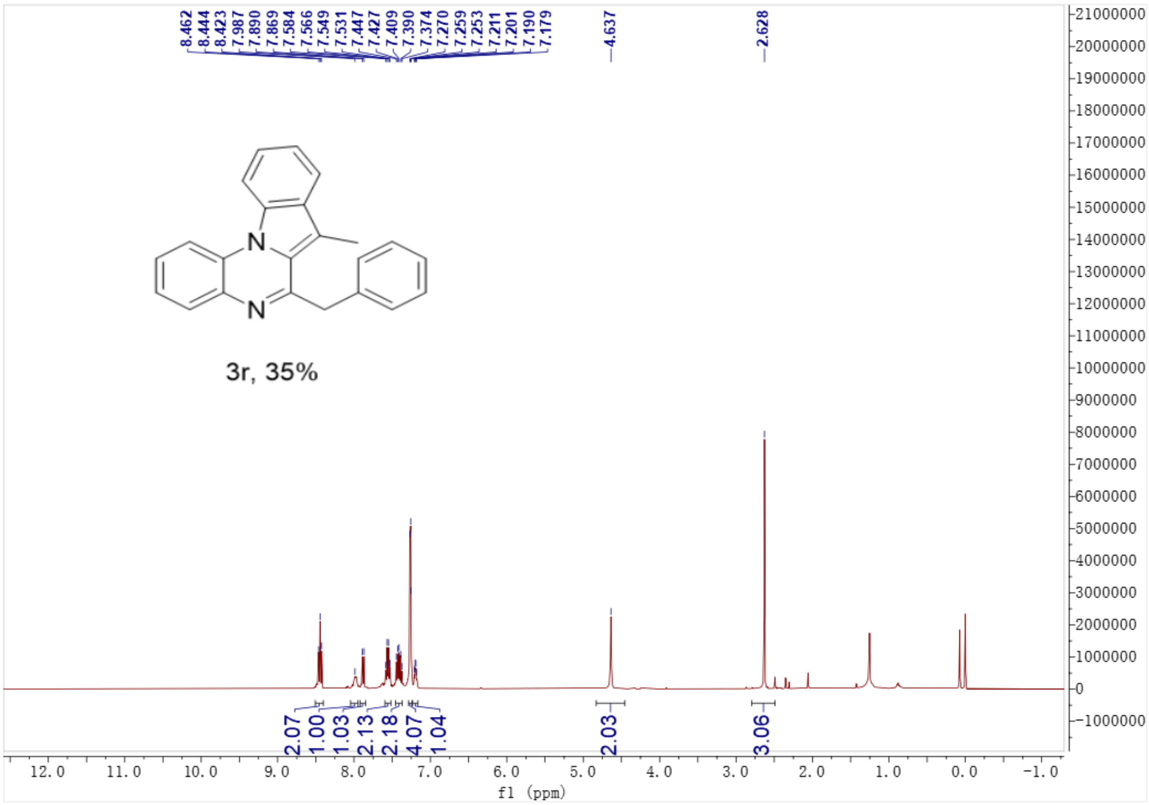

Figure S38. ^1H NMR spectrum of compound **3r** in $CDCl_3$ (400 MHz).

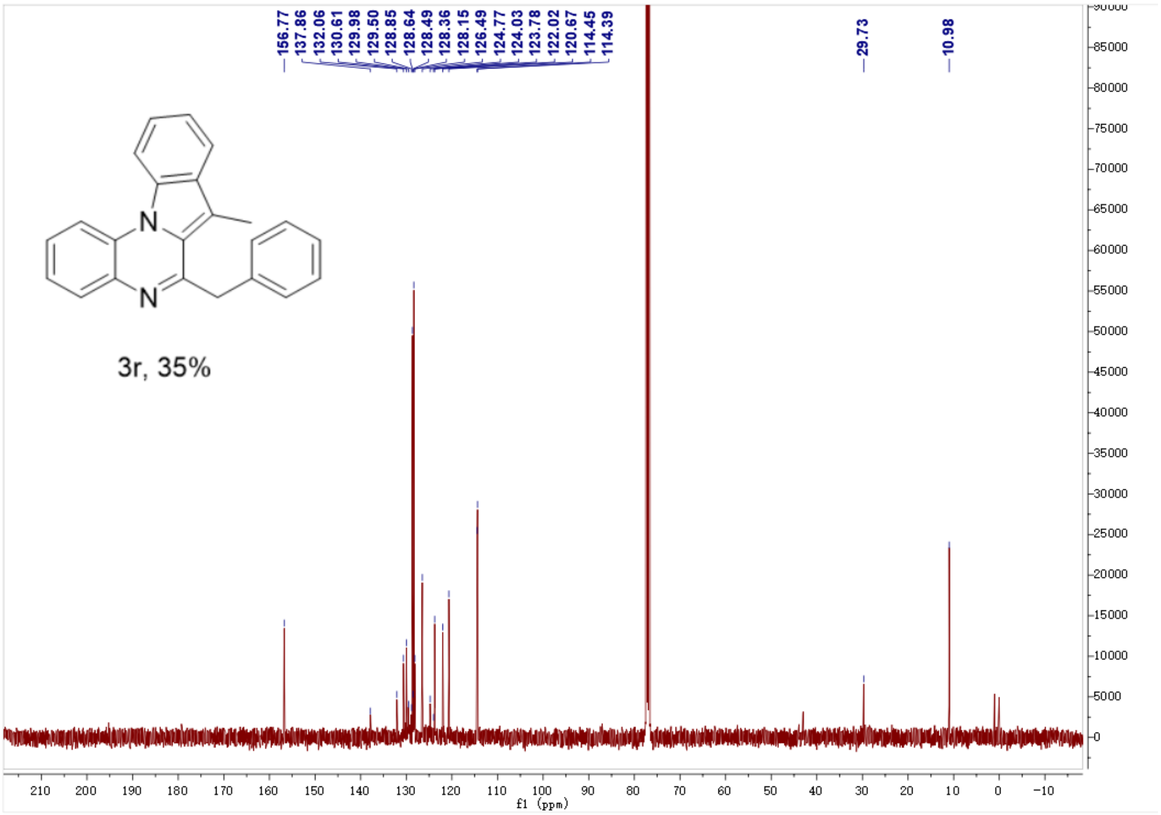

Figure S39. ^{13}C {^{1} H} NMR spectrum of compound **3r** in $CDCl_3$ (100 MHz).

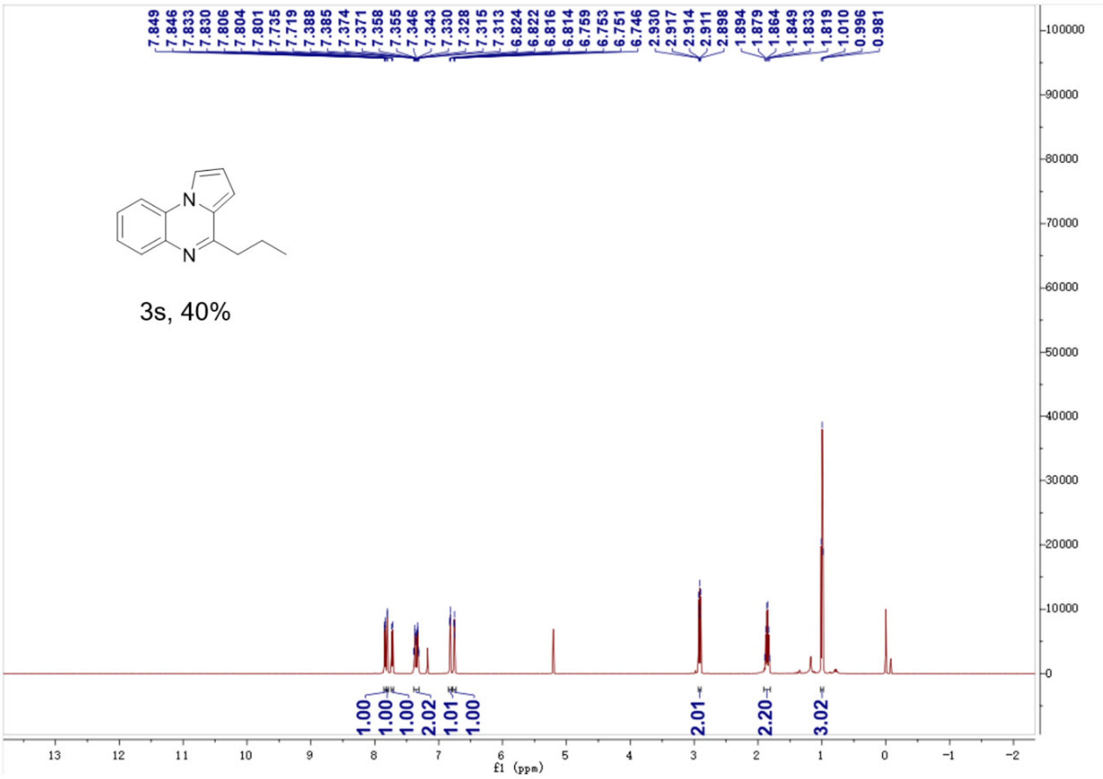

Figure S40. ^{1}H NMR spectrum of compound **3s** in $CDCl_3$ (500 MHz).

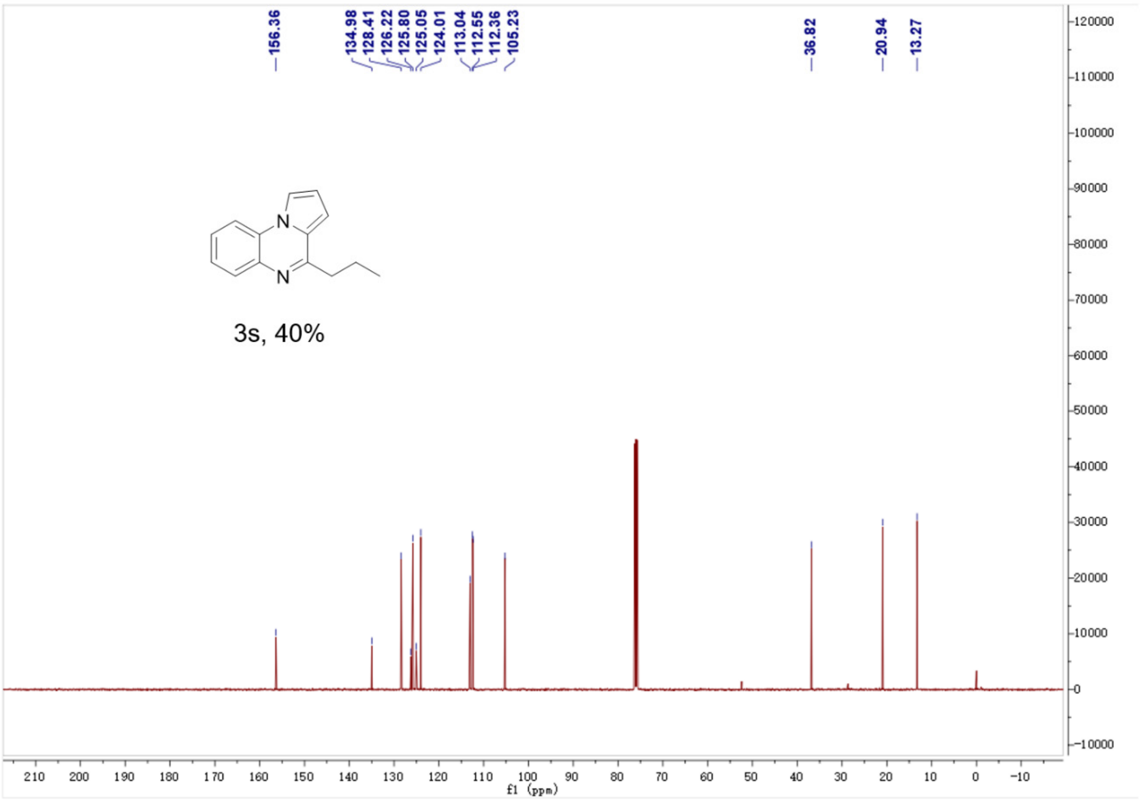

Figure S41. $^{13}C\{^{1}H\}$ NMR spectrum of compound **3s** in $CDCl_3$ (125 MHz).

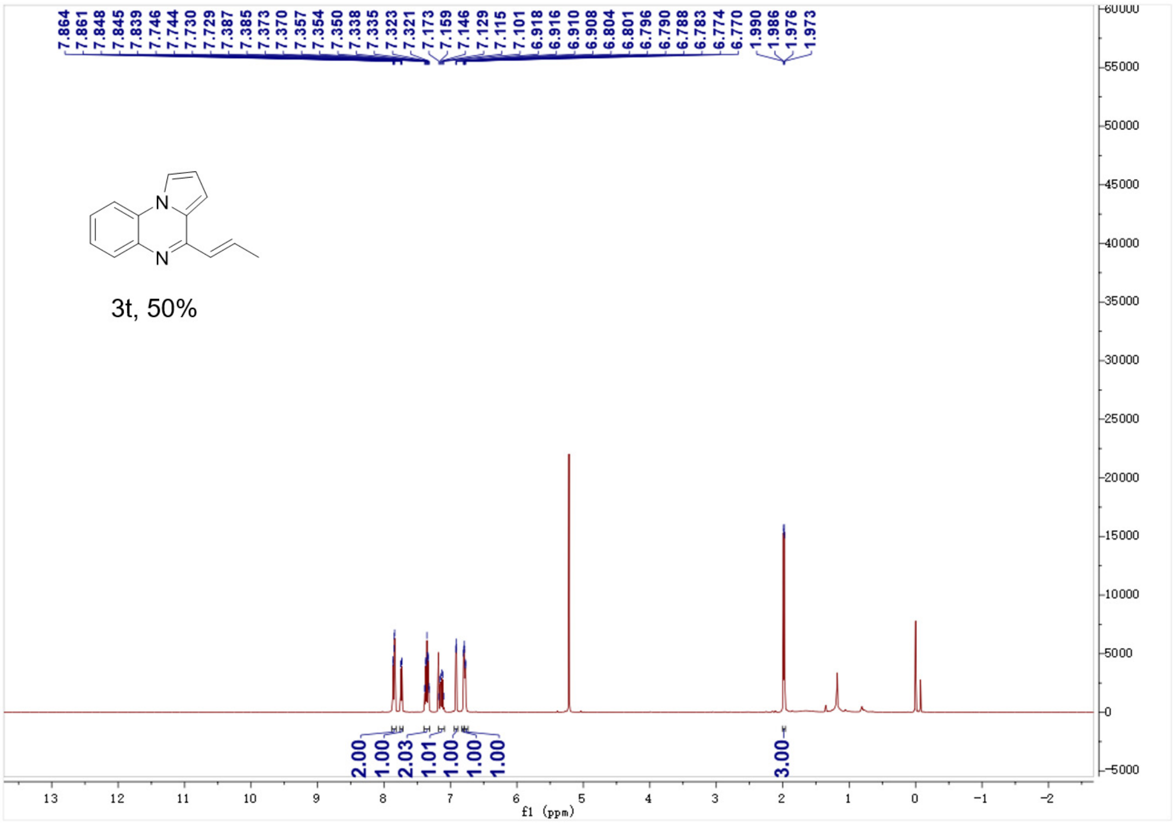

Figure S42. ^{1}H NMR spectrum of compound **3t** in $CDCl_3$ (500 MHz).

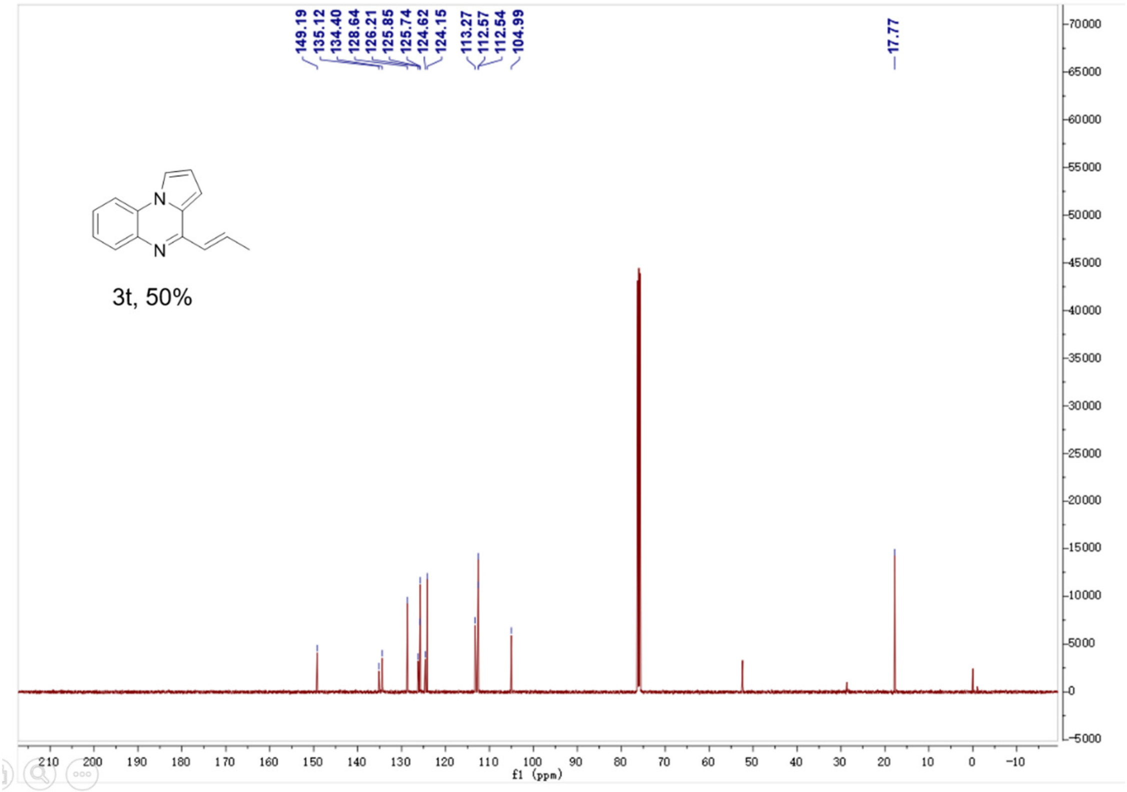

Figure S43. $^{13}C\{^{1}H\}$ NMR spectrum of compound **3t** in $CDCl_3$ (125 MHz).

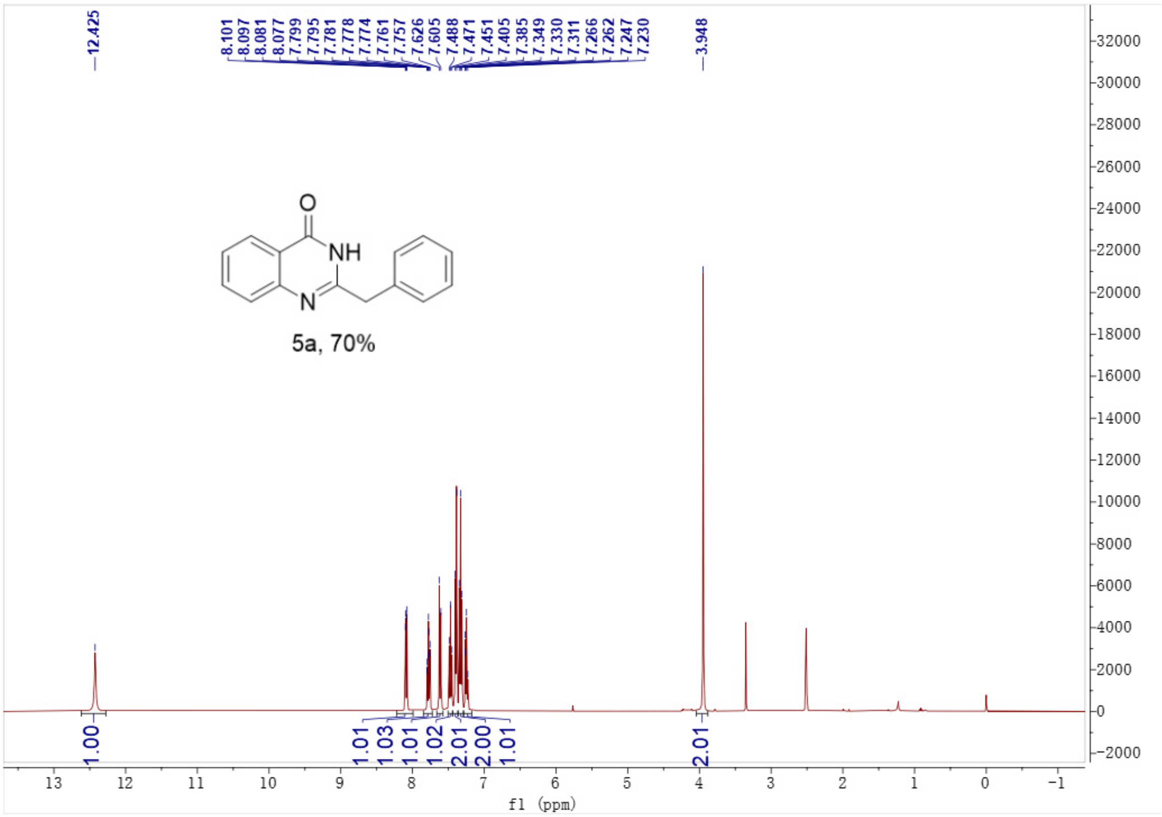

Figure S44. ^{1}H NMR spectrum of compound **5a** in DMSO-d_6 (400 MHz).

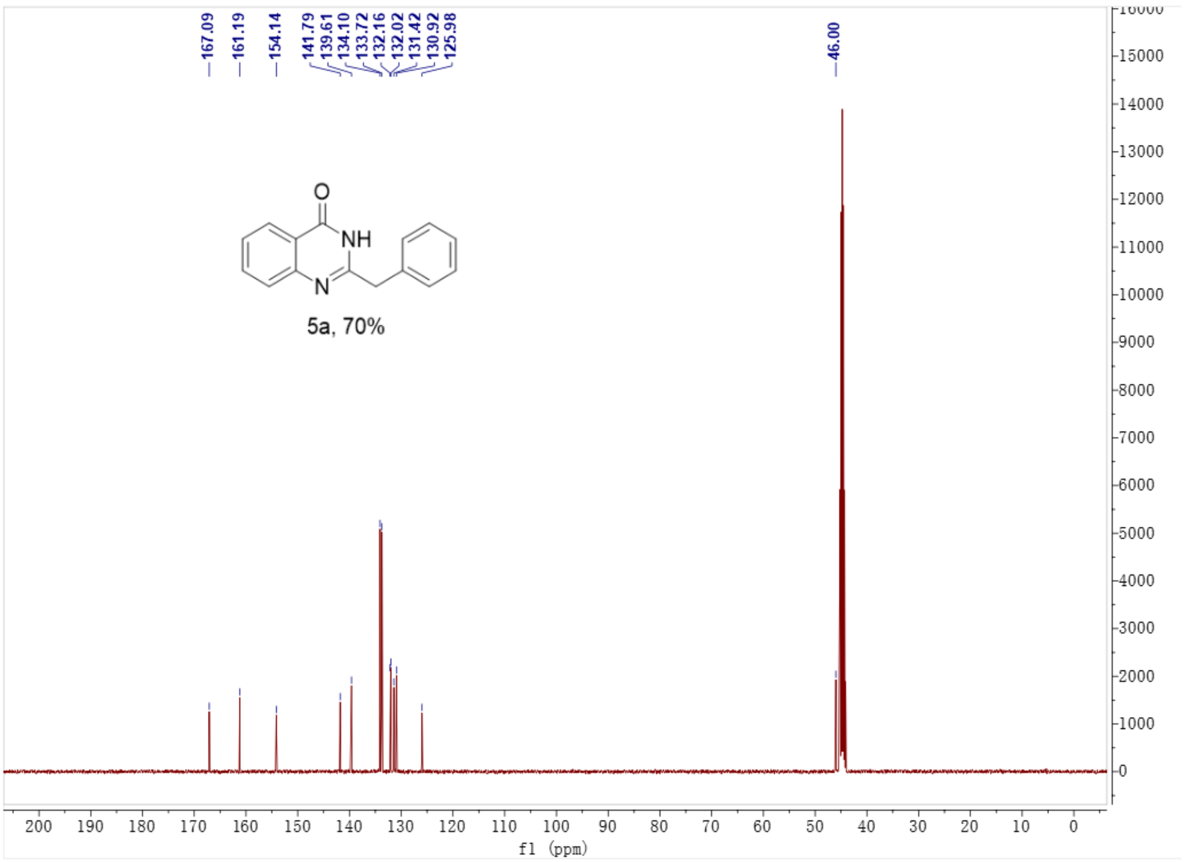

Figure S45. ^{13}C {^{1} H} NMR spectrum of compound **5a** in DMSO-d_6 (100 MHz).

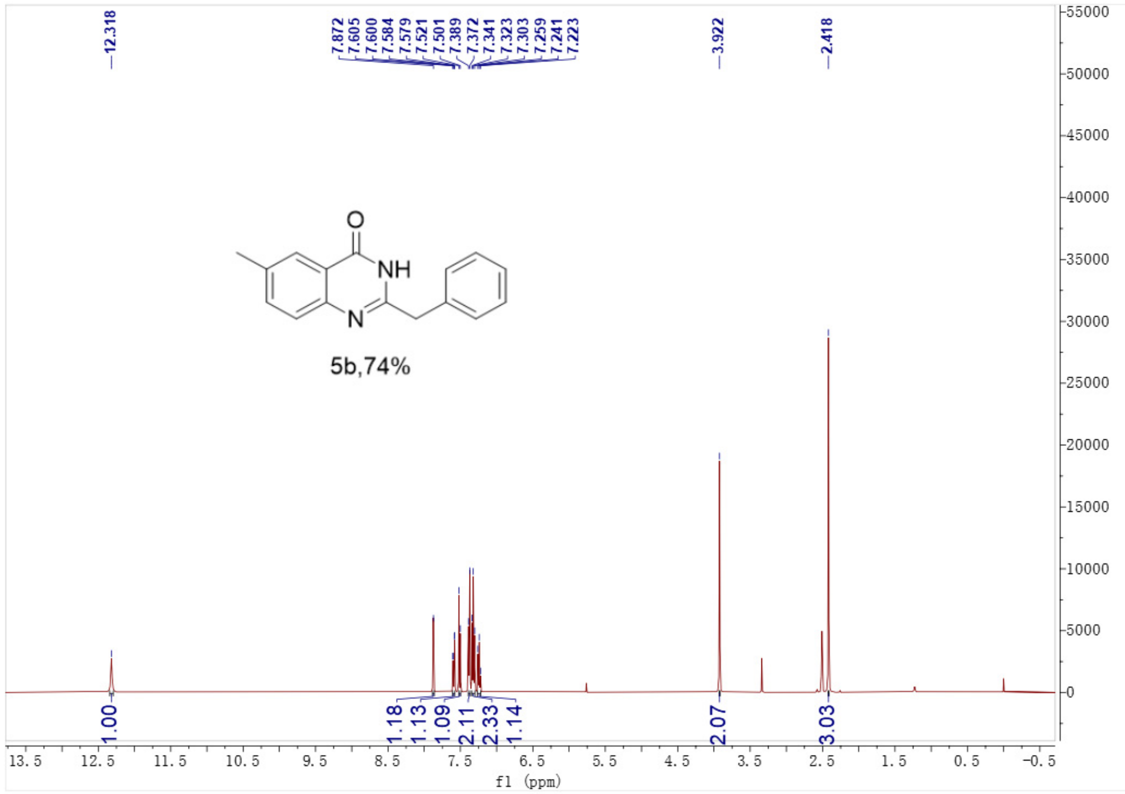

Figure S46. ^{1}H NMR spectrum of compound **5b** in DMSO-d_6 (400 MHz).

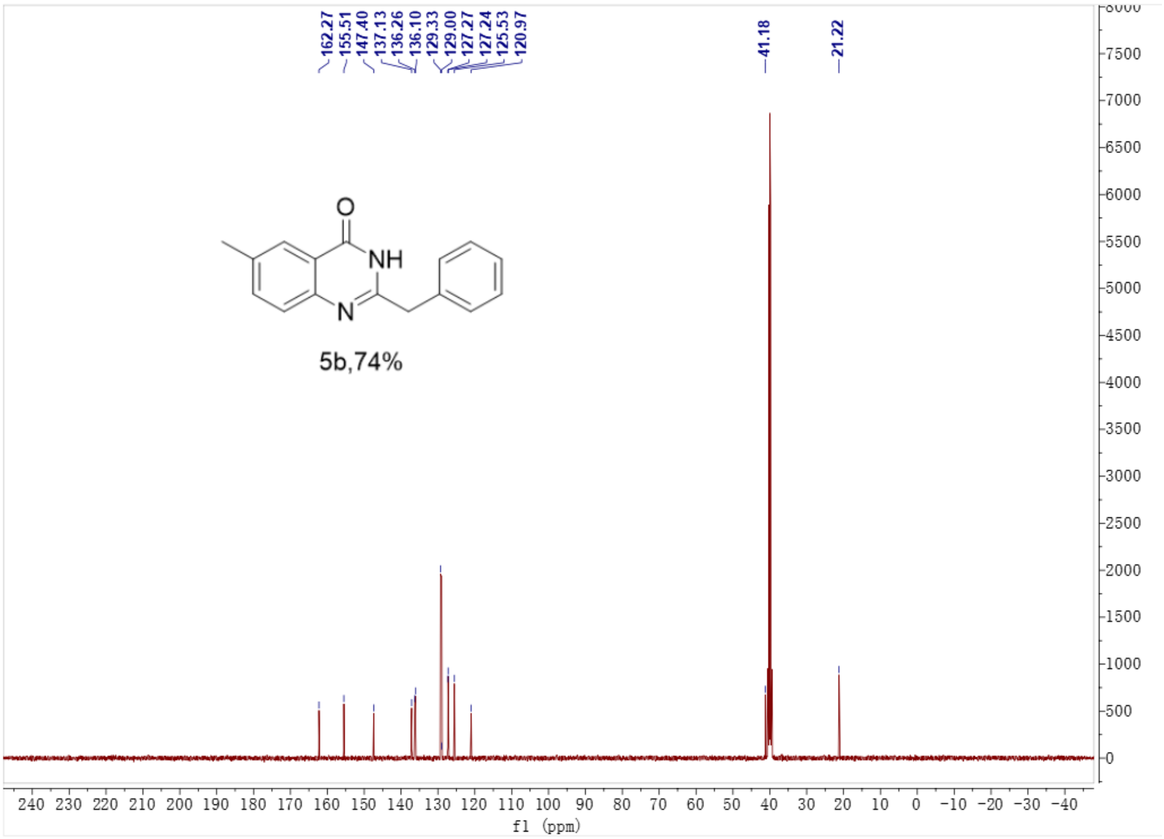

Figure S47. ^{13}C {^{1} H} NMR spectrum of compound **5b** in DMSO-*d*6 (100 MHz).

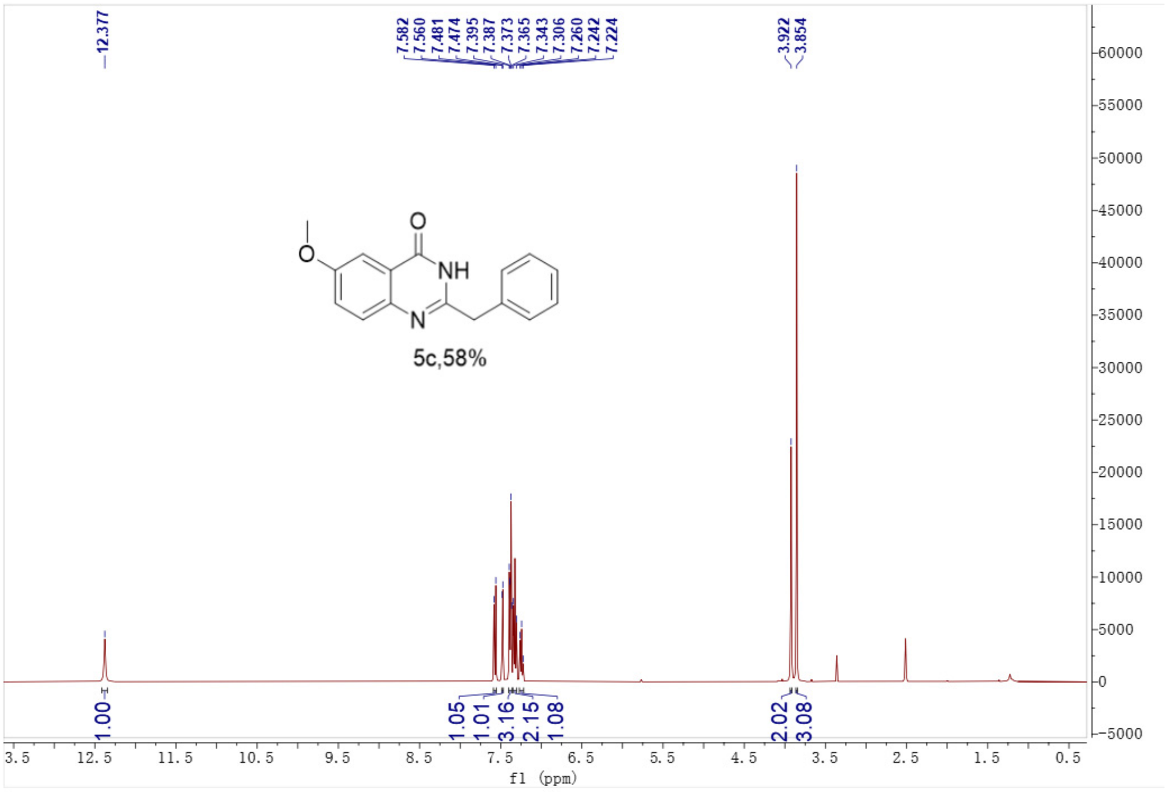

Figure S48. ^{1}H NMR spectrum of compound **5c** in DMSO-*d*6 (400 MHz).

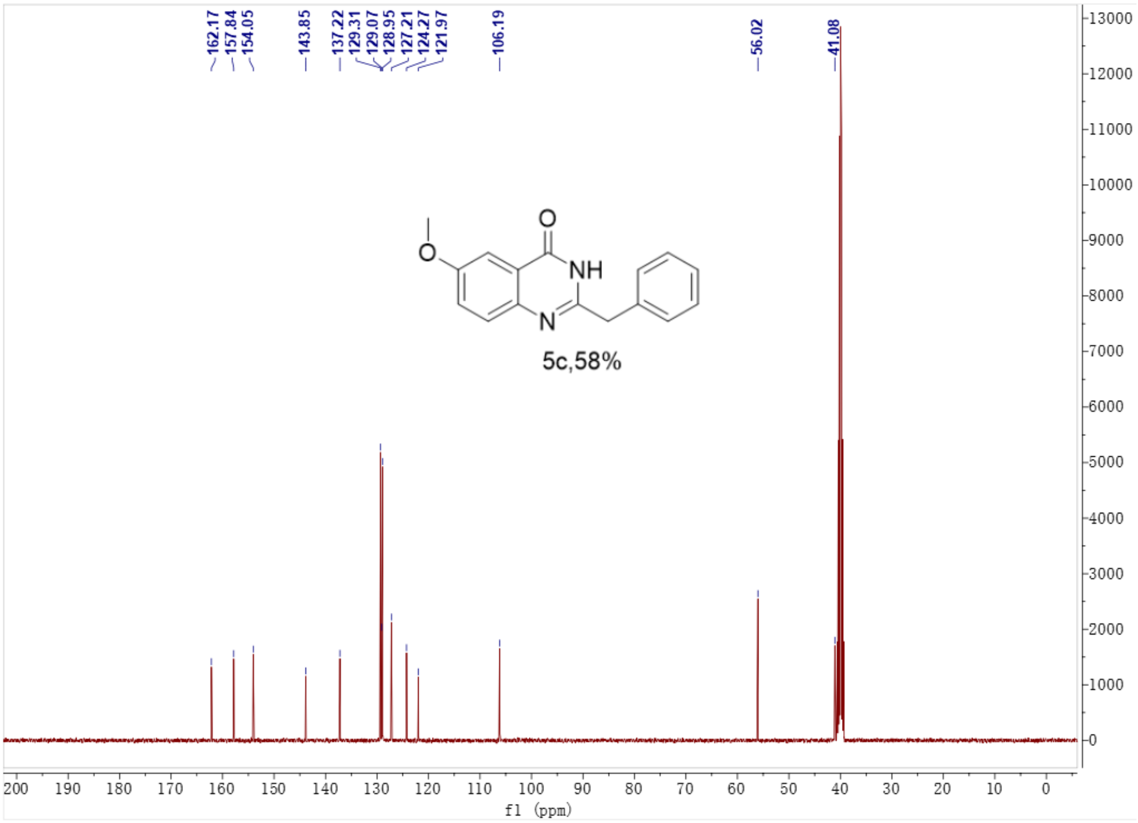

Figure S49. ^{13}C {[1] H} NMR spectrum of compound **5c** in DMSO-d_6 (100 MHz).

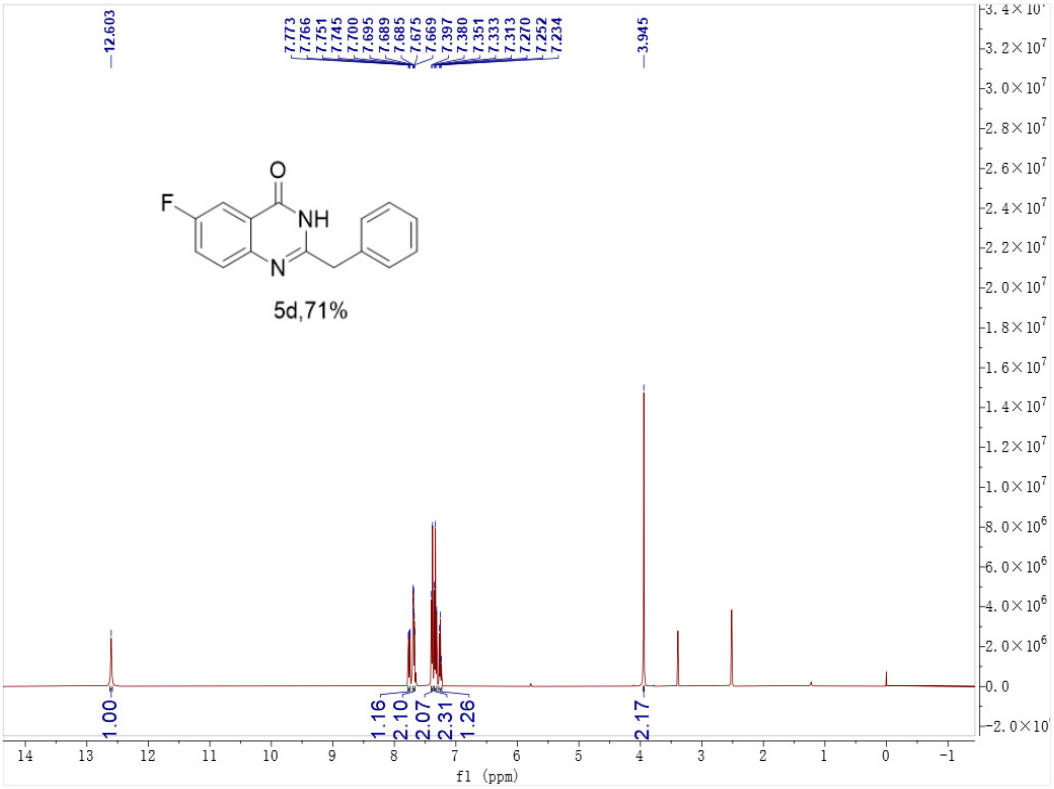

Figure S50. ^{1}H NMR spectrum of compound **5d** in DMSO-d_6 (400 MHz).

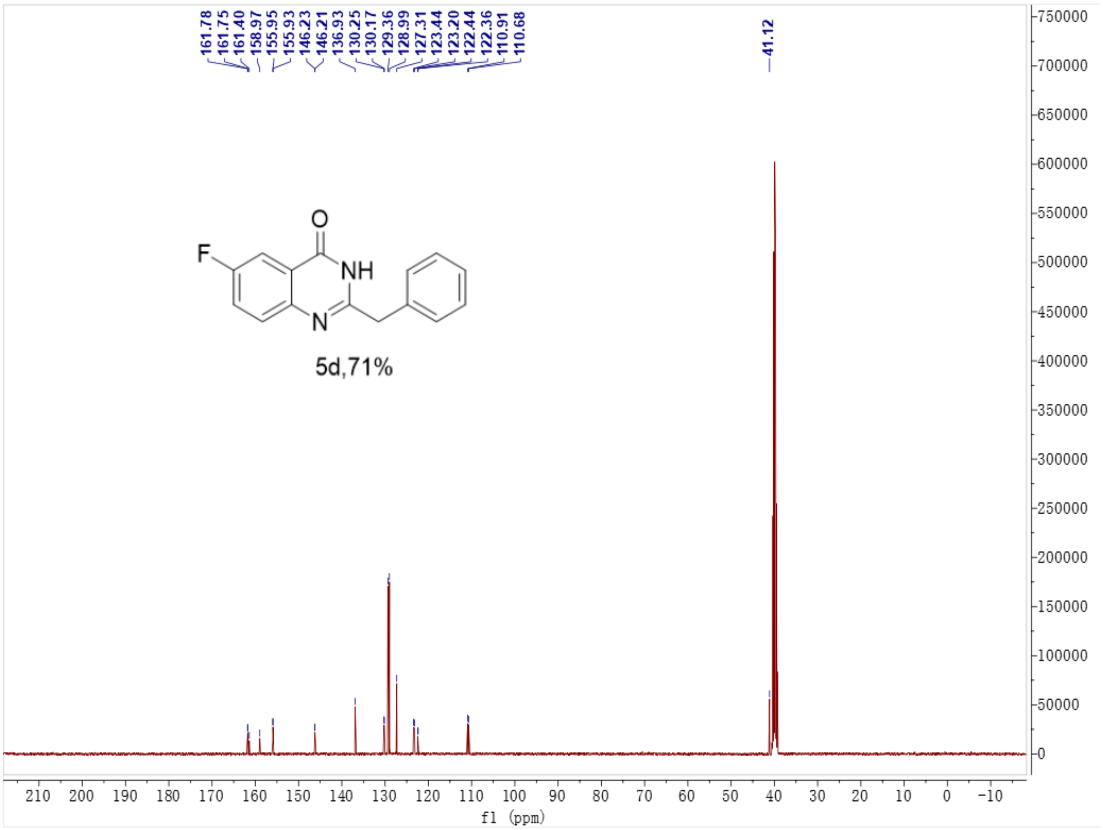

Figure S51. ^{13}C {^{1} H} NMR spectrum of compound **5d** in DMSO-d_6 (100 MHz).

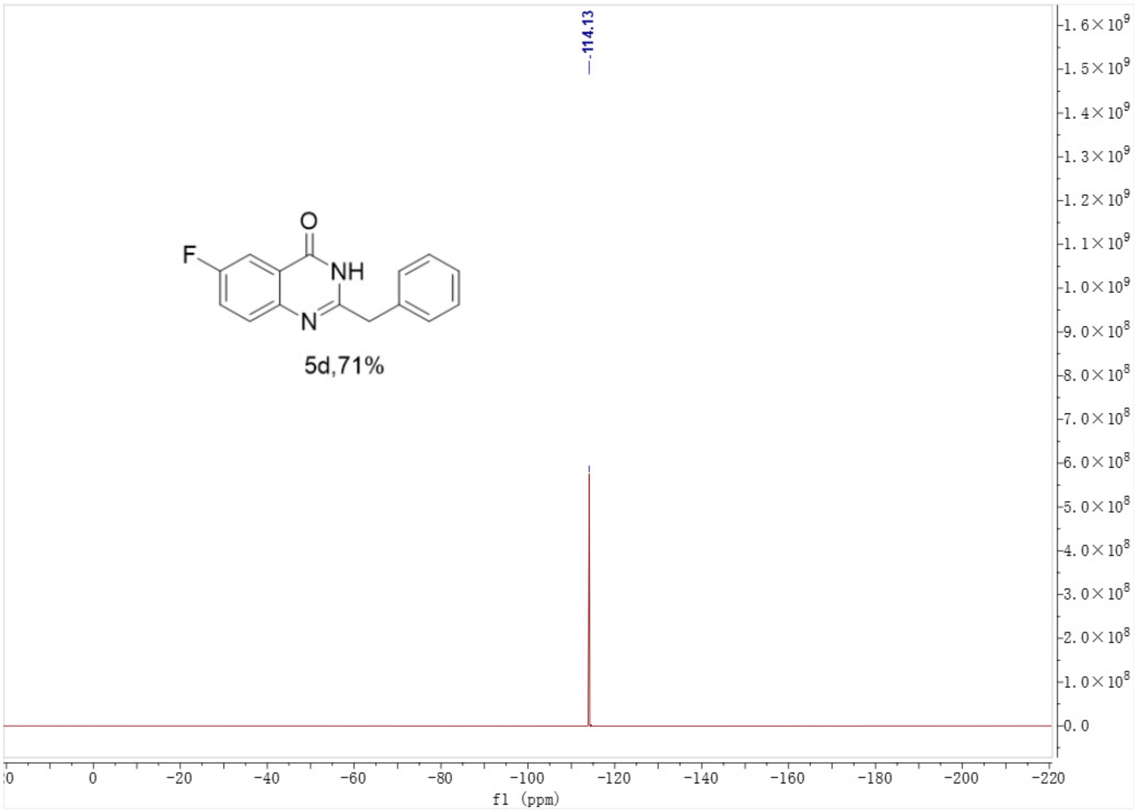

Figure S52. ^{19}F {^{1} H} NMR spectrum of compound **5d** in DMSO-d_6 (377 MHz).

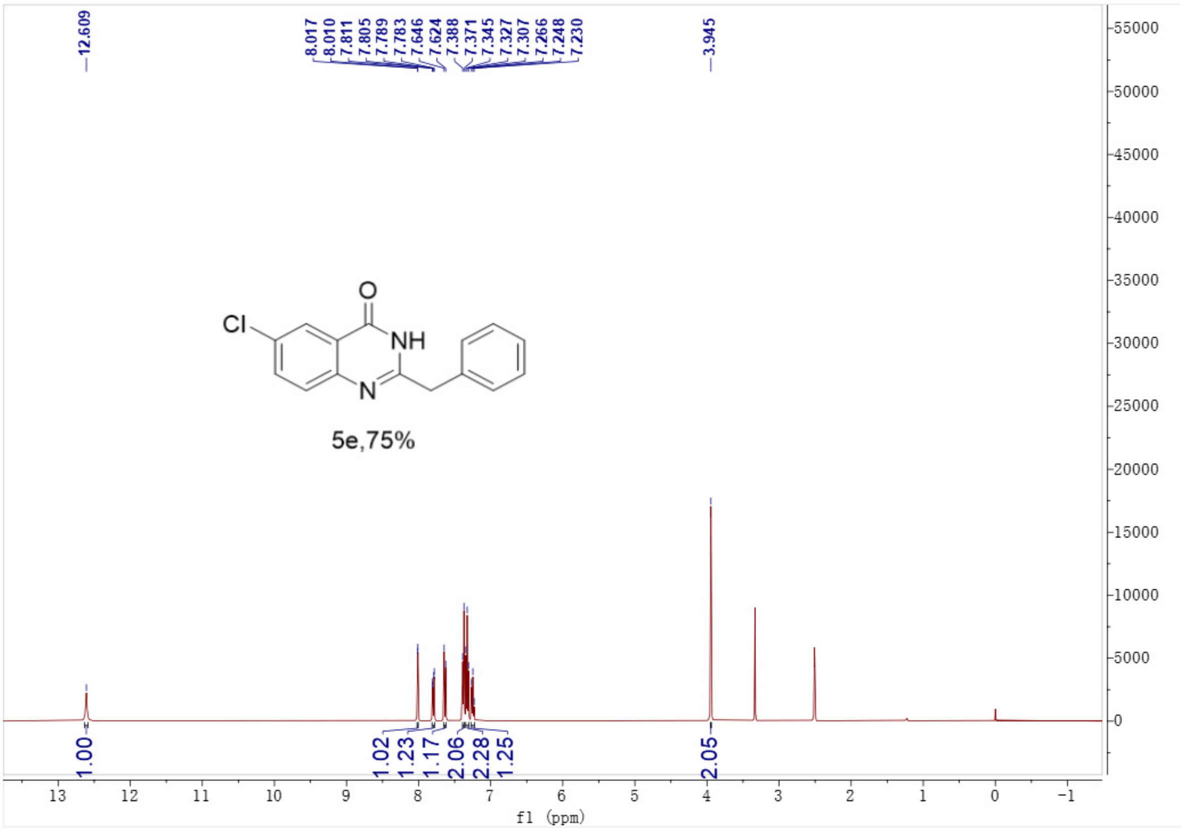

Figure S53. ^{1}H NMR spectrum of compound **5e** in DMSO-d_6 (400 MHz).

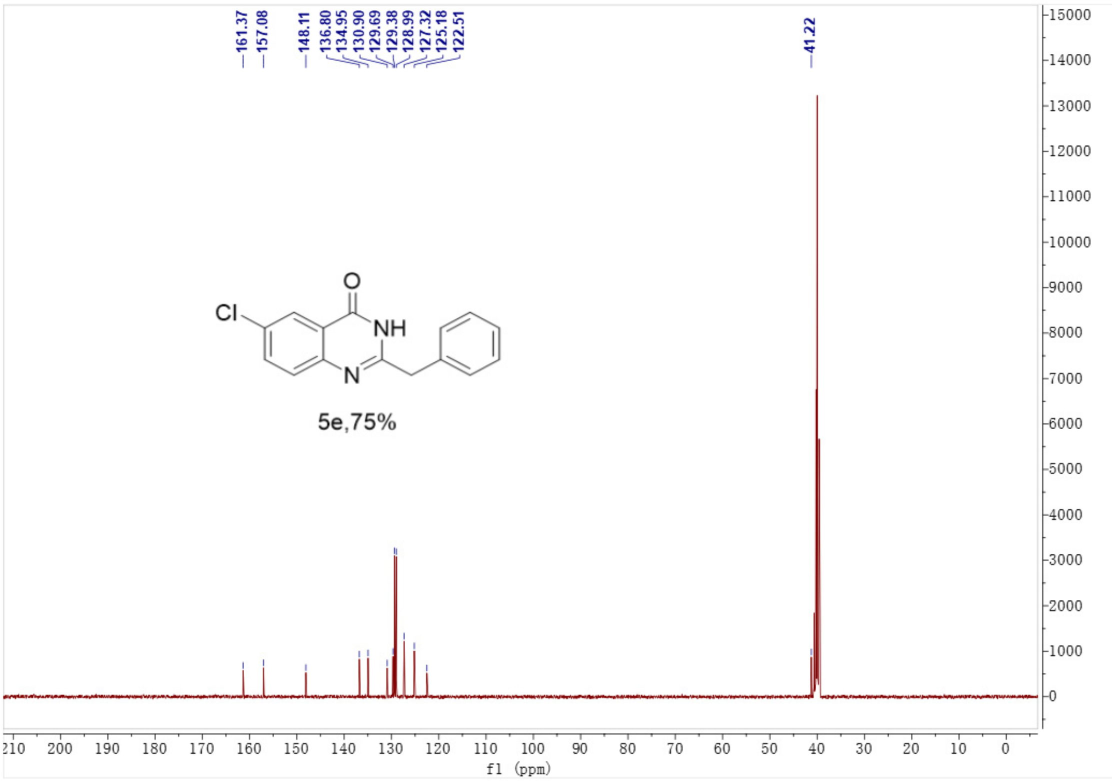

Figure S54. ^{13}C {^{1} H} NMR spectrum of compound **5e** in DMSO-d_6 (100 MHz).

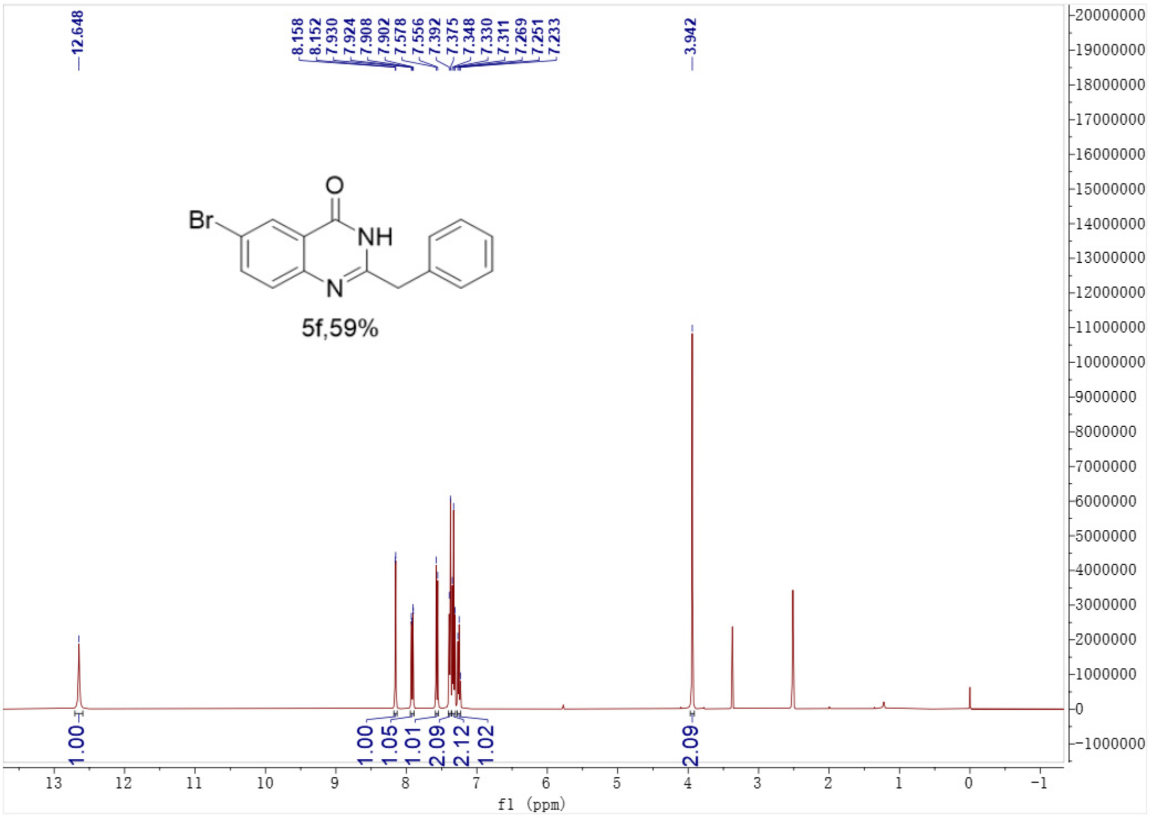

Figure S55. ^{1}H NMR spectrum of compound **5f** in DMSO-d_6 (400 MHz).

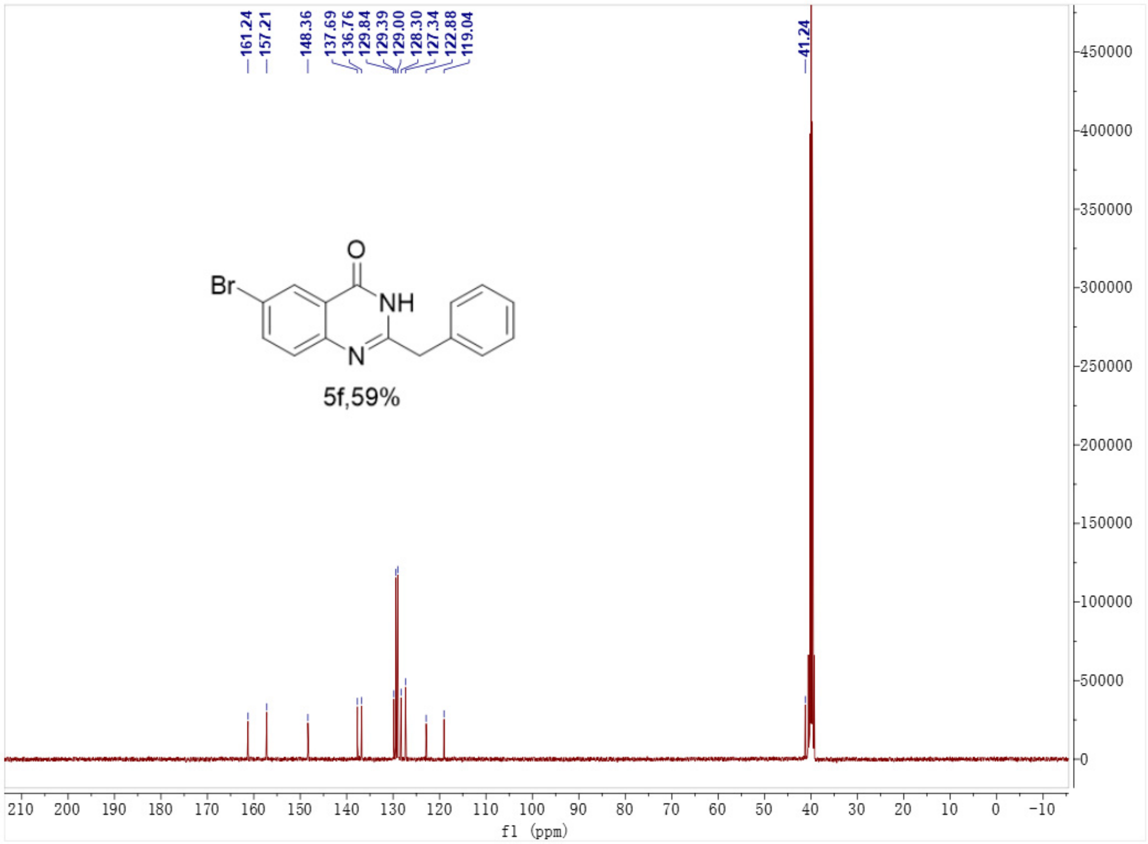

Figure S56. ^{13}C {^{1} H} NMR spectrum of compound **5f** in DMSO-d_6 (100 MHz).

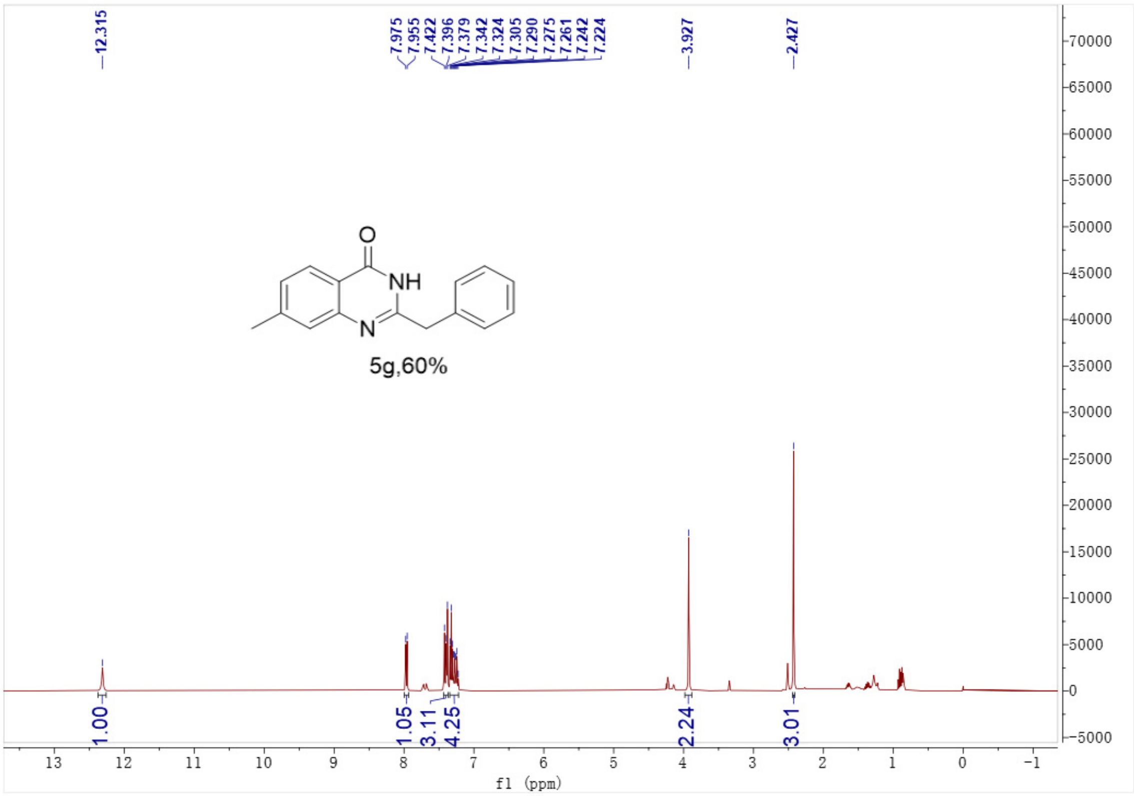

Figure S57. ^{1}H NMR spectrum of compound **5g** in DMSO-*d*6 (400 MHz).

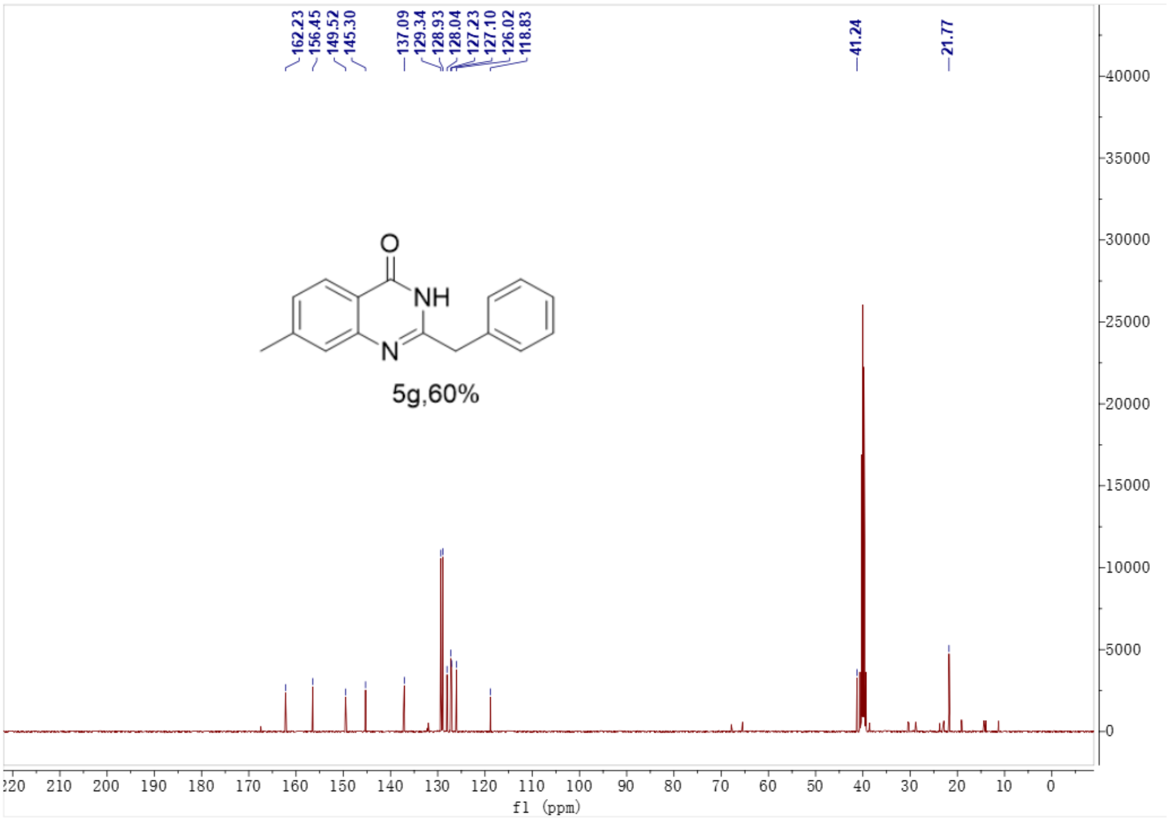

Figure S58. ^{13}C {[1] H} NMR spectrum of compound **5g** in DMSO-*d*6 (100 MHz).

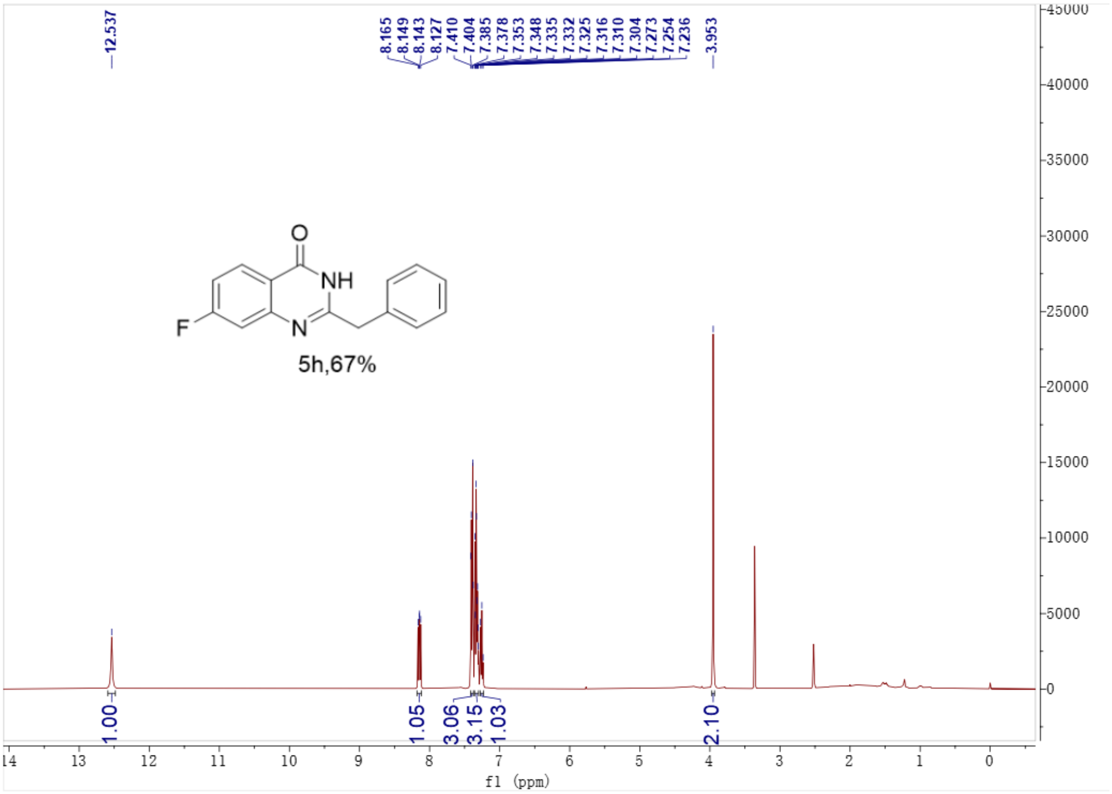

Figure S59. ^{1}H NMR spectrum of compound **5h** in DMSO-*d*6 (400 MHz).

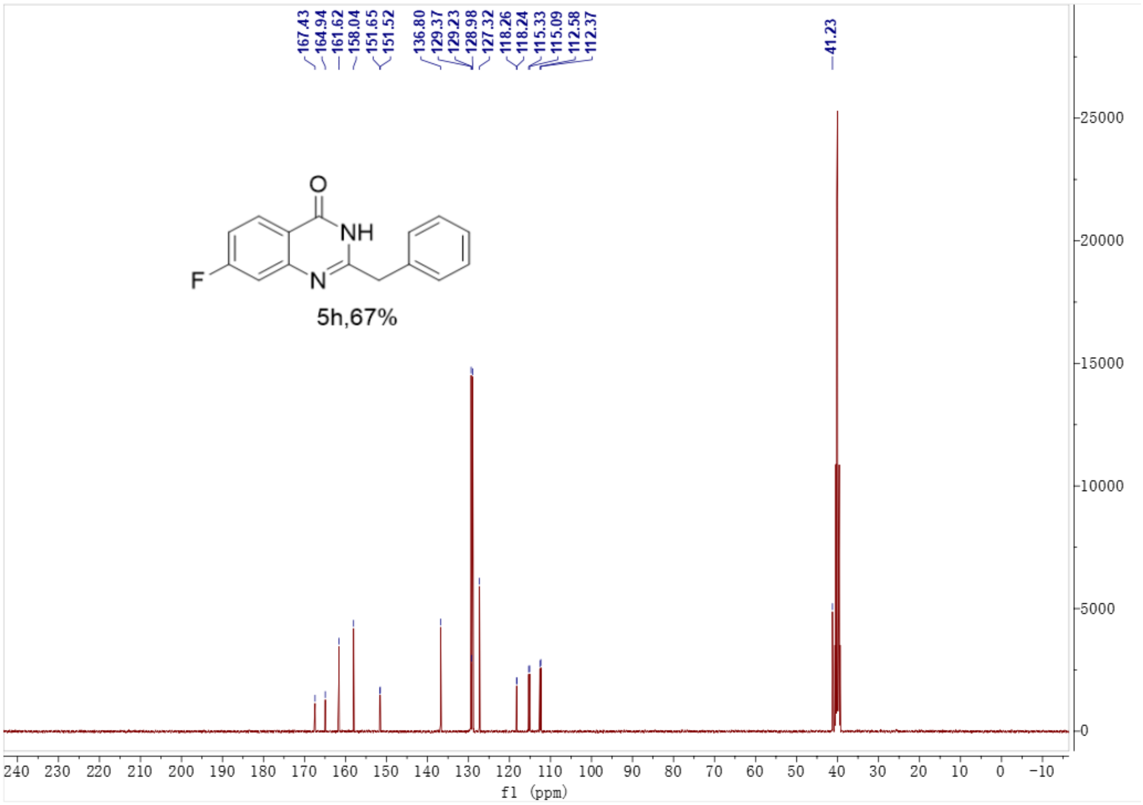

Figure S60. ^{13}C {[1] H} NMR spectrum of compound **5h** in DMSO-*d*6 (100 MHz).

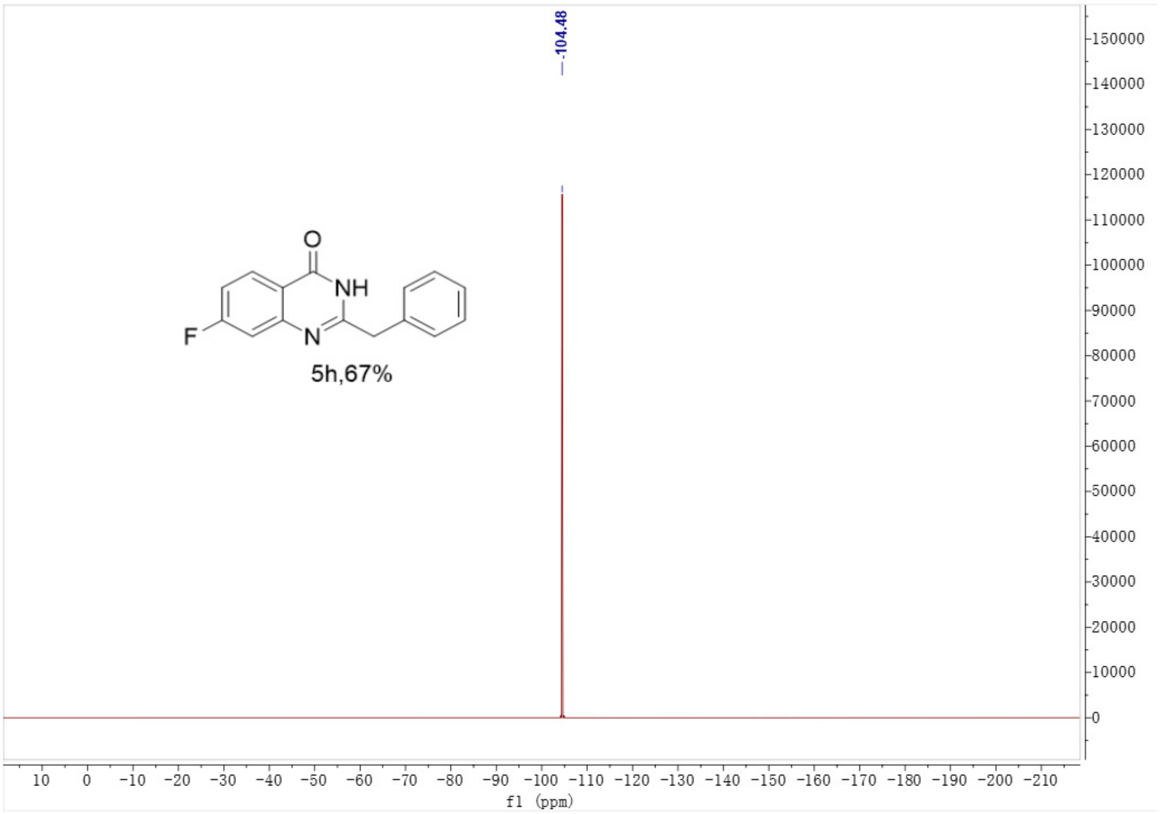

Figure S61. ^{19}F {[1] H} NMR spectrum of compound **5h** in DMSO-d_6 (377 MHz).

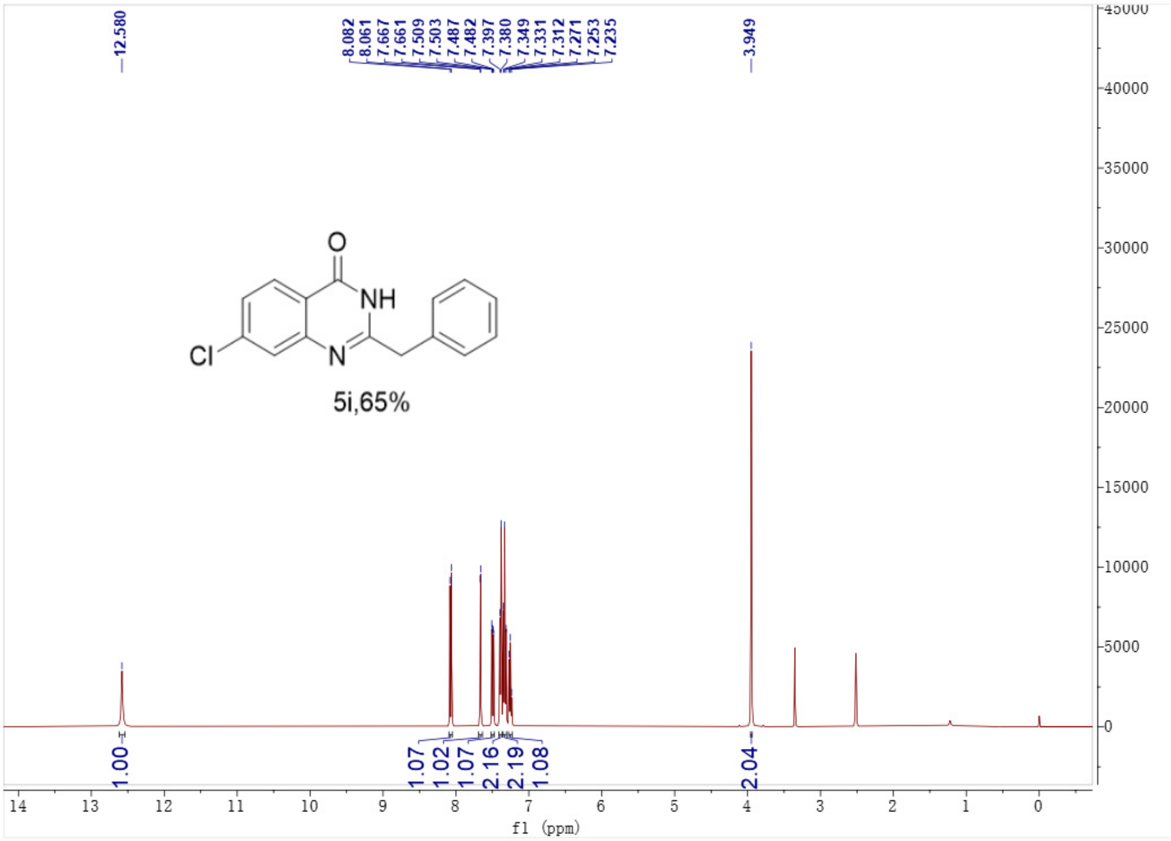

Figure S62. ^{1}H NMR spectrum of compound **5i** in DMSO-d_6 (400 MHz).

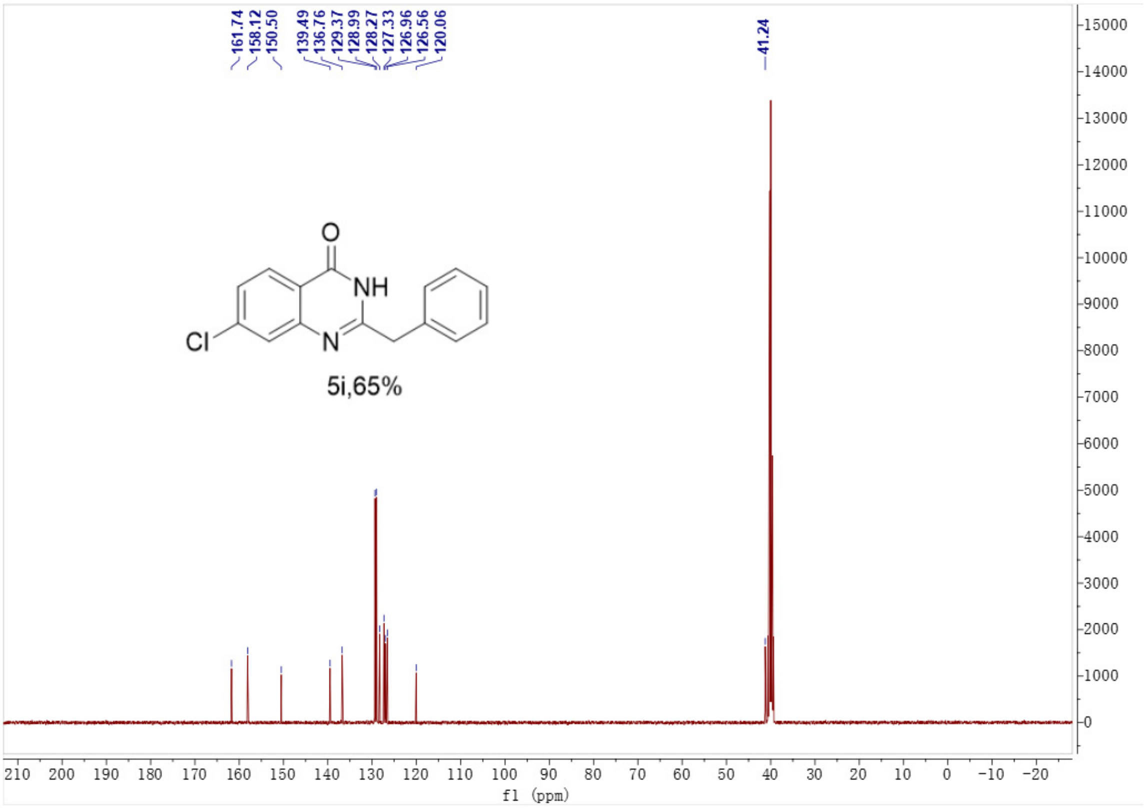

Figure S63. ^{13}C {^{1} H} NMR spectrum of compound **5i** in DMSO-d_6 (100 MHz).

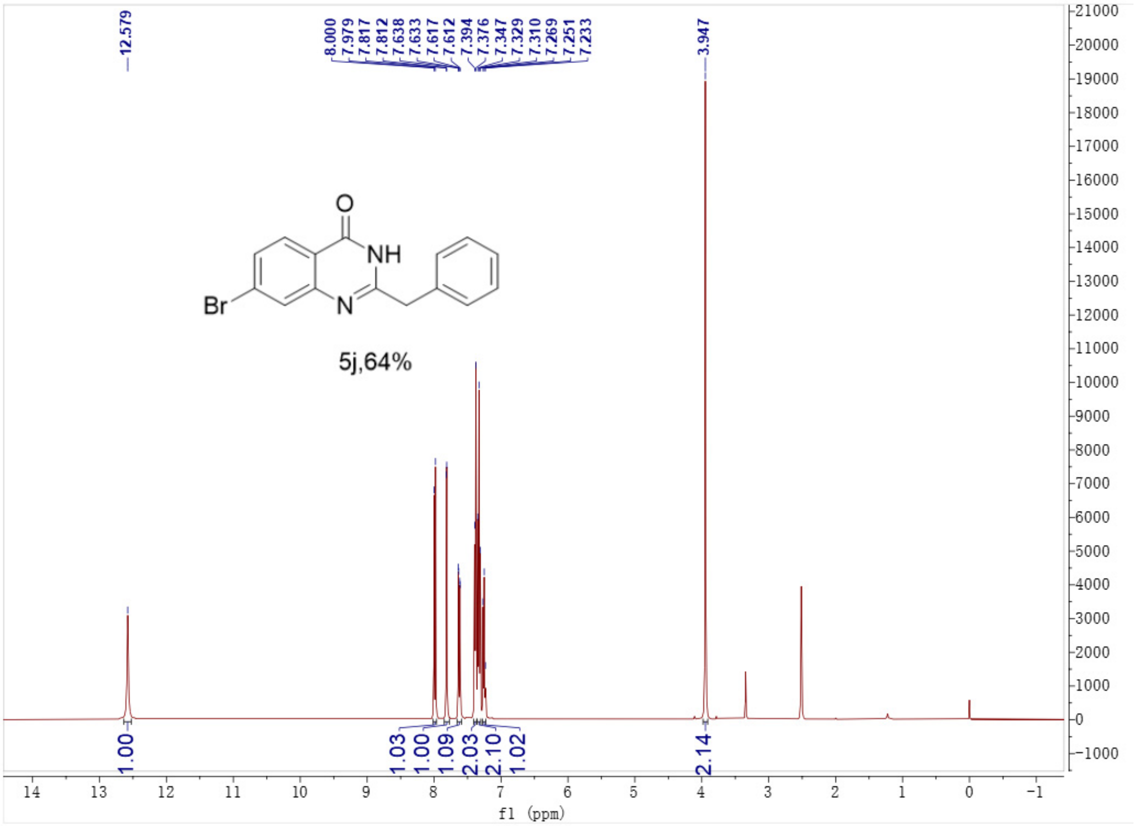

Figure S64. ^{1}H NMR spectrum of compound **5j** in DMSO-d_6 (400 MHz).

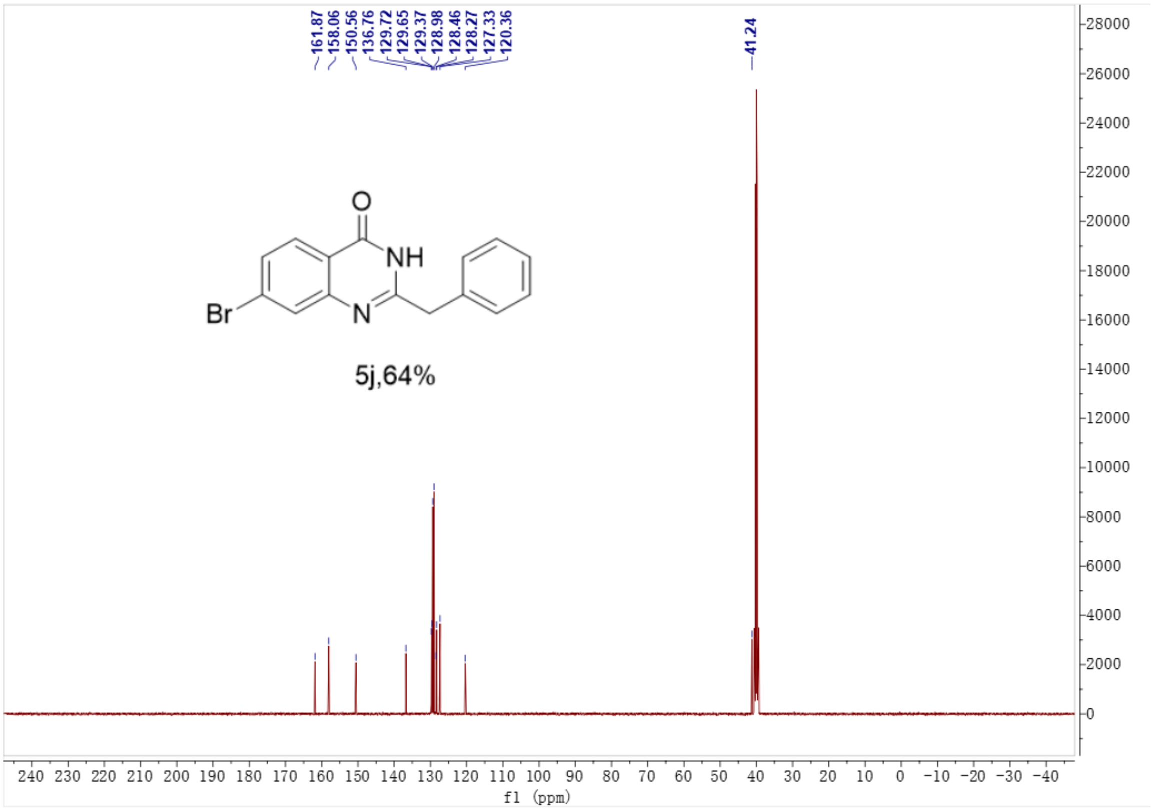

Figure S65. $^{13}C\ \{^{1}H\}$ NMR spectrum of compound **5j** in DMSO-d_6 (100 MHz).

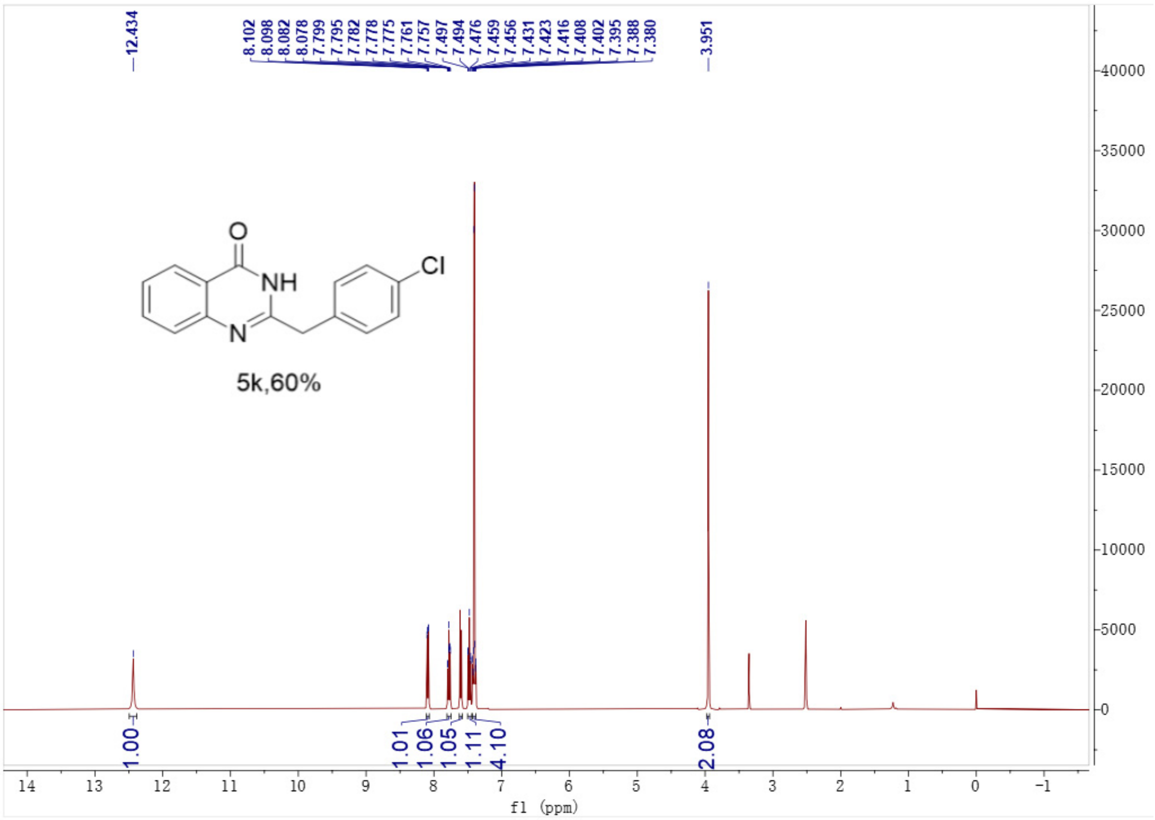

Figure S66. ^{1}H NMR spectrum of compound **5k** in DMSO-d_6 (400 MHz).

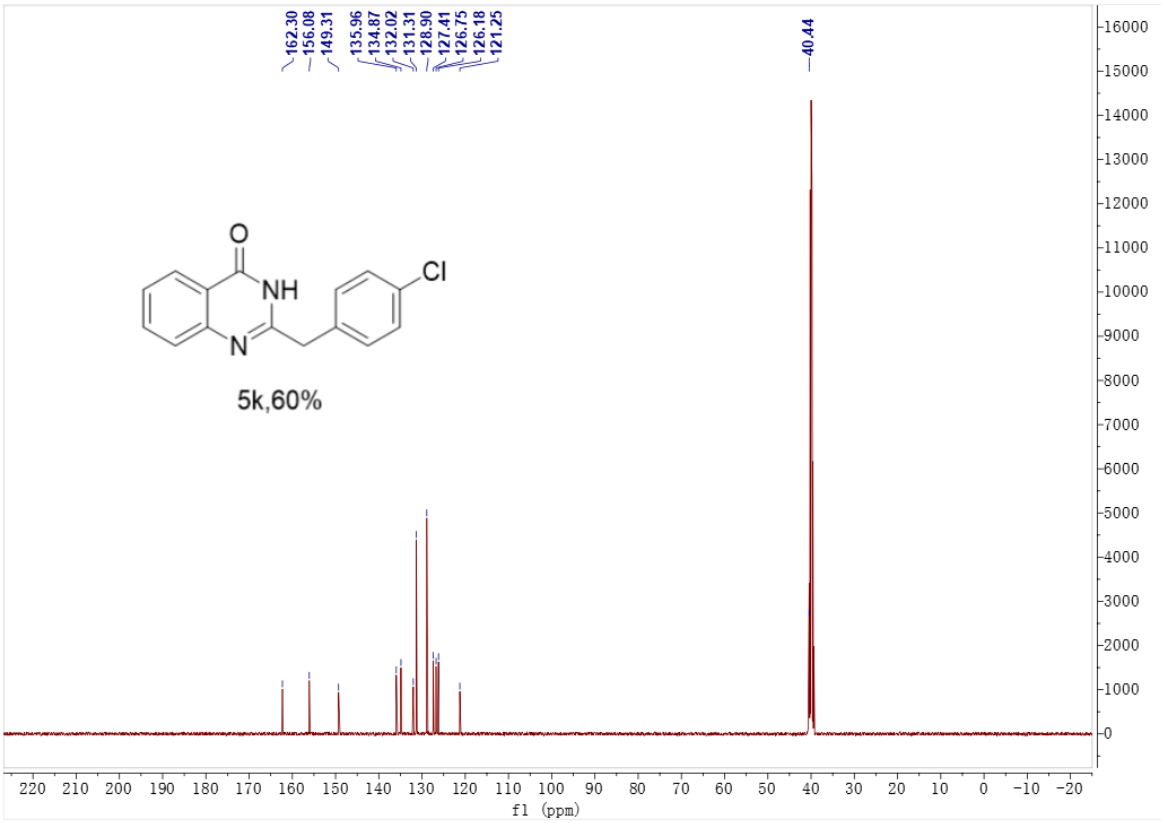

Figure S67. $^{13}C\{^{1}H\}$ NMR spectrum of compound **5k** in DMSO-d_6 (100 MHz).

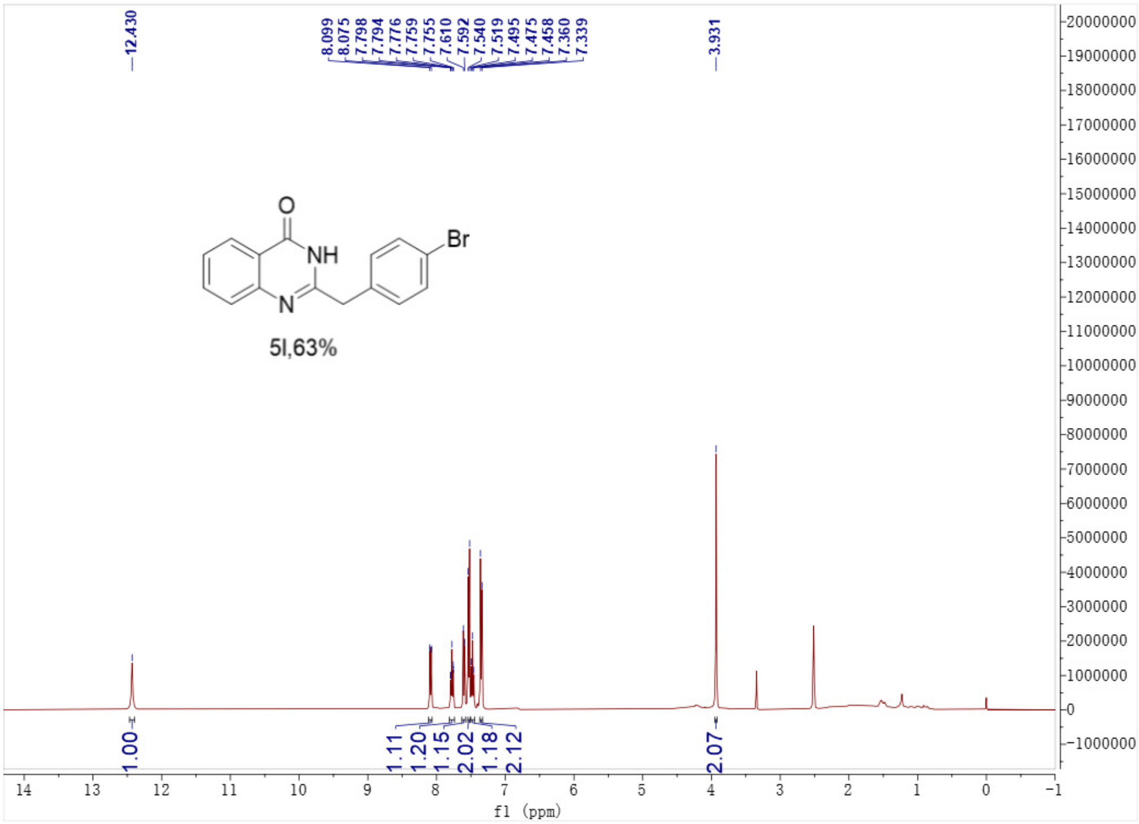

Figure S68. ^{1}H NMR spectrum of compound **5l** in DMSO-d_6 (400 MHz).

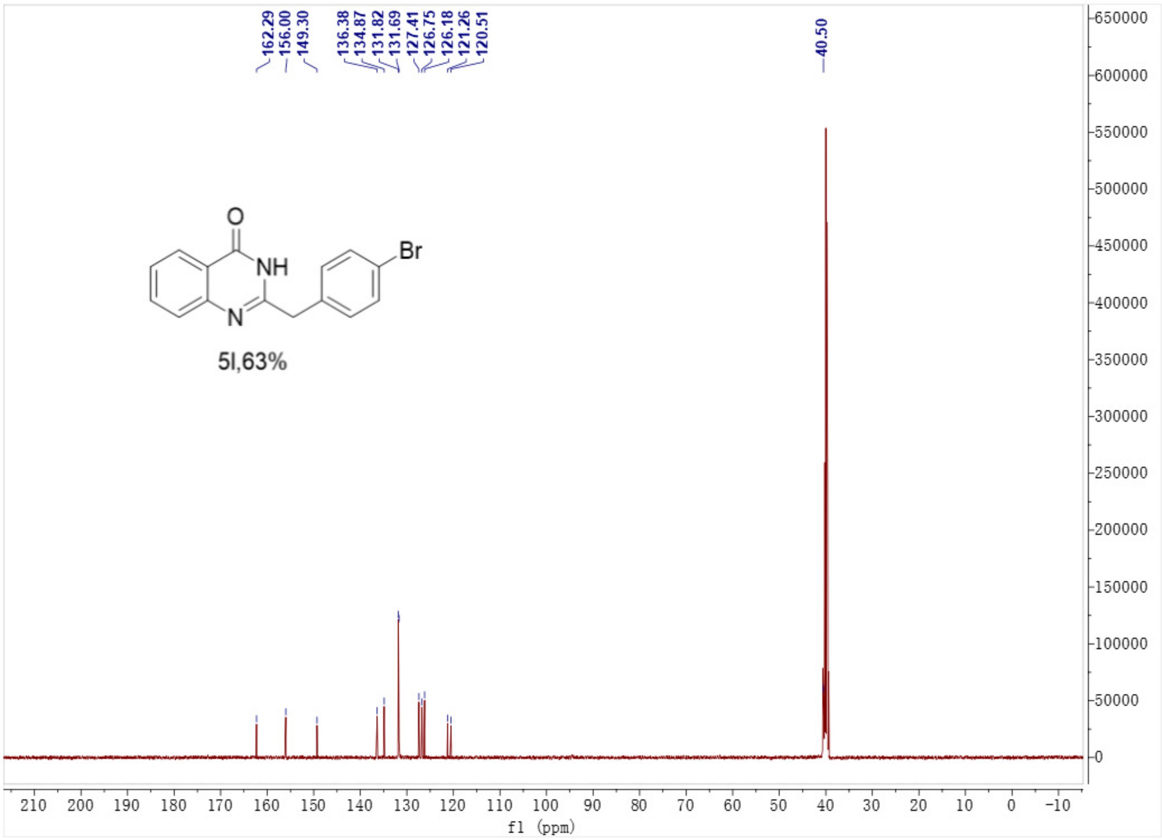

Figure S69. $^{13}C\ \{^{1}H\}$ NMR spectrum of compound **5l** in DMSO-d_6 (100 MHz).

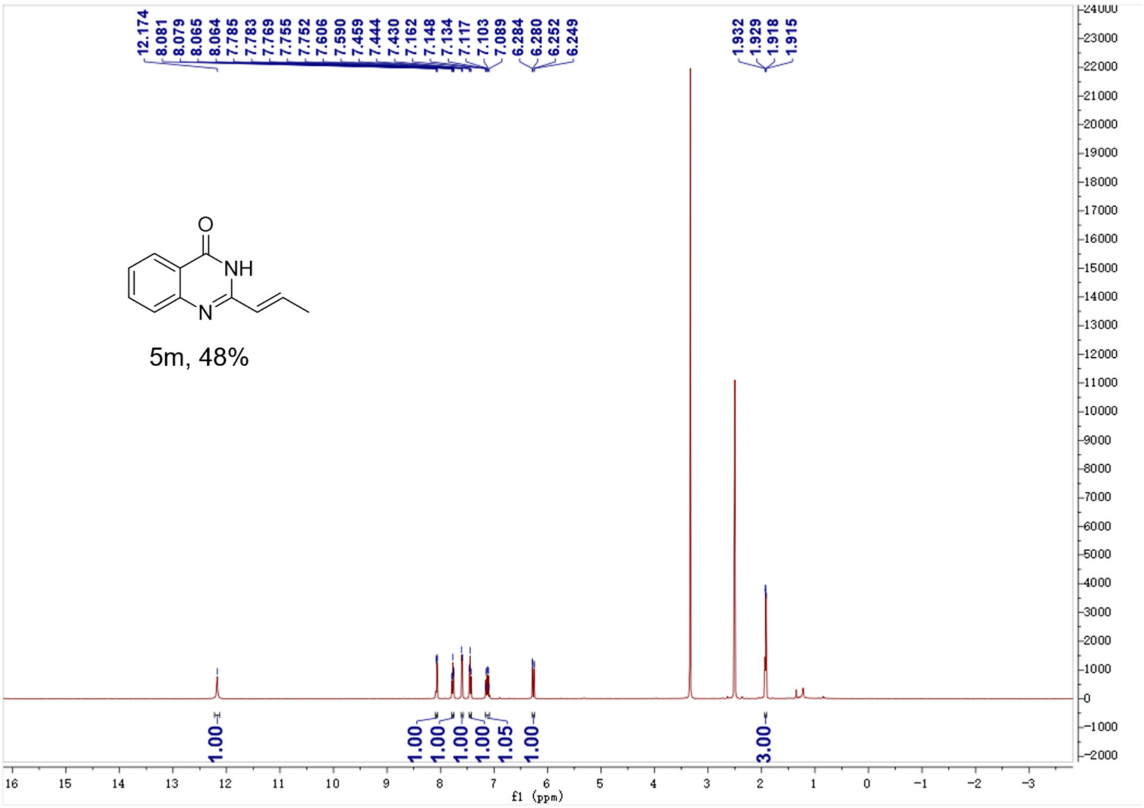

Figure S70. ^{1}H NMR spectrum of compound **5m** in DMSO-d_6 (500 MHz).

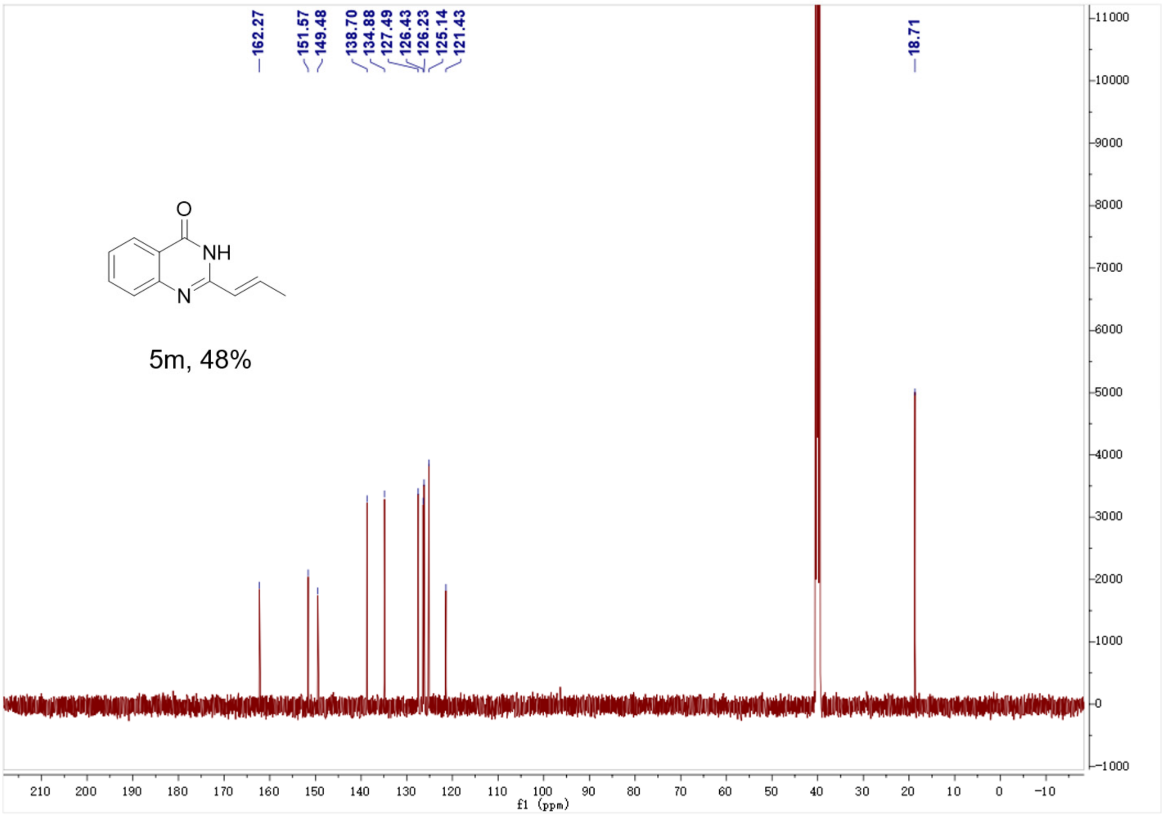

Figure S71. $^{13}C\{^{1}H\}$ NMR spectrum of compound **5m** in DMSO-d_6 (125 MHz).

2. HRMS of intermediate

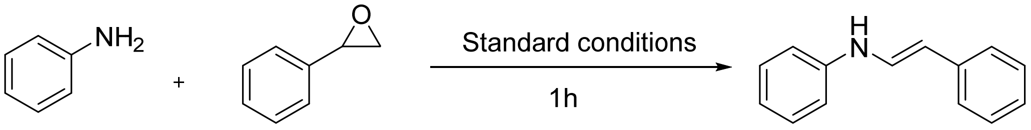

Detected by HRMS

Exact Mass:196.1120

Founded:196.1116

Display Report

Analysis Info

Analysis Name	D:\data\fenglei\LXY-K_RB1_01_15870.d	Acquisition Date	3/31/2023 4:11:42 PM
Method	1008-ms.m	Operator	Demo User
Sample Name	LXY-K	Instrument	impact II 1825265.10256
Comment			

Acquisition Parameter

Source Type	ESI	Ion Polarity	Positive	Set Nebulizer	0.4 Bar
Focus	Not active	Set Capillary	2600 V	Set Dry Heater	180 °C
Scan Begin	50 m/z	Set End Plate Offset	-500 V	Set Dry Gas	4.0 l/min
Scan End	10000 m/z	Set Charging Voltage	2000 V	Set Divert Valve	Source
		Set Corona	0 nA	Set APCI Heater	0 °C

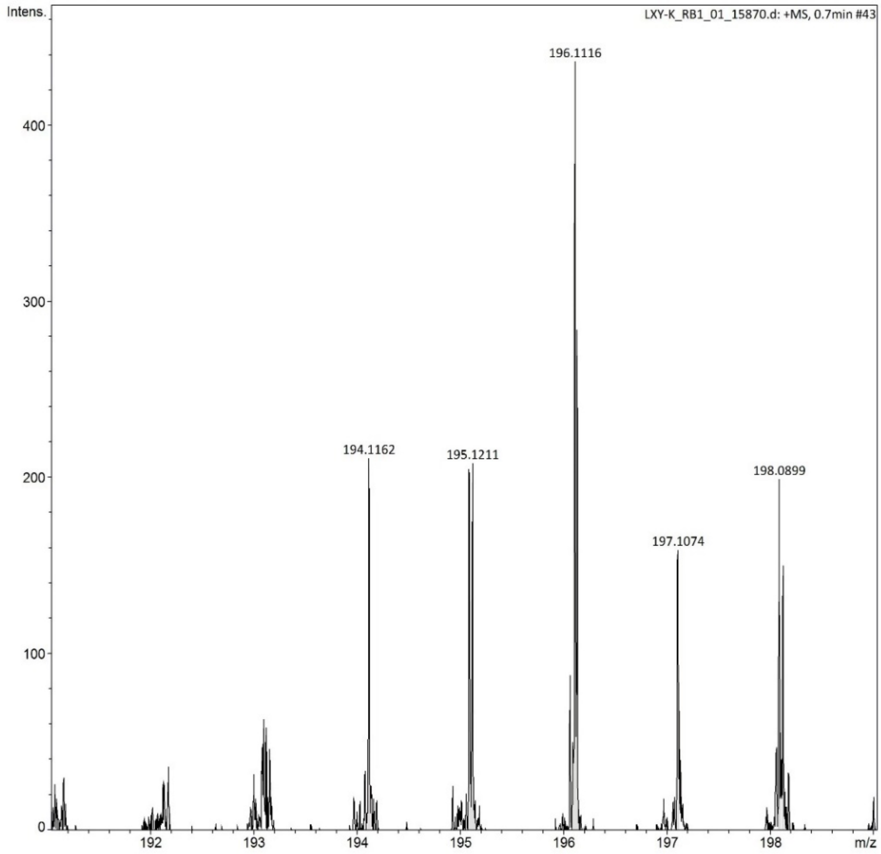

LXY-K_RB1_01_15870.d

Bruker Compass DataAnalysis 4.4 printed: 3/31/2023 4:25:32 PM by: demo Page 1 of 1

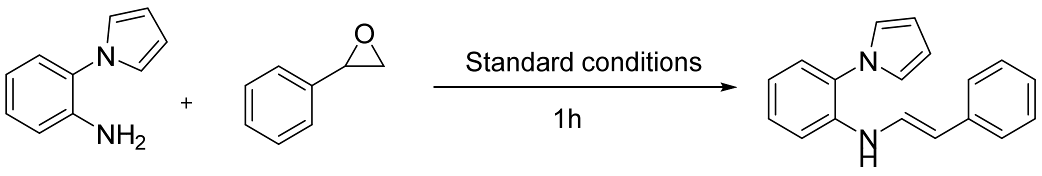

Detected by HRMS

Exact Mass:261.1385

Founded:261.1381

Display Report

Analysis Info

Analysis Name	D:\data\fenglei\LXY-Z_RB1_01_16079.d	Acquisition Date	4/4/2023 6:10:41 PM
Method	1008-ms.m	Operator	Demo User
Sample Name	LXY-Z	Instrument	impact II 1825265.10256
Comment			

Acquisition Parameter

Source Type	ESI	Ion Polarity	Positive	Set Nebulizer	0.4 Bar
Focus	Not active	Set Capillary	2600 V	Set Dry Heater	180 °C
Scan Begin	50 m/z	Set End Plate Offset	-500 V	Set Dry Gas	4.0 l/min
Scan End	10000 m/z	Set Charging Voltage	2000 V	Set Divert Valve	Source
		Set Corona	0 nA	Set APCI Heater	0 °C

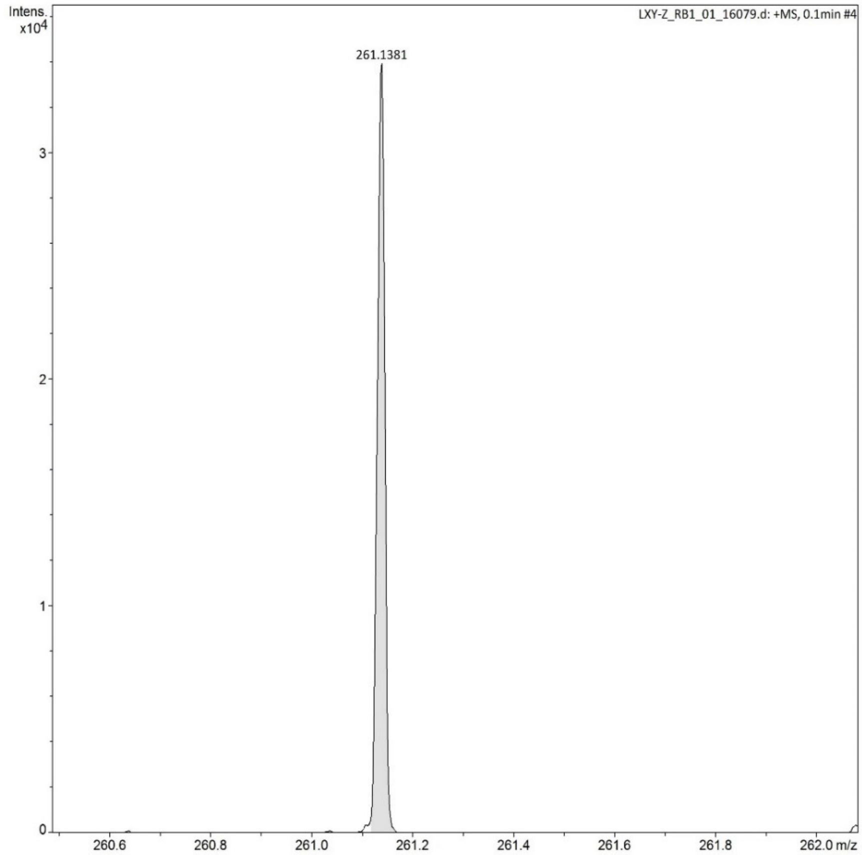

LXY-Z_RB1_01_16079.d

Bruker Compass DataAnalysis 4.4 printed: 4/4/2023 6:12:36 PM by: demo Page 1 of 1

Figure S72. HRMS of intermediate
